# Recent advances in fluorescent probes for diagnostics and targeted therapeutics at the biomedical frontier

**DOI:** 10.1038/s44386-026-00082-1

**Published:** 2026-11-14

**Authors:** Pinaki Bhattacharjee, Malliga R. Iyer

**Affiliations:** Section on Medicinal Chemistry, National Institute on Alcohol Abuse and Alcoholism, National Institutes of Health, Rockville, MD USA

**Keywords:** Biological techniques, Biophysics, Biotechnology, Chemistry, Optics and photonics

## Abstract

Fluorescence probes have contributed immensely to the understanding of cellular dynamics, pathology, and complex molecular interactions of biomolecules in living systems. There has been a resurgence in fluorescence imaging field mainly due to breakthroughs in advanced high-resolution techniques, thus propelling the development of innovative probes with superior therapeutic potential. This review emphasizes the design of fluorescent probes and the significant advancements in diagnostic modalities over the last five years.

## Introduction

The progress of fluorescence probes in the past several decades has attracted significant attention. Fluorescence signaling offers non-invasive and non-harmful real-time imaging with exquisite spectral resolution in living systems, which is particularly advantageous in modern biological research. The use of fluorescence probes has significantly helped to advance the understanding of cell dynamics, pathological mechanisms, and biomolecular interactions in their natural environments^1^. The live cell imaging market growth is primarily attributed to the advancements in imaging technologies, expanding use in drug discovery and development, and increasing demand for personalized medicine. The need for the next generation of fluorescence probes is on the rise, and the advancements observed in the application of fluorescent probes, ranging from simple imaging labeling to sophisticated systems, is an important achievement, especially for the domain of precision medicine. Early fluorescent dyes like fluorescein, rhodamine, and cyanine derivatives were initially utilized solely for tissue labeling and staining of biomolecules, such as DNA and proteins^2–5^. Although these fluorescent dyes were incredibly valuable for initial cell biology and light microscopy, they were constrained when it came to specificity, insufficient stability in vivo, increased sensitivity towards photobleaching, and high backgrounds arising due to continuous emission^6–8^. With progress in molecular biology and the growing need for targeted technologies, scientists started designing conjugated fluorescent probes—where model dyes were chemically coupled with antibodies, peptides, or ligands with high specificity for certain cell surface markers^5,9,10^. Despite these developments, signal-to-noise ratio and their application in vivo still remain problematic, mainly due to nonspecific interactions and signal generation due to unbound probes^11^.

The development of activatable fluorescent probes is an important innovation in the scientific community. These smart probes showed enhanced fluorescence only upon reacting with specific biological stimuli, such as enzymes linked to disease, pH or redox-related alterations, or markers of oxidative stresses^12^. Probes that are activated by protease cleavage by matrix metalloproteinases (MMPs) or by caspase enzymes, allow the accurate localization of cancer invasion or apoptosis, respectively^13^. This has led to a shift from the use of fluorescent probes as mere indicators to the use of fluorescent probes as highly sensitive sensors that can be used to detect real-time biochemistry in the living system. Further, these ratiometric, activatable probes have the ability to change their fluorescence wavelength in response to the stimulus, which allows quantitative measurements of the pH, ion, or redox environment of the subcellular compartments^14^. These developments have significantly clarified the molecular underpinnings of a broad range of diseases, such as cancer, Alzheimer’s disease, and cardiovascular disorders^15–17^.

With improvements in nanotechnology, the next evolutionary step has been the development of fluorescent nanocarriers for use in drug delivery systems^18,19^. Among these, liposomes, micelles, dendrimers, and polymeric nanoparticles have been endowed with therapeutic payloads and fluorescent markers^20–22^. These platforms are conducive to in vivo tracking with fluorescence imaging, allowing scientists to track distribution, pharmacokinetics, and targeting to tumors in real time. Besides this, many of these nanocarriers have also been made responsive to stimuli, releasing their therapeutic payload only under specific physiological conditions, for example, the acidic microenvironment of tumors or increased enzymatic activity^23–25^. These design elements enable localized delivery of the drug, which, in turn, minimizes side effects and augments therapy. The convergence of drug delivery capabilities with fluorescent imaging modality has provided the basis for an idea known as “theranostics,” which blends diagnostics and therapy into an integrated platform^24,26–30^. Modern fluorescent agents have the potential to detect disease biomarkers, assess treatment efficacy, and enable clinical interventions with specificity impossible through other means^31^.

In the field of precision medicine, multifunctional agents are now accepted as sophisticated tools that allow for real-time decision-making and adaptive approaches based on the unique profiles of individual patients^32^. With ongoing advances in synthetic chemistry, nanotechnology, and image sciences, future probes are expected to manifest even greater sophistication with capabilities for performing logical operations, responding to a wide range of stimuli by dynamically changing functions, and delivering not only therapeutic agents, but also genetic or immune-modulating agents. The convergence of diagnostic, monitoring, and therapeutic functions in one fluorescent platform is one of the most promising areas of modern medicine, holding great promise for improving the management of multifactorial diseases with greater efficacy and fewer side effects^33^.

Fluorescence detection and imaging are achievable in vitro and through clearly defined photophysical processes such as intramolecular charge transfer (ICT), twisted intramolecular charge transfer (TICT), photoinduced electron transfer (PET), excited-state intramolecular proton transfer (ESIPT), fluorescent resonance energy transfer (FRET), and aggregation-induced emission (AIE). By using these processes, researchers can create more accurate probes with abilities for detection of changes within the microenvironment of a cell or the target protein. With the application of such methods for increasing sensitivity and specificity of fluorescence-based methods, researchers will be able to significantly improve diagnosis and monitoring of treatments^33^.

Over recent years, there has been a tremendous surge within the field of fluorescence sensing and imaging, resulting in a plethora of scientific reviews and original research articles published within a broad cross-section of biomedical sciences sectors. This rapidly growing body of literature underscores significant advancements within probe-design, methodology for imaging, and the use of such tools within therapeutic and diagnostic applications. In accordance with rapid development, our purpose is to provide a concise and timely review summarizing recent developments within fluorescence sensors and imaging technologies. The main goal of this review is to conduct a thorough analysis of modern fluorescent probes used in biomedical applications, highlighting the role played by these probes in diagnosing diseases, monitoring cells, conducting image-guided intervention, delivering drugs, and tracking treatments with in vivo imaging. In addition to reviewing different types of fluorescent probes, the review focuses on the way modern fluorescent probes are designed to target markers that characterize various diseases, monitor biological events occurring within cells, distinguish pathologically altered tissue, and help make decisions based on biomedical information obtained through fluorescence imaging and sensing. Special attention will be paid to activatable probes, near-infrared (NIR) and NIR-II dyes, AIE fluorescent probes, fluorescent nanoparticles, genetically encoded sensors, logic-gated fluorescent probes, and novel concepts combining multiple aspects, like AI-based fluorescence imaging and wearable optical sensors. The focus of this discussion will be on the link between the probe design concept and applications in cancer, infectious disease, neurological disorders, liver injury, inflammation, and theranostics. In a nutshell, our main goal is to highlight the immense impact of fluorescence probes on the biomedical sciences, recent updates in fluorescent molecule design, clinical diagnostic technologies, and targeted therapies, and to take an in-depth look at the advances and applications of fluorescent probes in these critical areas.

## Emerging classes of fluorescent probes

Fluorescent probes can be classified in several ways depending on the focus of discussion. They are usually classified according to sensing principle, signaling response mode, target binding mechanism, fluorophore structure type, or application domain in the existing literature. In one instance, there will be classification by the mechanism for generating signals, such as PET, ICT, FRET, ESIPT, ratiometric response, or fluorescence lifetime modulation. On the other hand, the target recognition mechanism may dictate the categorization of fluorescent probes into categories such as activation of enzymes, coordination with metal ions, redox reactions, pH response, protein binding, or nucleic acid sensing^34–37^. However, the classification of fluorescent probes discussed herein is centered mainly on modern trends and recent developments. Therefore, more consideration will be given to new types of fluorescent biomedical probes, which, based on their type, can be subdivided into small molecule probes, genetically encoded probes, and nanoparticle-based probes. Small molecule probes include synthetically prepared fluorescent organic molecules used for identification of disease-relevant biomarkers, enzymes, receptors, ions, or alterations in the microenvironment. Small molecule probes possess flexibility in terms of structure, high-speed reactions, and the ability to be activatable, ratiometric-imaging, and organelle-specific imaging. Genetically encoded probes are protein sensors that can be expressed in vivo; they are useful for studying calcium signaling, neurotransmission, metabolic reactions, redox conditions, pH levels, and signaling processes within cells. Examples of nanoparticle-based probes are quantum dots, carbon dots, polymer dots, silica nanoparticles, and liposomes. They provide brighter signals, stability, long circulation period, specific target localization, and multimodal imaging possibilities. Additionally, novel approaches to designing smart probes include classification into NIR/NIR II, AIE-based probes, and logic-gate probes. They improve the depth of tissue imaging, fluorescence brightness of aggregate-containing probes, and specificity of diseases through stimuli-responsiveness.

### Small molecule activatable smart probes

**Fig. 1:**
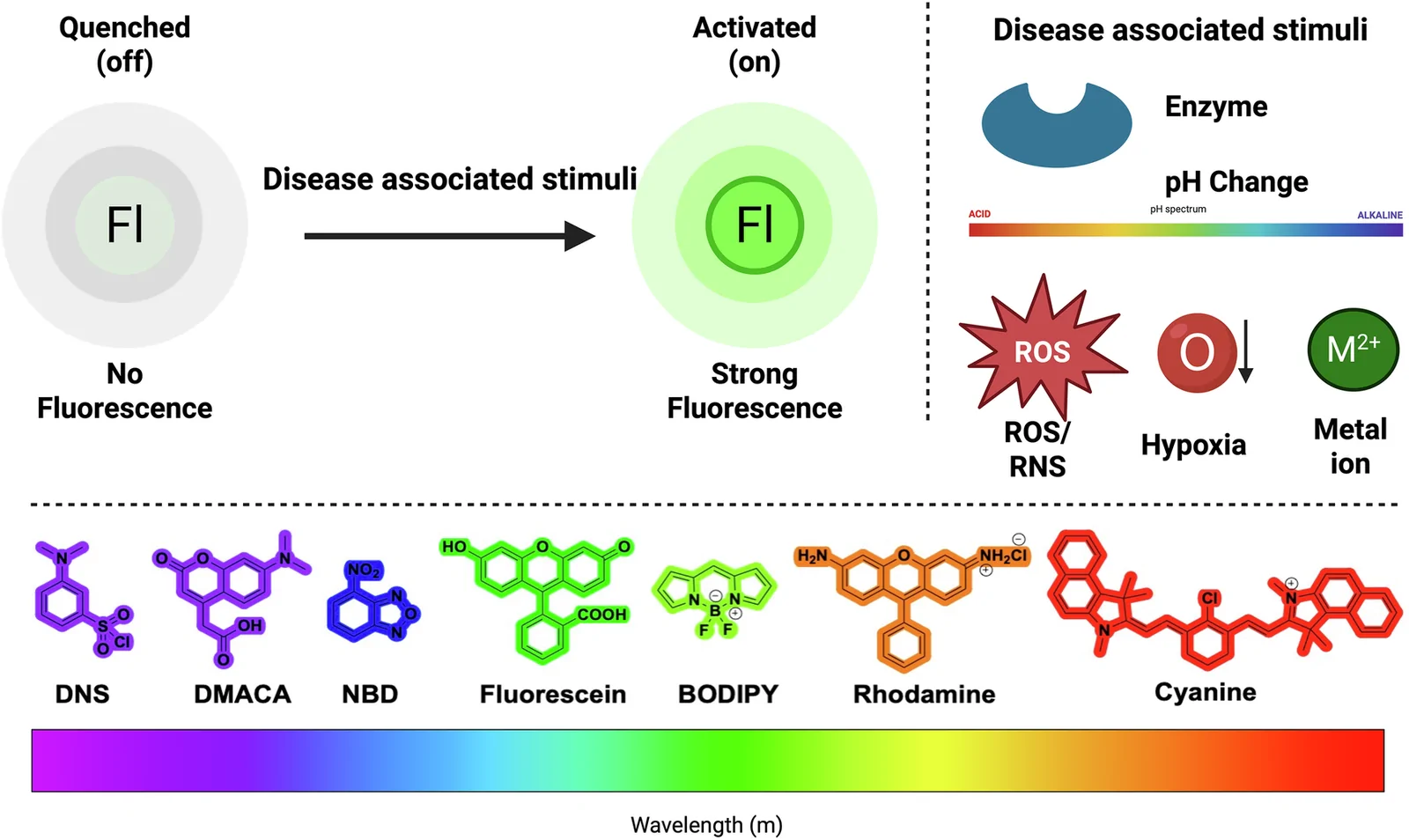
Smart fluorescent sensor mechanism of action. Various types of smart fluorescent sensors and basic mechanism of sensing. The figure was created with BioRender.com.

The use of off-on fluorescent probes that are activated by specific enzymes, pH changes, and redox states marks a remarkable milestone in molecular imaging and diagnostics^38^. Traditional fluorescent probes produce an uninterrupted signal that is insensitive to their microenvironment, which leads to significant background signals and diminished sensitivity in the molecularly complex environments of biological systems^39^. Off-on fluorescent probes are designed in such a way that they are non-fluorescent or inactive until they are activated by a specific biochemical signal (Fig. 1). Once they are activated, they are able to produce a significant signal with a high signal-to-background ratio and are capable of producing highly localized images. This characteristic of off-on probes makes them highly advantageous in in vivo studies because they are capable of producing images in a complex system such as a living body, in which nonspecific absorbance and autofluorescence are common^40,41^.

Fluorogenic and quenched probes are two essential classes of off–on systems created to maximize signal-to-noise ratios in vivo. Fluorogenic probes are generally comprised of an encapsulating fluorophore that is exposed or activated upon contact with a target, while quenched probes rely upon interruption of fluorescence quenching upon activation. Often, fluorogenic systems involve the use of self-immolative linkers, which spontaneously fragment after an initial chemical or enzymatic transformation, generating intensified fluorescence signals^42^.

In other architectures, the fluorophore is linked to an inhibitory caging group that suppresses fluorescence through electron- or energy-transfer processes; after caging group removal, fluorescence is recovered^43,44^. Quenched probes, in contrast, use dual-fluorophore or fluorophore-quencher pairs to regulate emission according to molecular proximity. FRET-based architectures are well adapted to this function, as FRET technology enables ratiometric responses—concomitantly reporting fluorophore activation state and local fluorophore concentrations. This ratiometric feature is critical for quantitative imaging, especially in living systems where fluorophore concentrations may vary due to pharmacokinetics or tissue distribution^45^. In essence, the development of fluorophores that can be used to sense enzymatic activity, pH, or redox conditions has greatly impacted the field of in vivo imaging by offering high specificity, sensitivity, and functional imaging capabilities. These probes have become indispensable in the field of biomedicine, pharmacology, and diagnostics, owing to their ability to eliminate background interference and offer real-time imaging of transient processes.

#### NIR and NIR-II fluorophores

One of the greatest improvements in fluorescence imaging over the past decade is the introduction of probes that enable improved penetration of tissue while reducing background noise, both of which are necessary to attain high-contrast, clinically relevant in vivo imaging. Although traditional fluorophores are essential for use in in vitro assays, their utility is severely constrained when applied in living systems. Most traditional fluorescent dyes, which emit light in the visible wavelength range, are bound by superficial tissue penetration due to light scattering, hemoglobin and water’s absorbance characteristics, and tissue autofluorescence, which peaks in the visible range of wavelengths (400–650 nm)^46^. These limitations have the effect of diminishing image clarity and penetration depth, especially when attempting to visualize targets embedded in dense tissue or organs^47,48^. Against these limitations, scientists have turned their efforts toward the NIR, more particularly the NIR-I (650–900 nm) and NIR-II (1000–1700 nm) wavelengths^49–51^. Emission in these wavelengths offers several benefits, such as lowered light scattering, minimal tissue autofluorescence, and increased tissue penetration—potentially several centimeters, depending upon wavelength and tissue type. This entry into these wavelength ranges has spurred further growth in organic and inorganic fluorophores tailored specifically towards in vivo imaging use^52,53^. The fundamental emissive nuclei of the majority of small-molecule organic fluorophores are based on important structural scaffolds, including cyanine, boron dipyrromethene (BODIPY), benzobisthiadiazole (BBTD), xanthene, and cyano-based derivatives^54^. Cyanine dyes, such as Cy5 and Cy7, and their analogs, are recognized as critical constituents among organic fluorophores that are applied towards near-infrared imaging pathways^55^.

Cyanine dyes are known for their unique qualities as fluorophores for use within the near-infrared range and are of growing interest since the indocyanine green (ICG) was approved for clinical use by the Food and Drug Administration in 1959^56^. The widespread use of cyanine dyes for deep tissue imaging, photodynamic therapy, and image-guided tumor resection is due to their built-in strengths, which are low toxicity, high absorption coefficient, high brightness, and tunable photophysical features^57,58^. Several other BODIPY-derived NIR fluorescent probes have been reported in the literature, owing to the favorable photophysical properties of BODIPY, such as high quantum yield, excellent photostability, and tunable emission wavelengths. The biocompatibility and synthetic versatility of the probes continue to stimulate significant research activities in the fields of bioimaging and theranostic applications^59–61^. Further development of NIR fluorescent probes identified a biomimetic approach toward constructing near-infrared-I/II fluorescent proteins (NIR-I/II FPs) with potential applications for use in vivo imaging. The researchers use protein-bound synthesized NIR-II chromophores that participate in covalent complexation with proteins, for instance, human serum albumin (HSA), through a nucleophilic substitution process. The approach makes the chromophores brighter, photostable, and biocompatible, hence allowing for better NIR-II lymphography and angiography. The process has great potential for the development of spectrally discriminated NIR-I/II FPs for use in real-time observation of bioprocesses. The process is also expected to revolutionize FP-based bioimaging and targeted delivery of albumin for deep-tissue imaging of living organisms^62^.

Fluorescent probes based on BBTD benzo-bis(1,2,5-thiadiazole) are another prominent class of organic NIR-II imaging agents. The significant electron-accepting property of BBTD allows the development of D–A–D type molecules with narrow band gap emission in the NIR-II region for their applications in deep-tissue biomedical imaging. Early examples, like CH1055, demonstrated the potential of small molecules as efficient NIR-II imaging agents, featuring rapid excretion from body fluids via kidney filtration^63^, whereas Q4/SCH1100 is an example of receptor-targeted BBTD-based probes for prostate cancer imaging^64^. Nonetheless, most BBTD-based probes still stay in the preclinical stage because of several limitations, including low water solubility, relatively poor quantum yield in aqueous solutions, long-term distribution studies, slow clearance of nanoparticle formulations, and the need for translation to clinics^65^.

The conventional family of xanthene fluorophores, e.g., fluorescein, rhodamine, and rhodol compounds, has gained wide popularity because of high brightness, high absorption coefficients, photostability, and easy synthetic modification. Modern xanthene chemistry expands this approach by providing far-red, NIR, and NIR-II imaging capabilities via heteroatom substitution, extended π-systems, and spiro-lactam fluorogenic dyes. Silicon-rhodamine compounds and other heteroatom-substituted xanthene structures offer great potential for live-cell and super-resolution microscopy imaging due to their redshifted emissions combined with fluorogenic activation properties and excellent biological behavior^66^. As an example of current achievements, one can mention the recently reported XM-Glu compound, which is an NIR xanthene dye capable of converting γ-glutamyl transpeptidase activity associated with tumors into fluorescence signals in vivo^67^. In summary, all these dyes still maintain considerable importance in biomedical research, as they provide a unique platform combining classic fluorescence chemistry and modern disease imaging approaches.

#### AIE probes

AIE is a revolutionary phenomenon in fluorescent probe technology, with especially prominent effects in the fields of biomedical imaging and applications in drug delivery^68^. Unlike traditional fluorophores, which often suffer from aggregation-caused quenching (ACQ) at high concentrations or solid-state, AIE-active compounds show the reverse behavior where they are non-emissive or show weak fluorescence at dilute solutions, but they become strongly luminescent with aggregation^69^. This apparently contradictory behavior, understood in the early 2000s, is due to the restriction of intramolecular motions (RIM), rotations, and vibrations, which in solution normally cause dissipation of the excitation energy through non-radiative paths. When these are blocked upon aggregation, there is increased radiative decay and consequent fluorescence. In the last decade, and especially in the past 5-year period, AIE-based probes have shown remarkably high efficacy in drug delivery systems, as their unique photophysical characteristics synergizes with living environments to provide sensitive and real-time monitoring of drug distribution in an organism, processes of cellular accumulation, and effects of therapeutic treatments^70^. The foremost advantage of aggregation-induced emission luminogens (AIEgens) in the realm of drug delivery is their ability to produce enhanced fluorescence when embedded in nanocarriers or bound to cellular targets^71^. Encapsulated compounds in traditional drug systems, ranging from liposomes, polymeric micelles, dendrimers, and silica nanoparticles, tend to form aggregates due to increased local concentrations and restrictive spaces^72–75^. This aggregation leads to quenching of the fluorescence, which, in turn, compromises the imaging performance of traditional dyes. On the contrary, AIEgens show an enhanced response under these conditions with high-intensity fluorescence, making possible the imaging of nanocarriers and allowing real-time tracking of these in biological environments. The effectiveness of AIEgens is thus considered highly advantageous for the detection of drug-containing nanoparticles as “turn-on” reporters, offering a platform for researchers as well as clinicians to confirm the efficient incorporation of such formulations^76^.

Apart from the imaging and therapeutic potential of the AIE probes, they have also been found to have improved photostability and biocompatibility, which are two of the major concerns that need to be addressed while using the probes. In addition, photobleaching is common in the case of traditional fluorophores, which limits the application of such probes in tracking experiments. On the contrary, AIEgens show good photostability when exposed to light, thus allowing longer exposure times without any loss in signal. Moreover, many AIEgens have already been proven non-cytotoxic and well-tolerated in vivo, with favorable biodistribution and clearance profiles^77^. Significant developments in the design of molecules have led to the synthesis of diverse AIE probes incorporating donor-acceptor architecture, heavy atoms for near-infrared emission, and biodegradable spacers. NIR-emitting probes are particularly beneficial for imaging in deep tissue, as they can reduce light scattering, suppress tissue autofluorescence, and offer higher penetration depths^78^. In a nutshell, AIE probes are a major breakthrough in the field of fluorescent probes and are currently in the process of being used in the diagnosis and treatment of various diseases and in the delivery of drugs^79^.

#### Logic-gated and multi-input fluorescent probes

Besides the activation with intrinsic stimuli, scientists have also developed smart small molecule probes that respond to dual or even multi-stimulus according to the principles of logic gates (e.g., AND, OR), allowing for greater control and specificity. The process of signal transfer at a molecular level has been of particular interest for data storage and processing, driven by a desire to miniaturize devices and impart logic operations at this level. Molecular logic gates are based on converting chemically encoded data into a change in molecular shape or spectral change upon stimulation from an external input, which could be an input of a chemical, temperature, redox potential, or radiation. Up until now, a wide range of logic devices such as AND, OR, NOT, XOR, NOR, and NAND have been reported^80^. However, constructing an integrated, complex, and sophisticated logic gate at a molecular level for memory devices, keypad locks, and key encoders/decoders is still a less investigated field^81–83^.

Logic-gated and multivalent fluorescence sensors represent a promising tool to achieve high specificity in disease imaging applications. While traditional responsive sensors provide signals based on just one disease parameter, logic-gated probes need two or more factors to be present before signaling through changes in optical output; factors like enzyme activity, oxidation/reduction levels, pH values, hypoxia, generation of reactive oxygen species, or protein aggregation that are associated with the particular disease^13^. Multi-input and logic-gated fluorescent probes constitute a novel trend in biomedical imaging because numerous diseases cannot be characterized by a unique biomarker. Cancer, neurodegeneration, inflammation, liver pathologies, senescence, infections, and many other diseases involve specific changes in the metabolism, enzymatic processes, redox status, and cell environment. Single-input fluorescent probes can activate due to the presence of a single biomarker, which may also be triggered by physiological or inflammatory processes unrelated to the disease being investigated^84^. Logic-gated fluorescent probes can emit light only after the presence of at least two disease-related inputs has been established. AND logic-gated fluorescent probes activate their fluorescent output signal only when two inputs are present at the same time or sequentially. Possible combinations include two enzymes, an enzyme plus a ROS, a protein aggregate and a redox marker, hypoxia along with glutathione, pH together with redox level, tumor enzyme activity plus tumor microenvironment, and so forth. In order to achieve a dual-locked state, these molecules feature two distinct blocking units masking the fluorophore. After both units are removed (e.g., sequentially or simultaneously), fluorescent reporters are released due to self-immolation or intramolecular cyclization of conjugates^85^. Some of the most important medical applications of this strategy refer to cancer. As is known, tumors display a variety of characteristics including enzymatic aberrations, acidic pH, hypoxia, higher glutathione content, oxidative stress, and changes in metabolism. Fluorescent probes responding to a single biological factor can be activated even in inflammatory tissue, leading to incorrect diagnosis or treatment. Therefore, a dual-locked cancer probe integrates two cancer-related triggers to increase specificity and sensitivity. Due to the pathophysiology of melanomas, two different designs were created: hydrogen peroxide/tyrosinase and hydrogen sulfide/tyrosinase probes. Hypoxia/GSH-responsive NIR probes represent another interesting example of dual-trigger fluorescent molecules used in medicine because these two factors are common for most types of cancer^86^.

In addition, logic-gated fluorescence finds a wide range of applications in clinical settings. One of them includes fluorescence-guided surgeries and intraoperative visualization of tumor boundaries. It is crucial to differentiate between cancer and normal cells before removing malignant tissue to ensure efficient treatment and avoid recurrence. Dual-input sensors featuring activation by aminopeptidase N and tumor redox status allow achieving high precision in detecting the boundaries. Cascade-activated NIR fluorescent probes can also be employed for sequential activation by tumor acidity and GSH. These designs are clinically significant because incomplete removal of the tumor often leads to tumor recurrence or unnecessary excision of healthy tissue^87^. Finally, the recent advances in logic-gated fluorescent probes also include senescence imaging. Senescence plays an important role in cancer therapy, aging, fibrosis, and chronic inflammation. Nevertheless, β-galactosidase is far from being an ideal biomarker for senescence. Modern scientists suggest that β-galactosidase, along with α-L-fucosidase, should be combined in dual-enzyme logic-gated probes for improved selectivity^88^. In this way, it is possible to differentiate between normal and senescent tumor cells as well as examine their responses to senescence-inducing therapies. It will help to study the persistence of senescent cells after treatments, thus preventing recurrence and related complications^89–91^.

While the development of many logic-gated fluorescent sensors is largely limited to the preclinical stage, their basic design is consistent with the general trend towards activatable and targetable fluorescence imaging in clinical applications. However, more validation is needed before the dual-input gate probe can be applied for human use.

### Nanoparticle-based fluorescent sensor

**Fig. 2:**
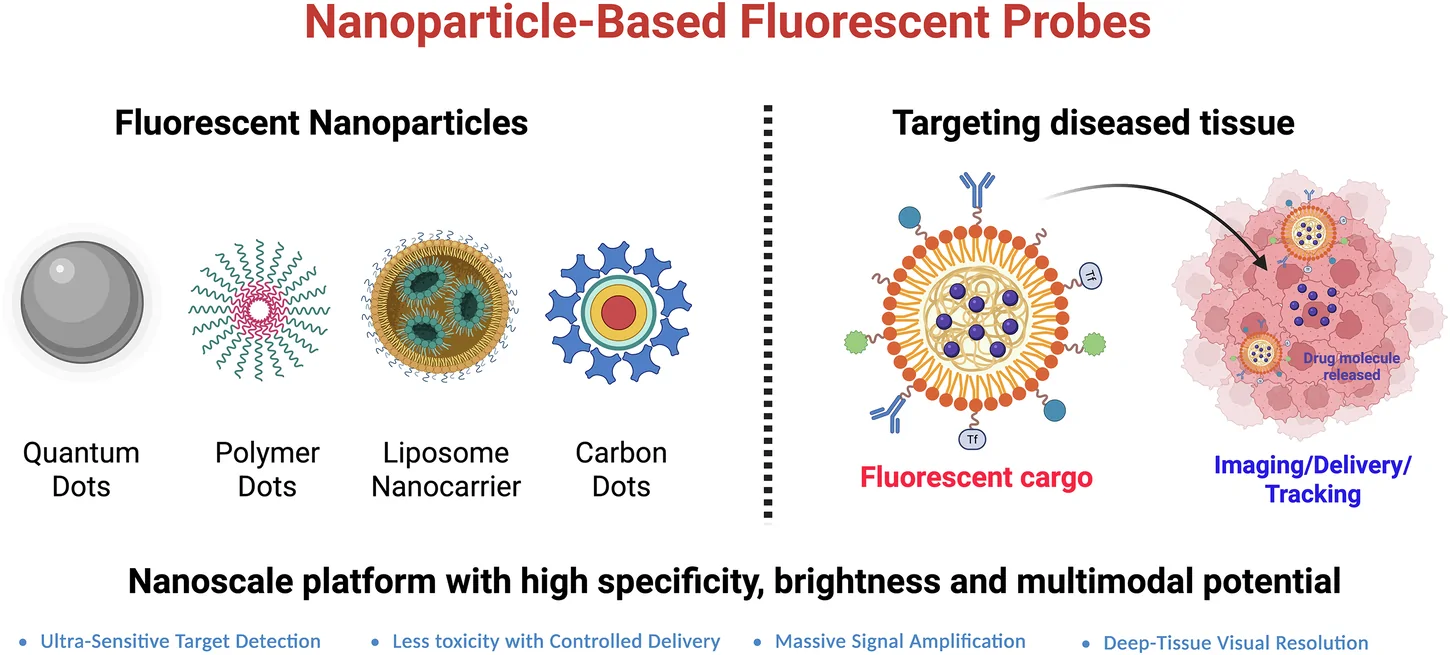
Representative nanoparticle-based fluorescent sensor. Nanoparticle-based fluorescent sensor and use in targeted therapy. Figure was created with BioRender.com.

Fluorescent sensors based on nanoparticles have become important tools for environmental studies and biomedicine, due to their unique photophysics, high surface-area-to-volume ratios, and multifunctionality (Fig. 2)^92^. The interrelation of nanotechnology with fluorescence sensing has led to a class of composite materials with greater sensitivity, ability for detection of multiple targets, and accurate measurement of metal ions, biomolecules, and even entire cells and tissues. Despite a huge number of scholarly articles being published every year on the use of nanoparticles for sensing and imaging, few move beyond the conceptual proof-of-concept phase and obtain patents, with an even smaller number eventually making it into the marketplace^93^.

Notwithstanding, even the most advanced organic dyes have at times faced limitations with respect to photobleaching and are relatively less bright compared to their inorganic counterparts. This has led to intensive study of inorganic nanomaterials, which have unique optical behavior and consistent performance in biological environments. Of these materials, the rare-earth-doped nanoparticles are especially prominent due to their excellent up-conversion and down-conversion luminescence behavior. One of the important characteristics is that these nanoparticles can absorb NIR photons of low energy and then emit higher-energy photons in the visible or NIR region. This anti-Stokes emission effectively suppresses autofluorescence backgrounds, allowing for imaging at higher tissue depths with high resolution. Rare-earth nanoparticles are usually made of a crystalline host lattice material (e.g., NaYF₄), which is co-doped with lanthanide metal ions such as Er³⁺, Yb³⁺, or Tm³⁺, which are chosen to precisely modulate the emission wavelength. The long fluorescence lifetimes and excellent photostability of these particles enable extended session imaging with minimal attenuation of the signal^94^. Moreover, surface functionalization methods such as silica shell or PEGylation have enabled them to be applied in bioconjugation and in vivo applications, including tumor imaging and delivery of pharmaceuticals.

At the core of all of these sensors are nanoparticles of different sorts, such as quantum dots (QDs)^95^, gold nanoparticles (AuNPs)^96^, upconversion nanoparticles (UCNPs)^97^, carbon dots^98^, and silica nanoparticles^99^, which serve either as carriers or fluorophores. In recent years, there is an increasing interest in dye-doped micro- and nanobeads^100–102^. A typical bead is made with many fluorescent molecules dispersed within a polymeric (such as polystyrene) or silica matrix, which increases its brightness over a conventional fluorophore tag. QDs are particularly differentiated by emission behaviors, which are size-dependent, broad absorption profiles, and excellent photostability, making them perfectly suited for extended imaging and multiplex detection.

**Fig. 3:**
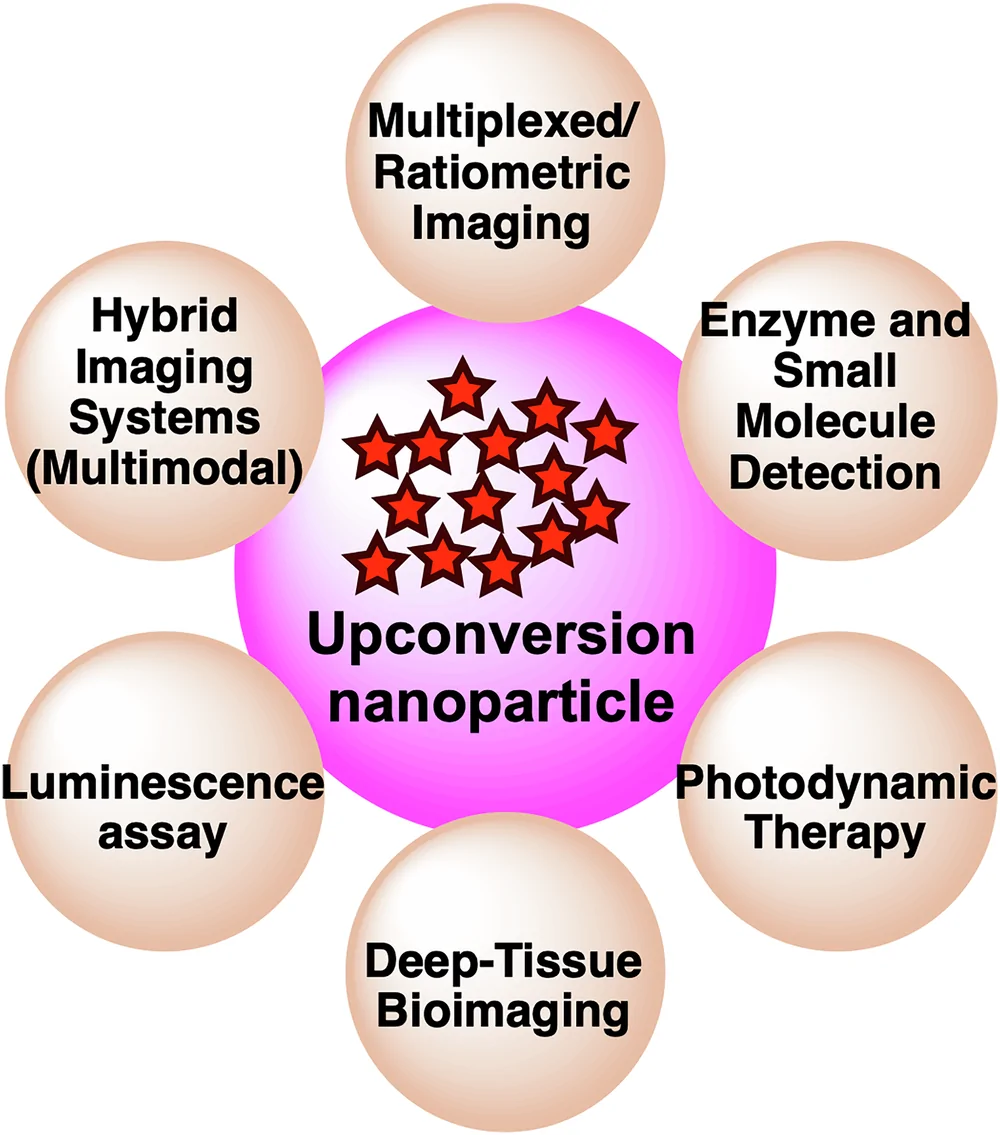
Application of upconverting nanoparticles (UCNPs). Recent studies or application on upconverting nanoparticles (UCNPs).

Similarly, UCNPs are capable of converting NIR light into visible fluorescence, allowing deep-tissue imaging with minimal autofluorescence and photodamage. Lanthanide-doped UCNPs represent a sophisticated class of optical probes with significant potential for overcoming some inherent defects of dyes, FPs, and QDs^103^. The emission process displayed by UCNPs is identical to multiphoton-excited fluorescence that is shown by traditional biolabeling agents, including dyes and QDs, because of the common aspect of converting low-energy excitation photons into high-energy emission photons (Fig. 3)^104,105^.

Recent progress in UCNP-labeled super-resolution imaging techniques, including as fluorescence emission difference microscopy, Stimulated Emission Depletion Microscopy (STED), and Structured Illumination Microscopy (SIM), has attained optical resolutions of 28 nm for individual nanoparticles and 82 nm for UCNP-labeled cytoskeletal proteins^106–109^. The detection mechanisms that enable nanoparticle-based fluorescent probes include processes such as FRET^110^, PET^111^, AIE^112^, and chemical quenching^113^. These are intended to improve sensitivity upon detection of particular biochemical or environmental cues. Nanoparticles are designed with responsive ligands or polymers that specifically interact with enzymes, ions, or reactive oxygen species, hence producing either an activation signal or a ratiometric signal. The application of nanoparticle-based fluorescent sensors in biomedical applications is witnessing rapid growth. In cancer diagnostics, nanoparticles bearing specific antibodies or aptamers are capable of specific detection of tumor markers, e.g., HER2, PSA, or folate receptors, in cancer cells^114–117^. Because of their high efficiency in fluorescence, high signal-to-noise ratio, and suitability, these are highly useful in imaging tumors in the early stages, in guiding surgeries, and in tracking responses to treatment. In neurological studies, UCNP-based probes have been designed to cross the blood-brain barrier to detect amyloid-β plaques associated with Alzheimer’s disease^118^.

### Genetically encoded fluorescent probes

**Fig. 4:**
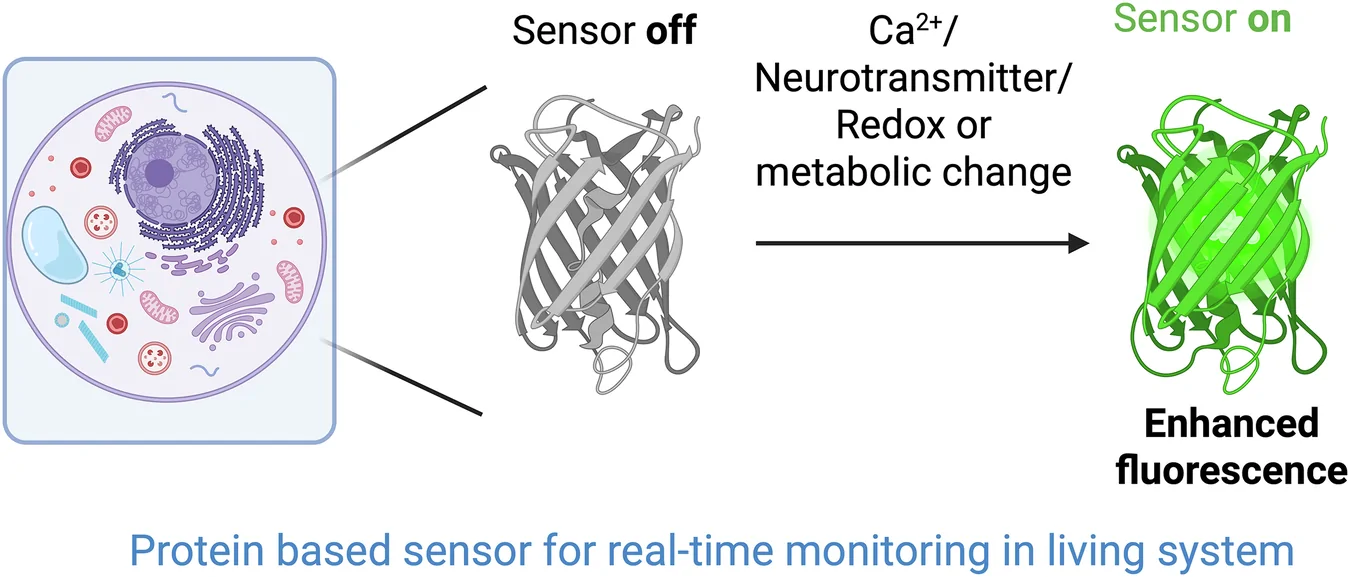
Genetically encoded fluorescent sensor mode of action. Protein-based sensor for real-time monitoring of living cells. Figure was created with BioRender.com.

Genetically encoded fluorescent sensors have revolutionized the field of molecular imaging by enabling dynamic, non-invasive, and real-time measurement of cell activities within living organisms, especially for visualizing changes in Ca²⁺ concentrations, neurotransmitters, metal ions, metabolites, pH, redox status, and signaling pathways within living samples (Fig. 4)^119^. Hence, they can be used effectively to identify molecular mechanisms underlying different diseases, ranging from cancers to neurological and cardiovascular disorders, to metabolic dysregulation and inflammation. On the other hand, from the standpoint of practical application, genetically encoded fluorescent probes are mostly valuable for validating disease-related biomarkers, conducting drug screening tests, creating disease models, and monitoring the effects of therapy on biological samples^120,121^.

Most sensors are derived from or were inspired by FP such as green fluorescent protein (GFP) and modified derivatives thereof, which are fused with protein domains or motifs designed for detection of a wide range of stimuli, such as calcium ion concentrations, redox states, pH values, metabolites, or signal molecules^122^. On target binding, a conformational change within the sensor protein alters its fluorescent properties, consequently yielding measurable readouts^123–126^.

An important class of sensors involves metabolite sensors, which are often engineered using the principles of FRET and are defined by fluorescence indicator proteins (FLIP). In a standard sensor based on FLIP, a metabolite-specific domain is strategically placed between a donor FP and an acceptor FP. Binding of the target metabolite causes a change either in the spatial distance or relative orientation of FPs, affecting FRET efficiency, leading to a change in the fluorescence ratio^121,127^. These sensors are carefully designed for glucose detection, ATP, NADH, and other intermediates of metabolism, making them a valuable tool for studying metabolic disease^128–130^. Genetically encoded redox-sensitive probes, including roGFPs and HyPer, further allow for direct detection of oxidative damage and reactive oxygen species (ROS), which are important for understanding inflammation, tumor growth, and aging^131,132^.

Genetically encoded fluorescent sensors can be categorized into several principal classes, including genetically encoded calcium indicators, voltage indicators, neurotransmitter and neuromodulator sensors, second-messenger sensors, metabolic sensors, redox and pH sensors, metal-ion sensors, enzyme-activity reporters, protein–protein interaction sensors, and organelle-specific reporters^133^. Calcium indicators like GCaMPs are extensively utilized to observe signaling in neurons, cardiac cells, and immune cells, whereas voltage indicators offer a direct visual assessment of alterations in membrane potential^134^. Metabolic and redox sensors detect variations in ATP, glucose, lactate, NADH/NAD⁺, NADPH/NADP⁺, hydrogen peroxide, and intracellular pH, rendering them essential for investigating cancer metabolism, mitochondrial malfunction, inflammation, and ischemia damage^123^. Another area of rapid expansion involves the design of genetically encoded biosensors for neurotransmitters and neuromodulators to enable real-time imaging of signaling events in functioning neural circuits^135^. In this context, FP-based ligand-binding protein and GPCR biosensors have allowed live cell imaging of glutamate, GABA, dopamine, acetylcholine, serotonin, norepinephrine, adenosine, opioids, and endocannabinoids^125,126,136–138^.

One of the strengths of genetically encoded sensors is their ability to allow subcellular imaging of dynamic biochemical events in the cellular system. Also, their genetic nature facilitates stable expression within model organisms, enabling longitudinal studies performed in vivo^131,139,140^. Also, the developments expanded upon the range of FPs available to include blue, cyan, yellow, and red variants, facilitating multiplex imaging and compatibility with advanced techniques including two-photon microscopy, super-resolution, and optogenetics^141^. Despite all of their benefits, these sensors face a multitude of challenges, including photobleaching, pH sensitivity, and potential disruption of cellular homeostasis due to overexpression. Nevertheless, continued research efforts toward protein engineering are designed with a view to enhancing their brightness, kinetics, and specificity. Despite the significant translational potential of these probes for biomarker validation, drug screening, point-of-care diagnostics, disease modeling, and monitoring of therapeutic responses, their direct implementation in the clinical setting is still currently limited.

## Disease-oriented diagnostic applications of fluorescent probes

Diagnostic imaging techniques heavily rely on fluorescent probes due to their high degree of sensitivity and modulable specificity^15^. With the burgeoning expansion in fluorescent probe investigations, it becomes unfeasible for one particular review to include all possible disease categories and probe types with equal attention. Therefore, in this particular review, only some selected but extremely relevant biomedical areas will be focused upon, which include cancer, infectious diseases, neurodegenerative diseases, liver diseases, and cardiovascular diseases. The selected fields are considered, as important since these categories are linked with high mortality rates, along with the fact that the fluorescent probes exhibit great promise in diagnosing, studying the mechanisms, intervening with image guidance, and monitoring therapy in these categories. In the specified categories, only some selected and advanced fluorescent probes will be introduced, including activatable probes, NIR/NIR-II probes, AIE probes, nanoprobes, genetically engineered sensors, and logic-gated probes. It should be noted that instead of providing a list of all kinds of fluorescent probes, the focus will be made on the design strategies as well as the applications in biomedical sciences. The applications of fluorescent probes cover oncology, infectious diseases, and neurological diseases, which enable real-time imaging and tracking of the progression of diseases^15,34^. By using well-characterized fluorescence probes for specific organisms, researchers are able to track disease progression as well as related biological processes in a living organism^33,142,143^.

### Cancer detection and image-guided diagnosis

The high cancer mortality rates can mainly be linked to the lack of characteristic morphological features of cancer within the early stages of cancer development. The lack of obvious pathological markers seriously delays the process of early detection and diagnosis, considering that traditional image techniques and histopathological examinations cannot identify subtle or pre-malignant changes. This delay in diagnosis greatly influences the ideal time to initiate treatment, thus reducing the chances of successful outcomes and resulting in poor patient prognoses^144–147^.

**Fig. 5:**
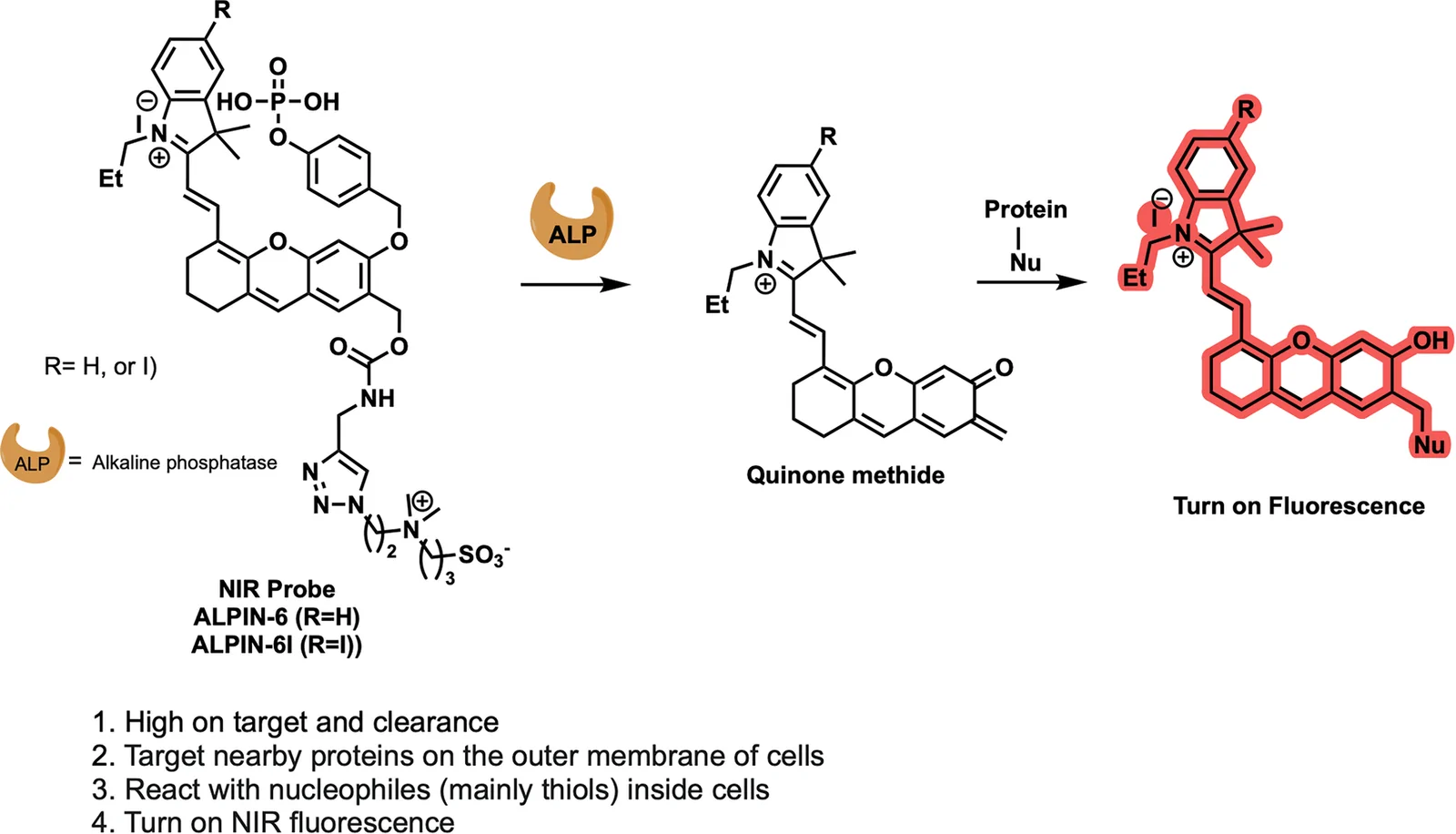
Turn off ALPIN probes converted to turn-on probes with alkaline phosphatase. Schematic illustration of enzyme-activatable near-infrared photosensitizer ALPIN-6 and ALIN-6I.

**Fig. 6:**
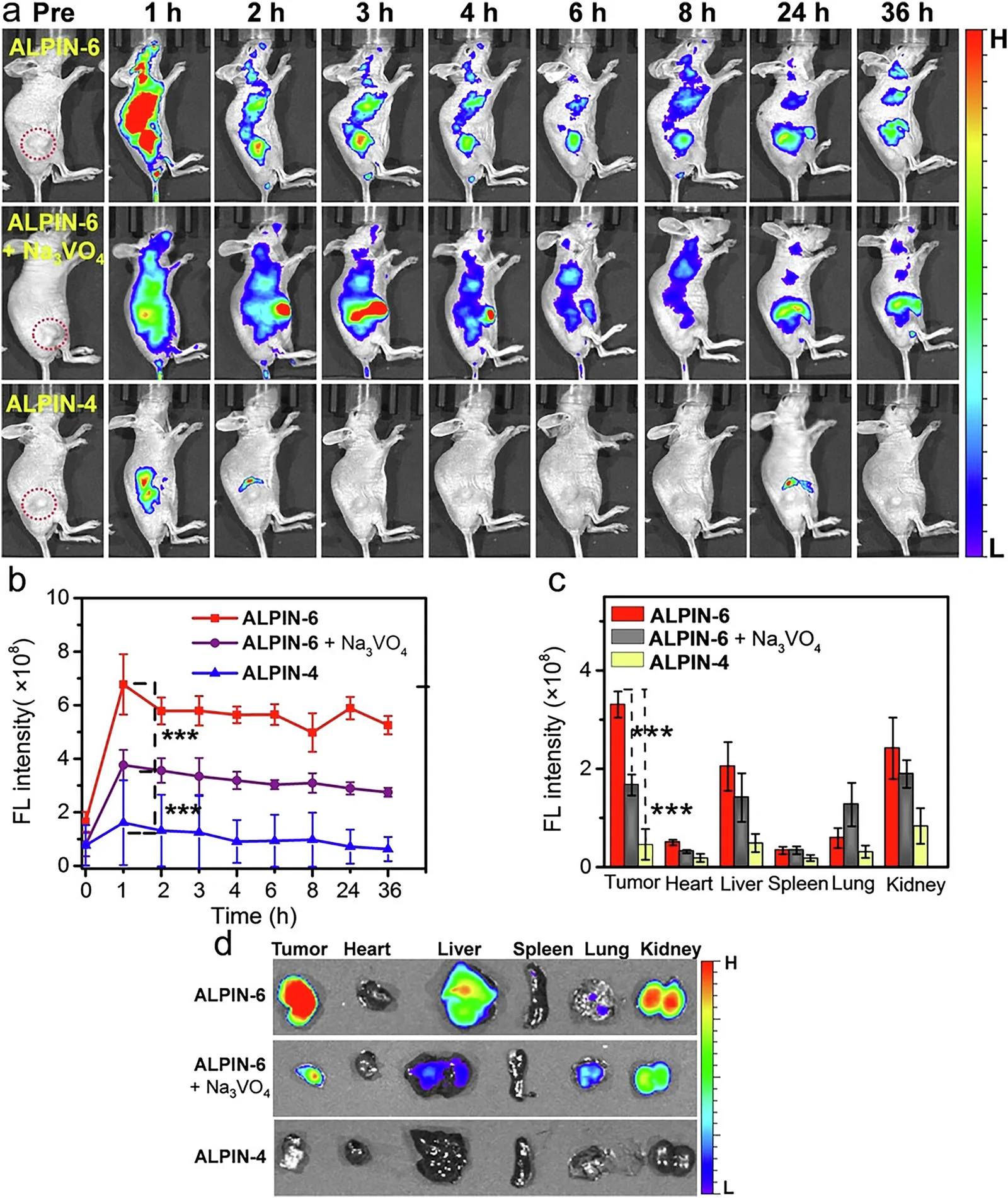
In vivo experiments with NIR probe ALPIN-6. **a** Representative fluorescence images of HeLa tumor-bearing mice following intravenous (i.v.) administration of the NIR probe. Mice received the probe alone at a dose of 0.4 μmol/kg via i.v. injection or were pretreated with i.t. injection of Na₃VO₄ (10 mM, 50 μL) before probe administration. **b** Time-dependent fluorescence intensity changes in the tumor region. **c** Quantified fluorescence intensities of tumors and major organs shown in (**d**). **d** Ex vivo fluorescence images of tumors and major organs collected 36 h after probe injection. λex/em = 660/710 nm. Data represent mean values ± s.d. (*n* = 3); ***P < 0.001. Reproduced with permission^150^.

Fluorescence imaging based on molecular fluorescent probes has been widely used in the biomedical field to monitor biological species at the single-molecule and cell levels, and in vivo because of the method’s high spatiotemporal resolution, sensitivity, and in situ and/or real-time detection in complex biosystems. Therefore, in situ activatable fluorescence imaging may be a powerful way to enable high-resolution imaging of the dynamics of enzymes in living systems^148,149^. Enzyme activity is one of the most widely studied stimuli for such probes. Recently, a novel “1-to-3” multi-effect technique has been developed for the development of enzyme-activatable imaging reagents and photosensitizers (Fig. 5). The alkaline phosphatase-activatable near-infrared probe (ALPIN-6) and photosensitizer (ALPIN-6I) transition from hydrophilic to hydrophobic following alkaline phosphatase (ALP) activation, covalently attach to proteins, and exhibit enhanced near-infrared fluorescence or photosensitizing capability. This method is suitable for real-time imaging of ALP activity in living cells and for in vivo imaging with prolonged fluorescence. The ALP-activatable photosensitizer is selectively triggered and concentrated in tumor cells that overexpress ALP, resulting in a 50% decrease in free thiols and improved PDT effectiveness (Fig. 6)^150^.

**Fig. 7:**
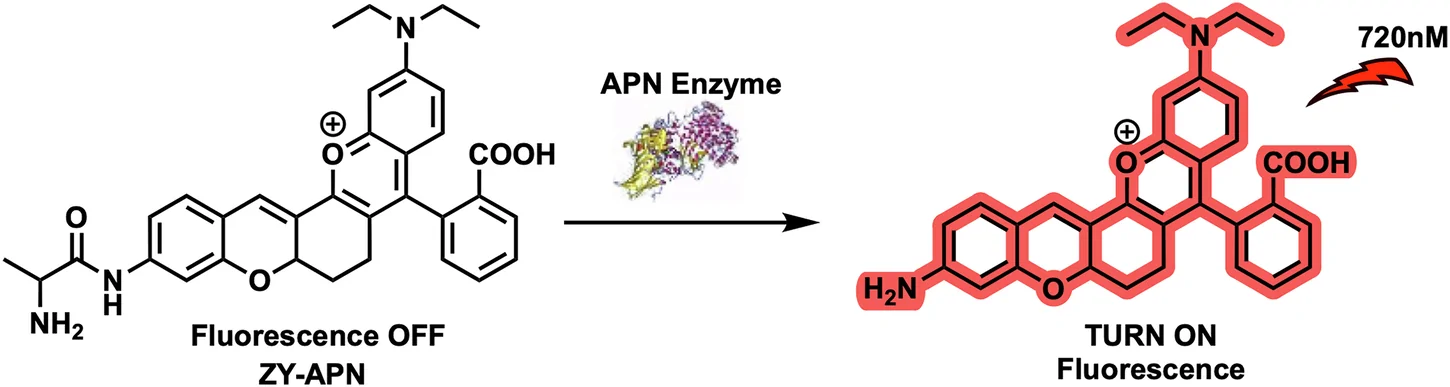
Aminopeptidase N activatable fluorescent probe ZY-APN. Schematic illustration of the NIR fluorescent probe ZY-APN for imaging enzyme activity in cells and mice.

**Fig. 8:**
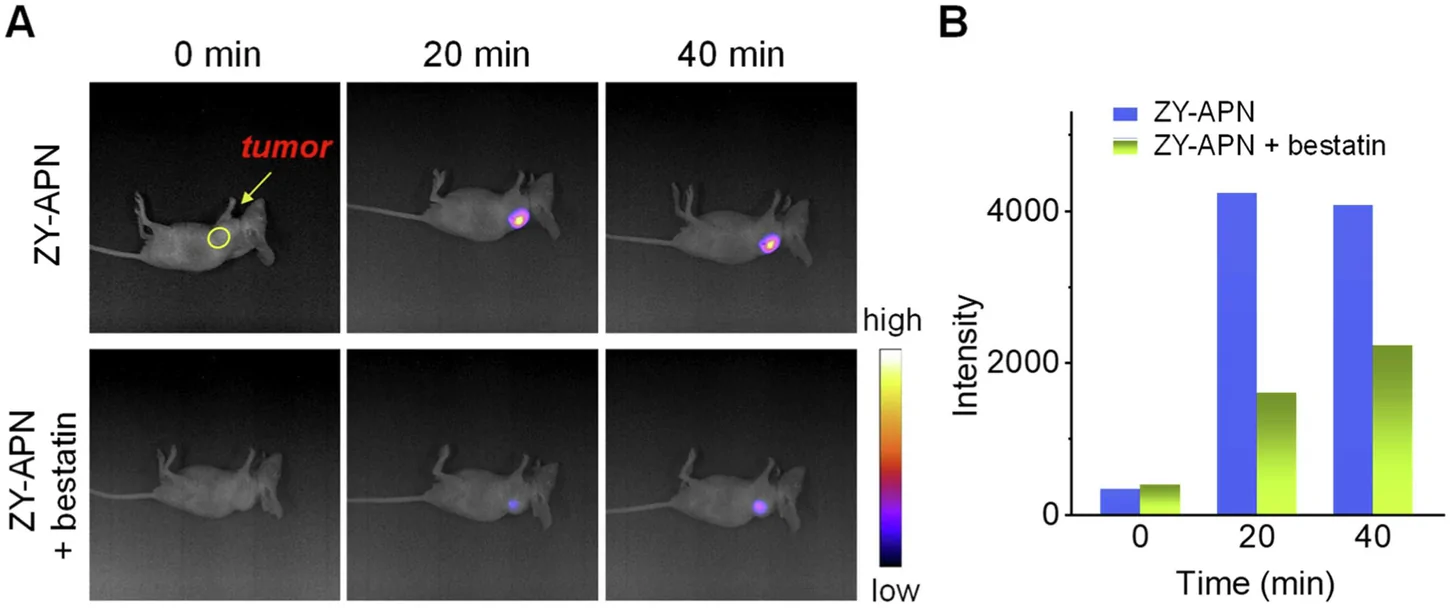
Tumor imaging of APN in vivo. **A** Fluorescence images of HeLa tumor-bearing mice. In the first group, mice received only an intratumoral injection of probe ZY-APN (50.0 μL, 200.0 μM). In the second group, mice were pretreated with bestatin (50.0 μL, 1.0 mM) for 0.5 h, followed by intratumoral injection of ZY-APN (50.0 μL, 200.0 μM). Fluorescence images of both groups were acquired at 20 and 40 min after probe administration. **B** Time-dependent changes in the average fluorescence intensity within the tumor region from 0 to 40 min, corresponding to (**A**). Reproduced with permission^152^.

Aminopeptidase N (APN) is a significant proteinase, with abnormal levels of APN closely associated with various diseases. Mammalian APN is essential in various physiological processes, including pain sensation, blood pressure regulation, tumor angiogenesis and metastasis, immune cell chemotaxis, sperm motility, and cell–cell adhesion. The precise detection and monitoring of APN activity hold substantial importance in clinical diagnosis and drug development^151^. The research introduced an APN-activated NIR fluorescent probe, ZY-APN, which demonstrated efficacy in both in vitro and in vivo enzyme detection(Fig. 7). The probe exhibited a significant enhancement in near-infrared fluorescence emission at 730 nm and was useful in the differentiation of normal and tumor cells based on the level of APN activity. The probe showed promise in the diagnosis and treatment of APN-associated diseases, especially cancer (Fig. 8)^152^.

**Fig. 9:**
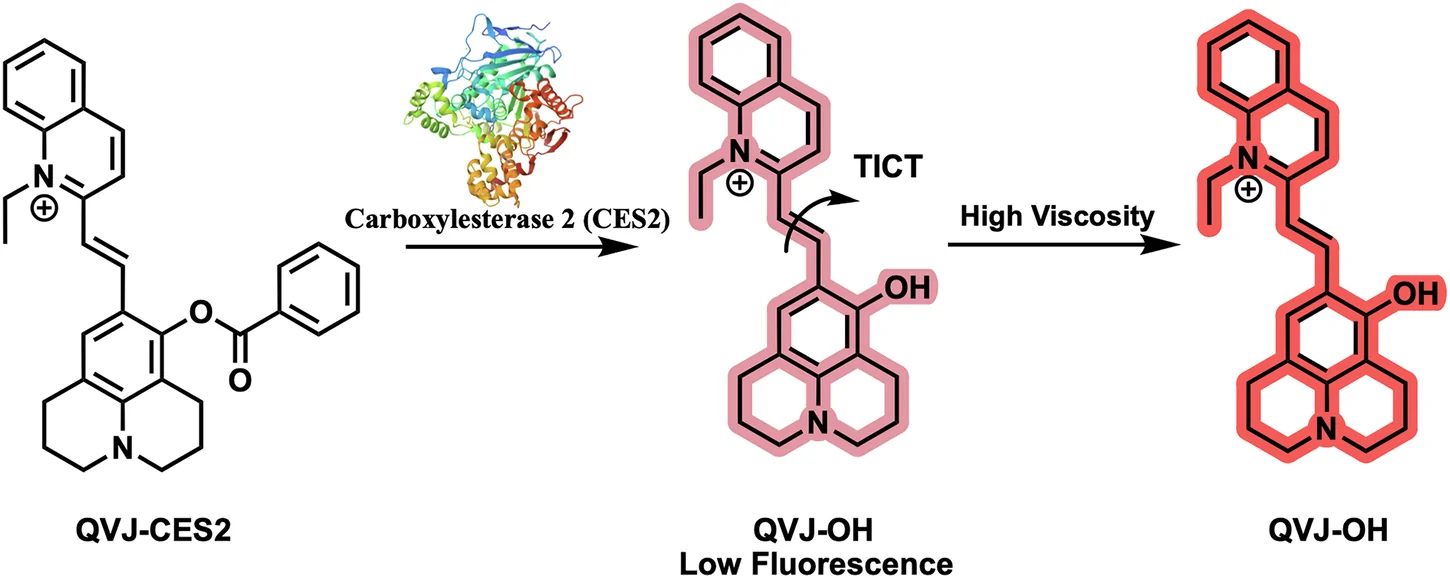
Schematic illustration of the NIR fluorescent probe (QVJ-CES2) based on carboxylesterase 2 and viscosity cascade response towards thyroid cancer detection. Detection method of QVJ-OH in the presence of carboxylesterase 2.

Another enzyme Carboxylesterase 2 (CES2) enzyme played a significant role in the metabolism of drugs containing ester and amide groups and influences their therapeutic potency and toxicity. In the study, a near-infrared fluorescence probe, QVJ-CES2, was synthesized to increase the detection of thyroid cancer, as it detects CES2 enzyme activity and cellular viscosity in thyroid cells and has a detection limit of 1.9 ng/mL (Fig. 9). It employs a dual response mechanism that increases fluorescence intensity and therefore detection sensitivity. In vitro studies showed its quick responsiveness with specificity and can therefore be used in early diagnosis of cancer and accurate surgical procedures^153^.

A significant number of pathological and physiological processes have been related to the activity of enzymes like caspases and MMPs, which are associated with cellular processes like apoptosis, metastasis of cancer cells, inflammation, and tissue remodeling^154,155^. For instance, caspase-3-activatable probes, wherein the role of caspase-3 is that of an important effector molecule in the apoptotic pathway, are extensively utilized in both the visualization and quantitation of programmed cell death in cell culture systems and model animals. Caspase-3 is a member of the intracellular cysteine protease family and plays a key role in the initiation and execution of apoptosis. These types of probes are especially relevant to the assessment of the efficacy of cancer therapy or the study of the course of neurodegenerative diseases, where apoptosis plays an intrinsic part.

**Fig. 10:**
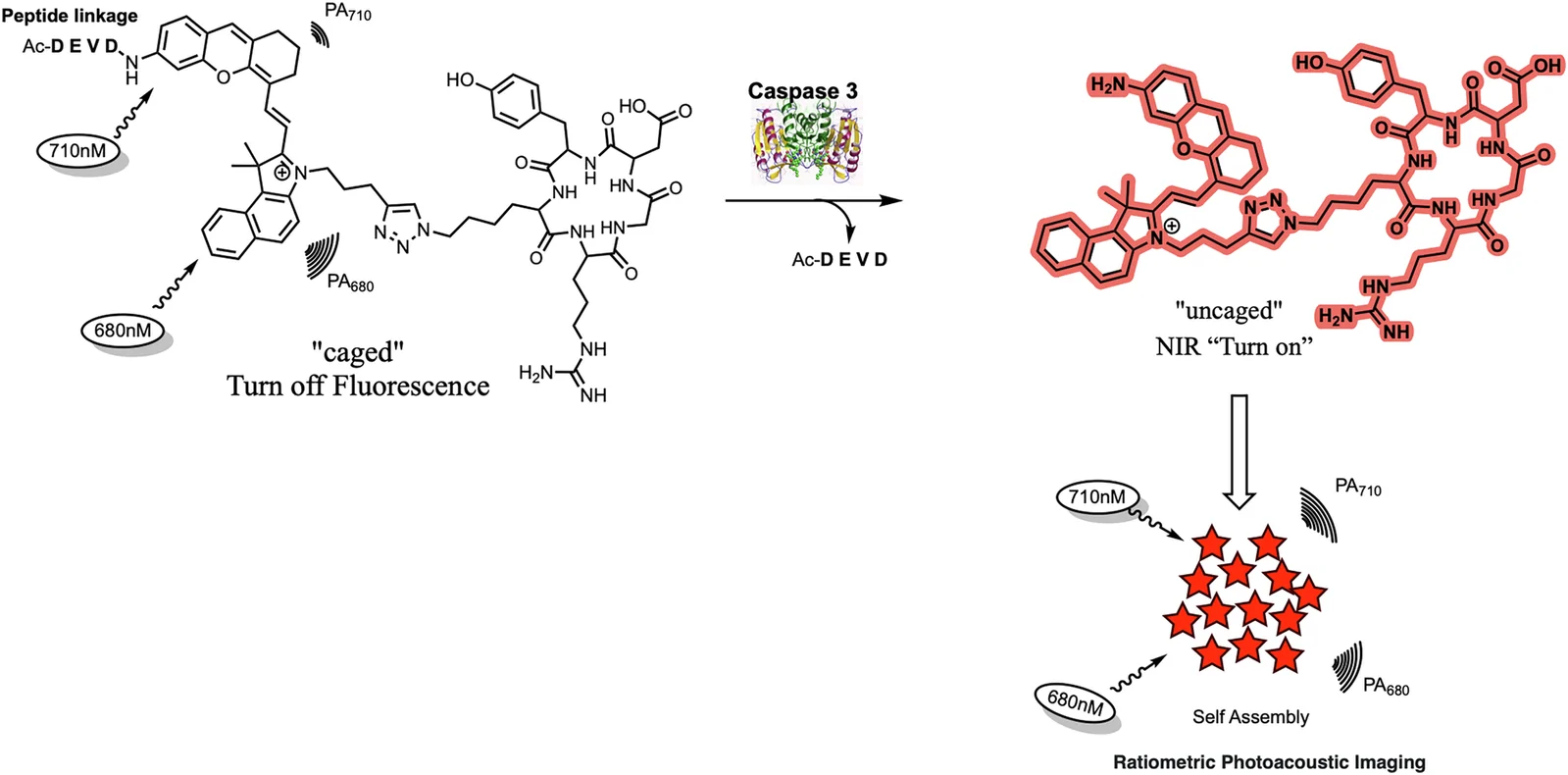
Enhanced NIR emission and in situ self-assembly responsive to caspase-3. Schematic illustration of a ratiometric photoacoustic imaging NIR probe for Tumor Apoptosis.

**Fig. 11:**
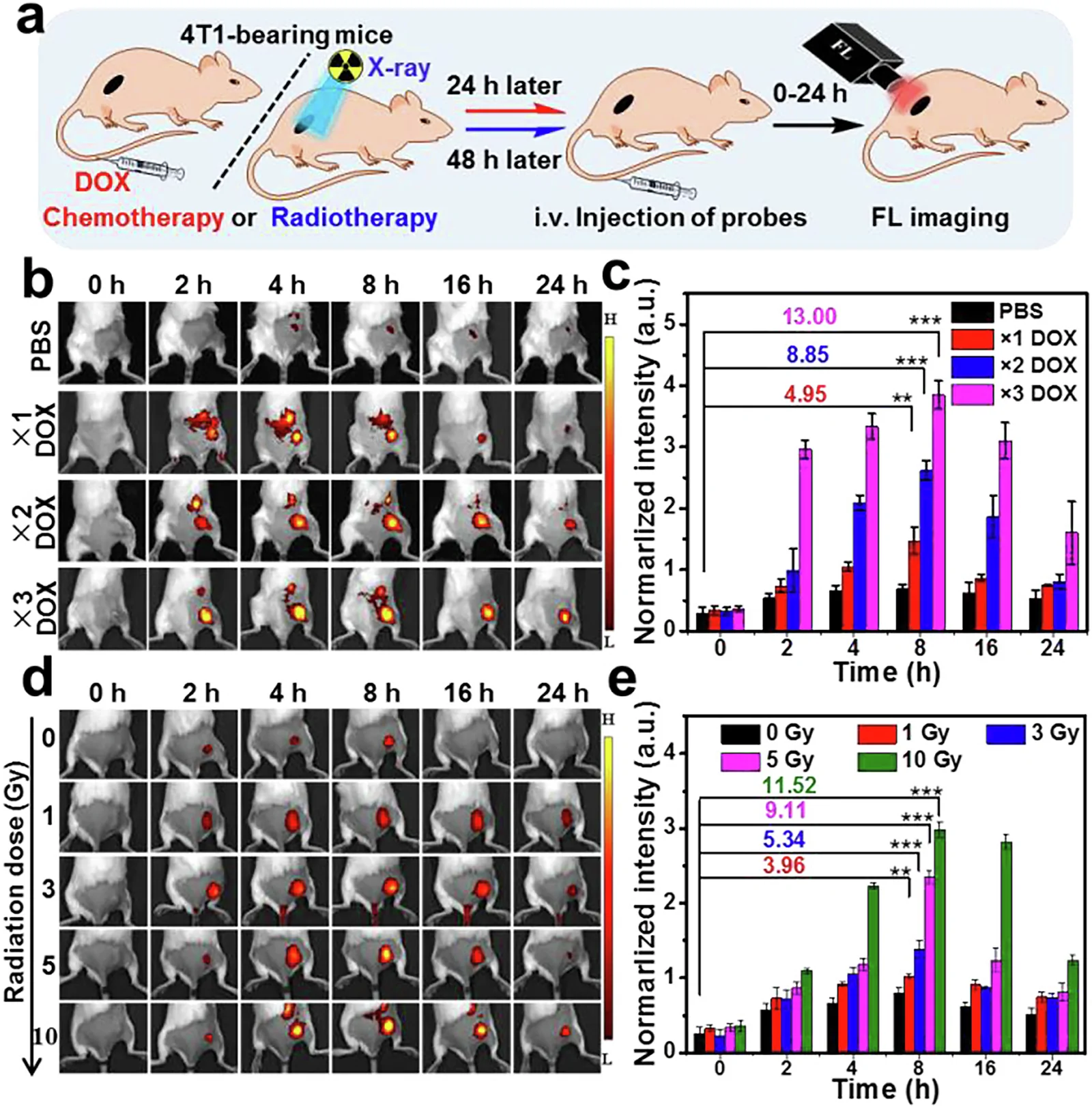
In vivo real-time fluorescence imaging of 4T1 tumors. **a** Schematic illustration showing the establishment of chemotherapy- and radiotherapy-treated 4T1 tumor-bearing mouse models, followed by fluorescence imaging. **b** In vivo fluorescence images of 4T1 tumor-bearing mice pretreated with PBS, 1× DOX, 2× DOX, or 3× DOX, after intravenous injection of the probe at a dose of 1.5 μg g^−1^. **c** Quantitative analysis of tumor fluorescence intensities under different pretreatment conditions shown in (**b**). **d** Fluorescence images of tumors exposed to different radiation doses. **e** Quantification of fluorescence intensities from tumors subjected to different radiation treatments shown in panel.**P < 0.01 (*n* = 3), ***P < 0.001 (*n* = 3). Reproduced with permission^156^.

Shi et al. successfully developed a ratiometric photoacoustic probe for tumor-targeted and caspase-3-activatable near-infrared fluorogenic imaging (Fig. 10). A newly developed molecular probe named AcDEVD-Cy-RGD consists of three active parts: (i) a Asp-Glu-Val-Asp (DEVD) peptide that is specific to the enzyme caspase-3 and acts as the responsive component, (ii) a cyclic Arg-Gly-Asp (cRGD) peptide that acts as the target component, and (iii) a hemicyanine dye that acts as the reporter component. The probe exhibits near-infrared fluorescence and ratiometric photoacoustic signals, enabling the detection of caspase-3 activity in a concentration-dependent manner. Because the hemicyanine unit’s amino group is in a “caged” form with decreased electron-donating capacity, this probe is initially nonfluorescent. In the presence of the enzyme caspase-3, the amide bond between the DEVD peptide and the dye is cleaved, leading to the rapid release of the dye NH2-Cy-RGD in its uncaged form, accompanied by an increase in near-infrared fluorescence output and significant self-assembly of the probe. This facilitates the real-time observation of tumor cell apoptosis in living organisms. The probe has been utilized to visualize tumor responses to chemotherapy and radiotherapy, facilitating precise estimation of radiation doses. The probe’s superior ratiometric photoacoustic (PA710/PA680) responsiveness positions it as a viable technique for noninvasive and quantitative assessment of caspase-3 expression and cellular apoptosis in vivo (Fig. 11)^156^.

In a recent study by Lai et al., the development of a xanthene-based near-infrared fluorescent probe, XM-Glu was developed that can efficiently analyze the γ-glutamyltranspeptidase (GGT) enzyme activity associated with cancer-related biomedical imaging. GGT is an enzyme that plays an important role in glutathione biosynthesis and is also known for its antioxidant properties. In many cases, this enzyme is associated with abnormal expression related to several cancers. XM-Glu acts as an enzyme-activatable probe where the interaction between γ-glutamyl binding site and GGT results in a near-infrared fluorescent probe with an emission wavelength of around 648 nm. This probe showed a detection limit of up to 0.067 mU mL⁻¹. The function of this activatable probe becomes important in in vivo imaging as it helps to eliminate non-specific background noise and allows for the real-time measurement of the activity of enzymes associated with tumors. The research shows that XM-Glu can identify GGT activity within tumors, the liver, and the kidneys tissues^67^.

**Fig. 12:**
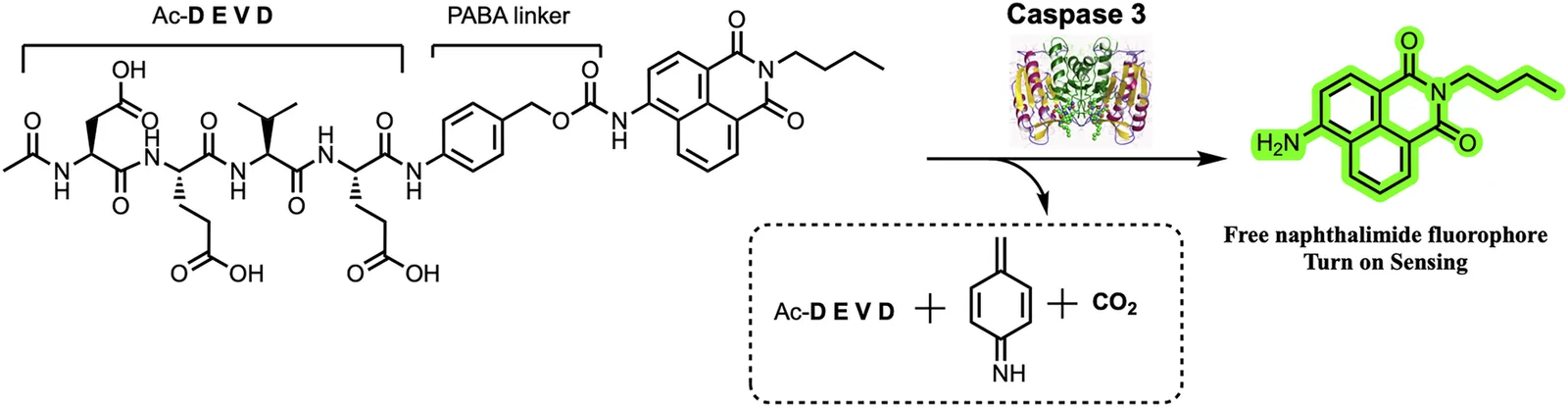
Release mechanism of 1,8-naphthalimide-based ratiometric fluorescent probe for ratiometric sensing. Schematic illustration of Ac-DEVD-PABC-Naph for the of caspase-3 visualization.

Further, a new ratiometric fluorescent probe, Ac-DEVD-PABC-Naph, has also been synthesized and characterized. The probe employs a 1,8-naphthalimide fluorophore covalently attached to a peptide sequence using a self-immolative p-aminobenzyl alcohol linker. Following enzymatic cleavage by caspase-3, the probe undergoes spontaneous degradation, liberating the free naphthalimide fluorophore and causing a ratiometric alteration in fluorescence emission. Spectroscopic investigations revealed a time-dependent ratiometric fluorescence response, indicating significant sensitivity to caspase-3 activation. Enzyme kinetics parameters, including Km, Kcat, and limit of detection (LOD), were acquired, indicating that the probe exhibits kinetic data similar to previous probes documented in the literature, while further offering the advantages of ratiometric detection (Fig. 12)^157^.

Similarly, MMP-sensitive probes are often designed to contain substrates that are cleaved by MMP-2 or MMP-9, enzymes that are overexpressed in invading tumors and inflammation sites. These kinds of probes are useful in visualizing the microenvironment of the tumor or the early diagnosis of pathological conditions like rheumatoid arthritis, which displays increased MMP activity^158^. A collection of small-molecule BODIPY dyes, designated BODIPY-Mito-n, has been developed for fluorescence imaging applications targeting mitochondria. The addition of poly(ethylene glycol) chains to the triarylphosphonium cation moiety markedly enhanced the biocompatibility of the probes. All BODIPY-Mito-n compounds exhibited significant MMP-sensitive localization inside the mitochondria, with Pearson’s correlation coefficients (PCC) ranging from 0.76 to 0.96. Compounds BODIPY-Mito-2 and BODIPY-Mito-6 exhibited the greatest sensitivity to the MMP, with emission intensity reductions of 62% and 75%, respectively, after MMP depolarization. This increased sensitivity to MMP, as well as the higher biocompatibility of BODIPY-Mito-6, could be beneficial in the development of new probes for mitochondrial imaging in the future. The findings of the study demonstrate that various parameters must be taken into account in the development of probes that are targeted to the mitochondria, with increased sensitivity to MMP, and BODIPY-Mito-n analogs could be effective in detecting alterations in mitochondrial functions associated with cancer and cardiovascular diseases^159^.

Besides enzymatic activation, pH-sensitive off-on type probes have been of great interest because of their potential to take advantage of the acidic microenvironments seen in tumors, lysosomal compartments, or infection sites. These responsive probes commonly contain moieties, such as dimethylaminophenyl, morpholine, or pyridine groups, which experience conformational or electronic changes triggered by protonation in acidic environments, leading to fluorescence increase^160^. For example, pH-activatable probes responsive in the pH range of 4.5 to 6.5 are ideally suited for the imaging of endosomes and lysosomes, in addition to the visualization of the acidic spaces in tumors^161^.

**Fig. 13:**
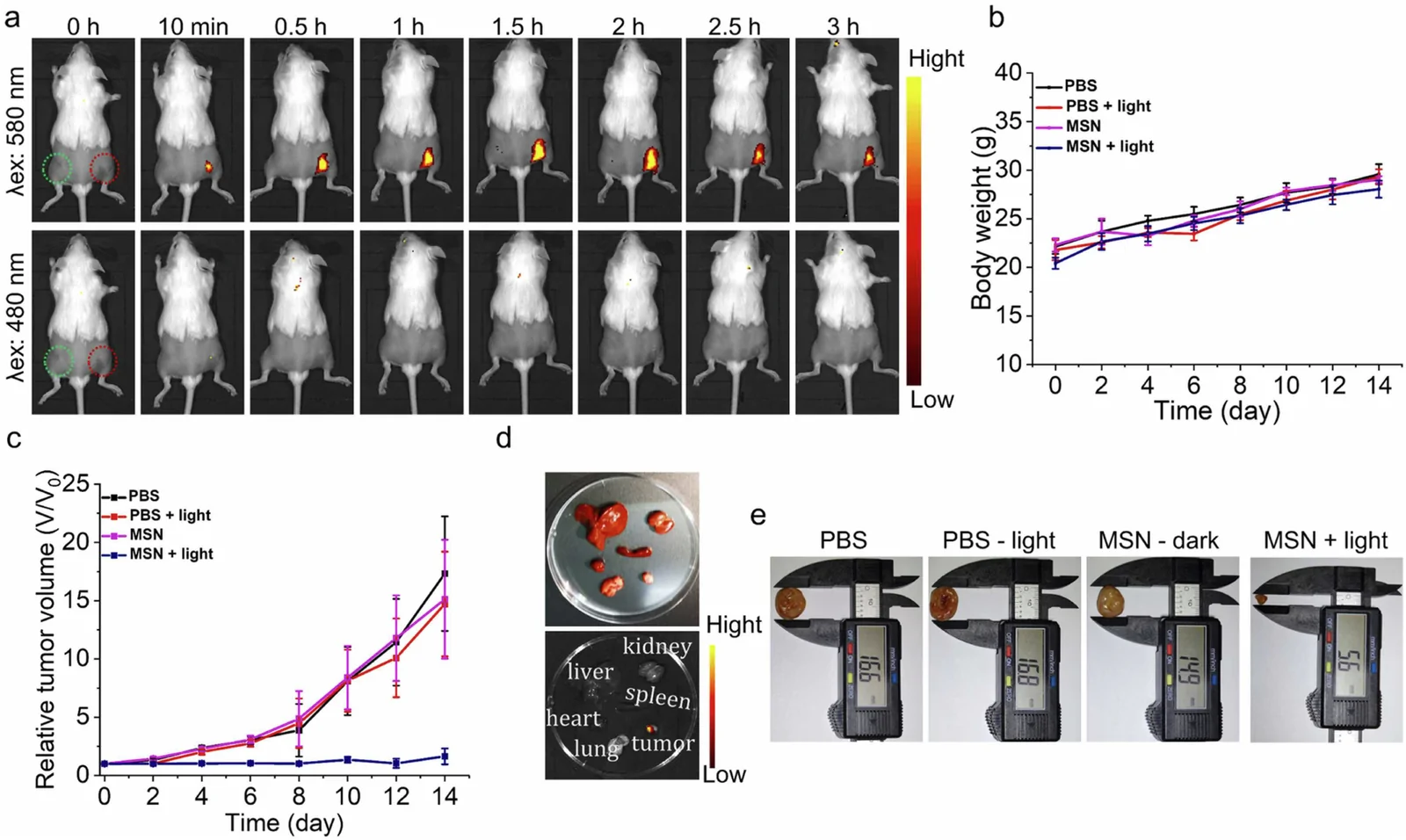
Fluorescence imaging and phototherapeutic studies in vivo with probe MSN. **a** Fluorescence images of tumor tissues (red circle) and normal tissues (green circle) recorded in two channels after MSN injection at different time points: λ_ex = 480 nm/λ_em = 620 nm and λ_ex = 580 nm/λ_em = 670 nm. **b** Body-weight changes of mice from different treatment groups monitored over 14 days. **c** Relative tumor-volume changes in mice from different treatment groups over 14 days. **d** White-light and fluorescence images of major organs, including heart, liver, spleen, lung, and kidney, together with tumor tissues. **e** Representative tumor images collected from mice after 14 days of different treatments. Reproduced with permission^163^.

Lysosome-targeting probes have emerged as a focal point in the field of anticancer therapies. Ulrich et al. described several aniline- and iodine-substituted derivatives of BODIPY, which were presented as potential lysosome-targeting and pH-activated theranostic agents for photodynamic therapy, showing high light-induced in vitro cytotoxicity. Reducing collateral damage to surrounding normal tissues is important for maintaining the accuracy and effectiveness of photodynamic therapy (PDT)^162^. A pre-photosensitizer has been designed smartly to target specifically within the lysosomes of tumor cells, showing an appropriate range of pH responses (pH < 5.0) by making simple molecular adjustments, and has been successfully applied in switch-on photodynamic therapy, supported by single-crystal structure analysis, pH testing, cell uptake studies, and in vivo experiments. Fluorescence imaging with a mouse model demonstrates that the compound is present in an “off” state within normal tissue and is “on” within tumor areas. This activatable pre-photosensitizer, with specificity for targeting lysosomes, opens up new directions for intentional tumor-specific responsive photosensitizer design. Hence, a pre-photosensitizer against a lysosome-pH target for a cancer cell, MSN, has been successfully designed by adjusting its substituent group (introducing -CN). In addition, MSN’s range of pH responses (pH < 5.0) has been tested using an in vitro approach, supported by single-crystal structures of MSN and MSNH⁺. Furthermore, favorable cell uptake and singlet oxygen generation ability of MSN, a characteristic, enable autonomous activation and transformation into MSNH+ within the lysosomal microenvironments of different types of cancer cells, including HepG2, HeLa, and 4T1 (Fig. 13). Preparation of MSN is a novel approach for controlled development of a tumor-specific, activatable, and safe photosensitizer for effective cancer therapy^163^.

**Fig. 14:**
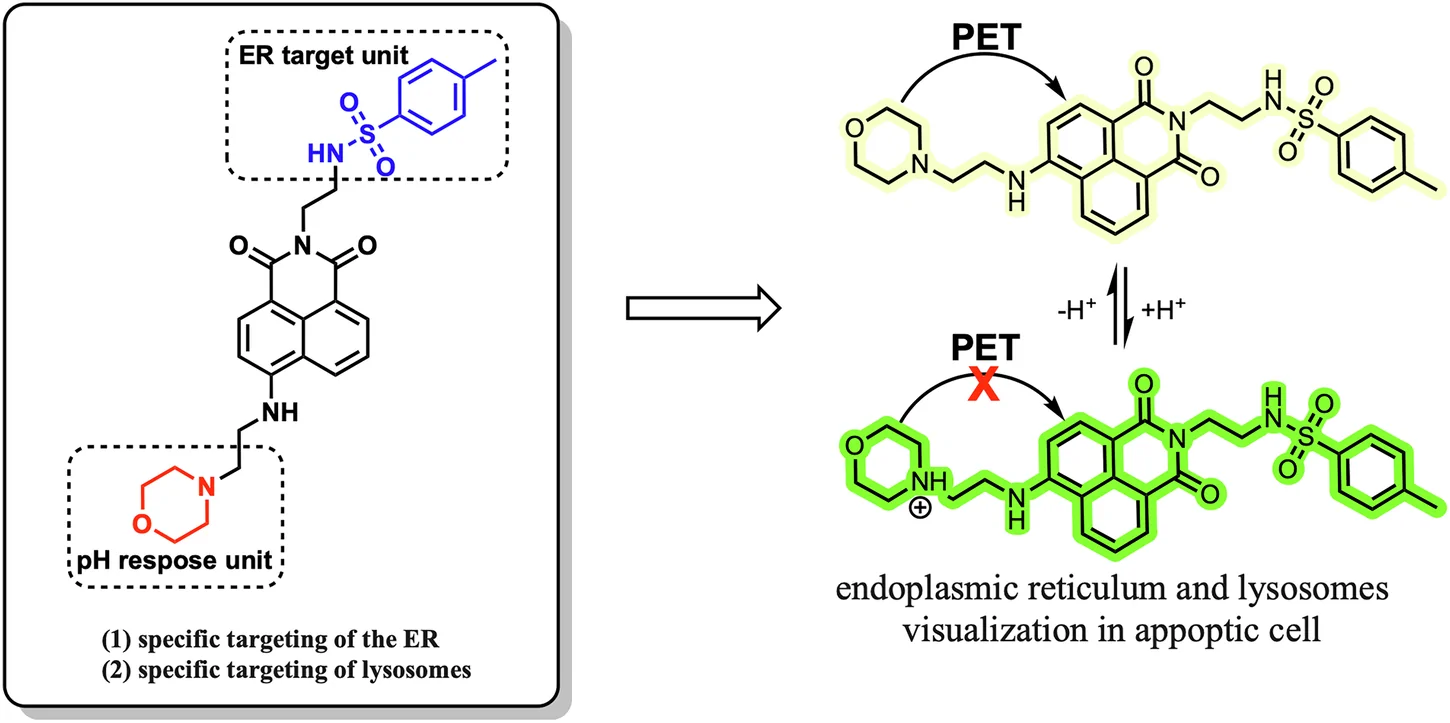
Designing strategy of a single probe (EL) based on naphthalimide for ER and lysosomes detection and imaging. Schematic illustration of discriminative visualization of endoplasmic reticulum and lysosomes in apoptosis and autophagy using a single fluorescent smart probe.

Yang et al. successfully designed a novel fluorescent probe for monitoring apoptosis and autophagy by simultaneously visualizing the endoplasmic reticulum (ER) and lysosomes. The probe EL showed a prominent change in fluorescence intensity with reference to varying pH environments, promoting discrimination of ER and lysosomes based on their distinct pH profiles. The probe efficiently evaluated apoptosis by a decline in intensity while also reflecting an enhancement of intensity during autophagy and hence created a distinct discrimination of apoptosis and autophagy. The potential applications of EL for visualizing ER and lysosomes, along with monitoring dynamic variations during physiology, are vast, and hence contribute significantly towards an enhanced understanding of the physiological and pathological significance of such organelles. This work is of significant value for the preparation of multifunctional probes for targeting other organelles with potential towards monitoring dynamic variations of subcellular compartments during various processes. Follow-up studies are needed with an objective of developing multifunctional probes for monitoring ER, lysosomes, and a wide range of analytes simultaneously, which would further pinpoint the functional activities of ER, lysosomes, and other analytes within respective organelles within different physiology and pathophysiological scenarios (Fig. 14)^164^.

**Fig. 15:**
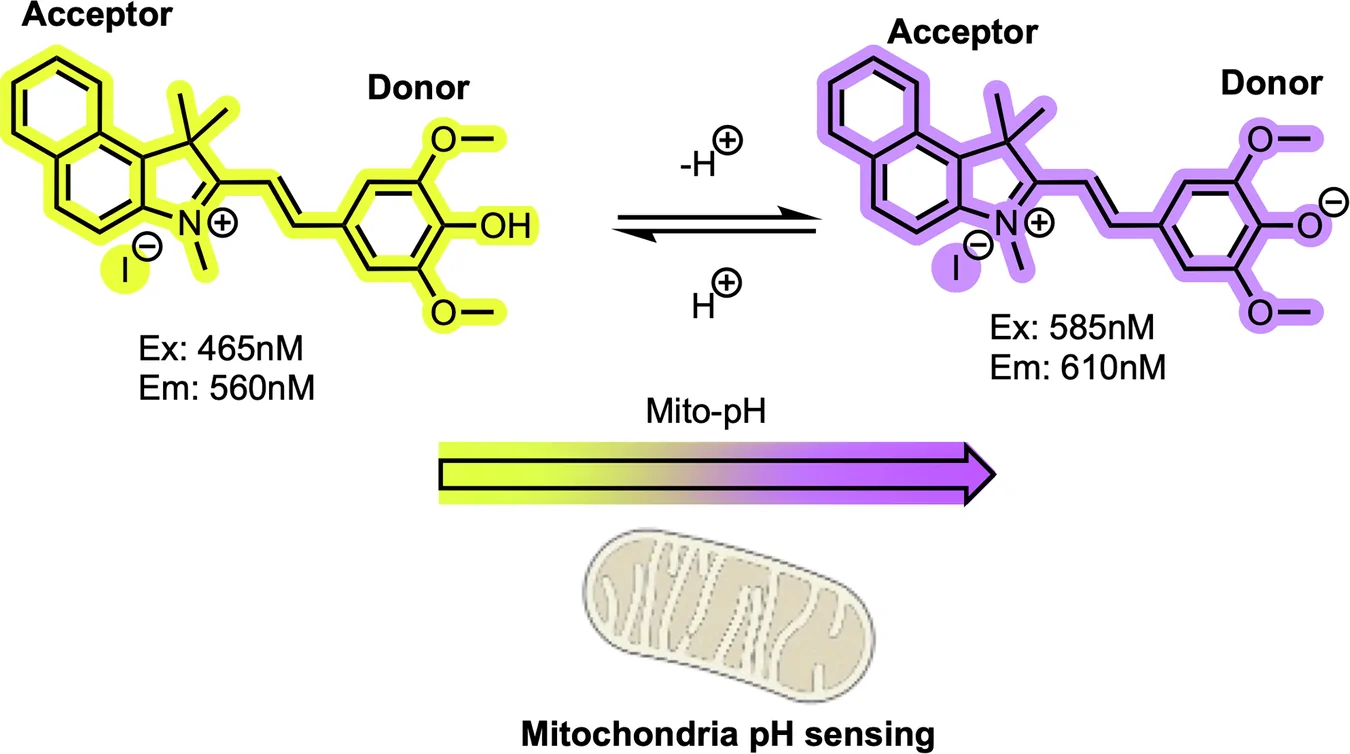
Proposed response mechanism of Mito-pH2 towards pH. Schematic illustration of red-shifted donor–acceptor hemicyanine-based probe for mitochondrial pH in live cells.

The ability to sense acidity has been utilized for targeted administration and visualization of chemotherapeutic agents. In these applications, initially the probe is non-fluorescent while circulating in the systemic environment but turned into a strongly fluorescent when it comes to contact with the acidic microenvironment of malignant tissue, validating the accumulation of the drug and enabling targeted therapy^23,165^. The pH level is a critical regulator of vast numbers of cellular processes and activities. The inner mitochondrial membrane (IMM) is distinguished by nanometer-scale pH gradients derived from the electron transport chain (ETC) for mitochondrial membrane potential (MMP) establishment and subsequent ATP generation and thermogenesis. Tools for partitioning of pH gradients from total IMM potential within living bodies are relatively scarce. In trying out conjugating a fluorescent benzo-indole chromophore with a phenol-sensitive site into a compact covalent structure, a sensitive, red-shifted, cell-permeant pH indicator (Mito-pH2) is reported. Mito-pH2 is specifically mitochondria-specific for localization by IMM being a putative target site by virtue of amphiphilic cationic character and is capable of sensing dynamic change in pH within mitochondria of living bodies. Constructing Mito-pH2 is done with a strategy of incorporation into a doubly exciting and doubly emitting peak for sensing by a ratiometric measurement of pH. Mito-pH2 is shown to be able to expand over a range of 3–9 with a characteristic color change from yellow to purple, making it a sensitive and versatile colorimetric probe for sensing of pH. Density functional theory (DFT) computations and spectroscopy are used for revelation of putative sensing mechanisms, including description of photophysics. Quantitative live-cell fluorescence microimaging measures the dynamic change in mitochondrial pH with extracellular change in pH is sensed by Mito-pH2 with minimal, if any, detectable cytotoxicity with subsequent live microimaging. Red-emitting Mito-pH2 allows quantitative mapping of physiological mitochondrial membrane pH and extends the range of mitochondria-penetrating, low-toxicity probes for sensing environmental conditions with mitochondrial state (Fig. 15)^166^.

**Fig. 16:**
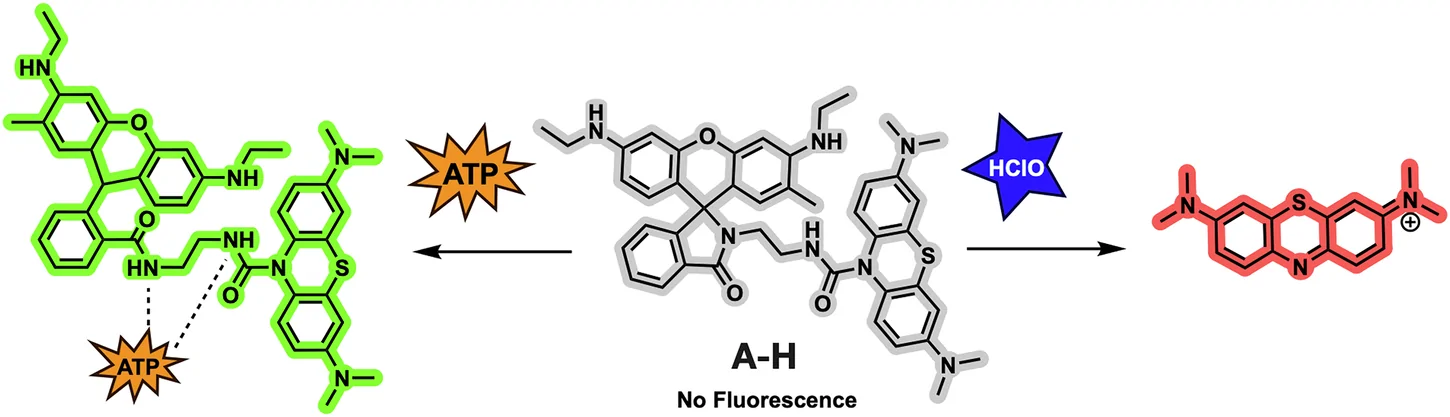
Design strategy and responsiveness of fluorescent probe A-H. Schematic representation fluorescent probe A-H, for distinguishing HClO and ATP in normal and cancer cells and useful for NIR PDT.

A new fluorescent probe, A-H, has also been developed by Liu et al. to improve cancer diagnosis and therapeutic efficiency, having a specific response to adenosine triphosphate (ATP) and hypochlorous acid (HClO). This is the first fluorescent probe combination for ferroptosis and near-infrared Photodynamic therapy (PDT), enhancing therapeutic efficacy. The probe’s ability to differentiate between normal and cancerous cells results in improved diagnostic accuracy. In addition, upon reaction with the overexpressed HClO, it triggers the release of methylene blue, which is utilized for photodynamic therapy PDT. This strategy successfully combines imaging and therapeutic techniques for cancer diagnosis, thus improving the effectiveness of treatment and overcoming the limitations of single cancer treatment techniques (Fig. 16)^167^.

**Fig. 17:**
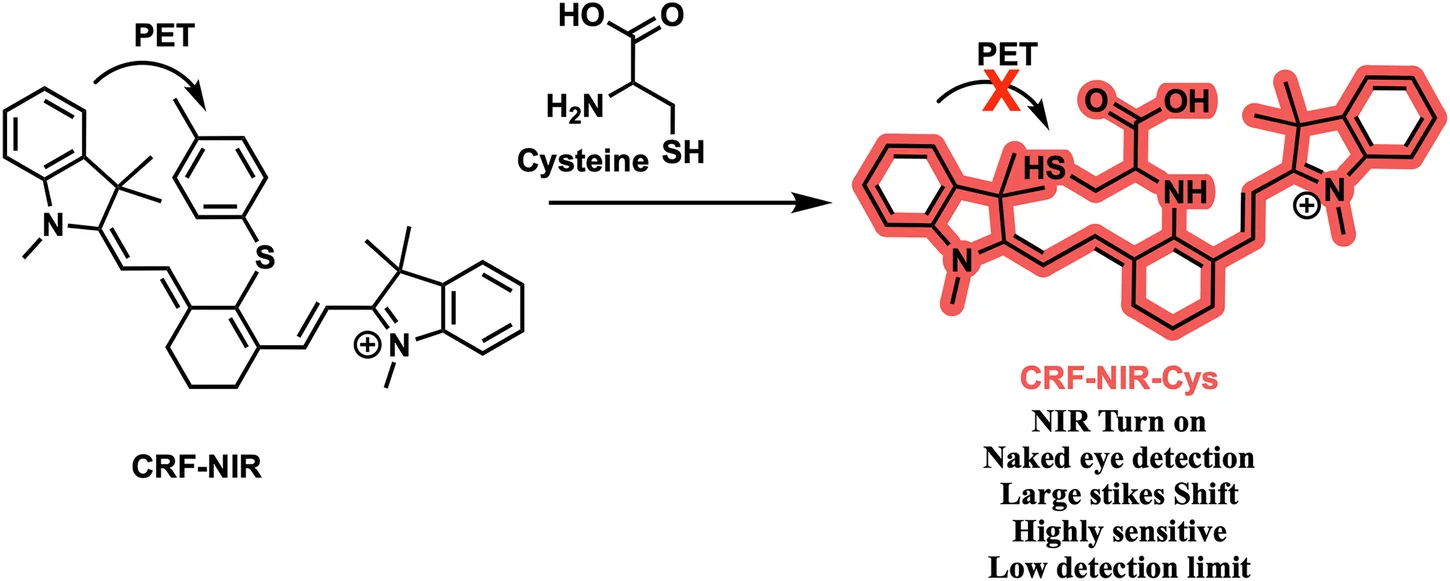
A NIR cyanine-based probe CRF-NIR for selective detection of Cys. Cys in mitochondria detection via cyanine NIR fluorescent probe CRF-NIR.

**Fig. 18:**
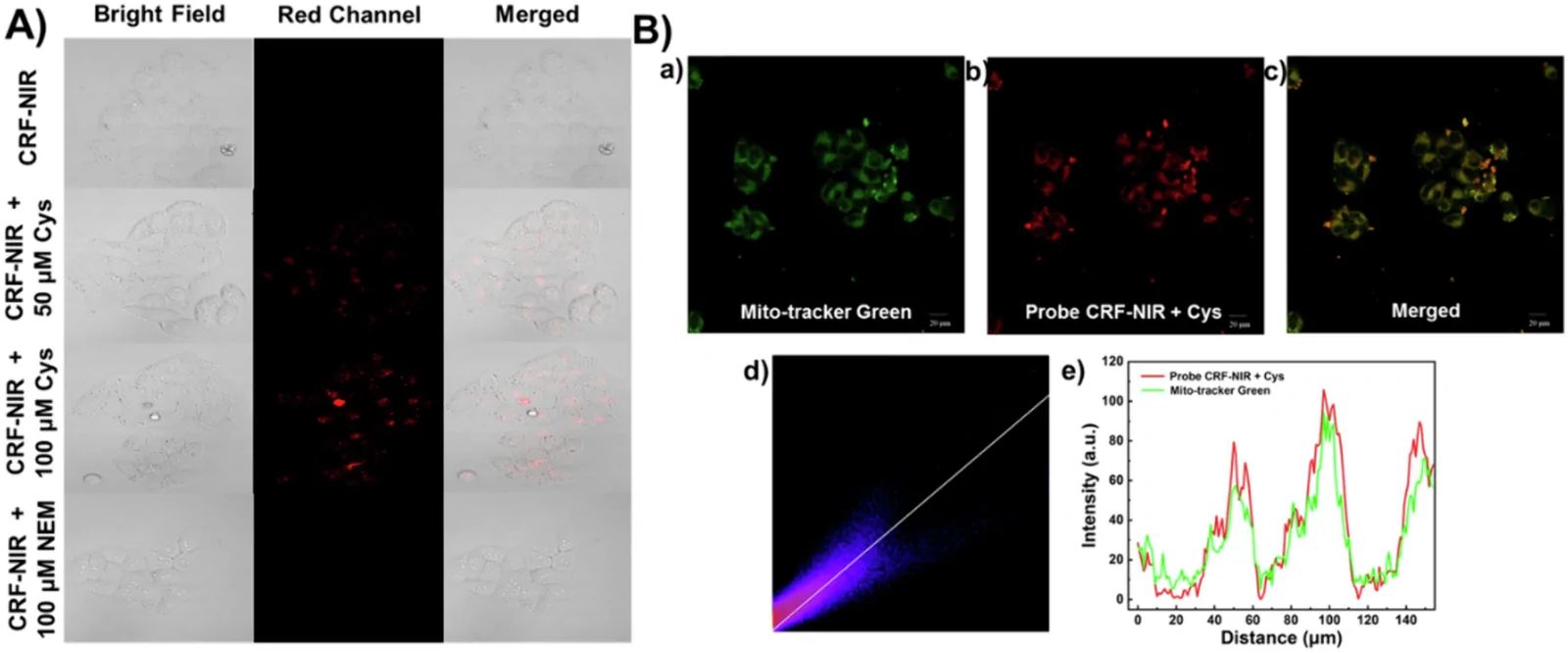
Co-localization experiment of CRF-NIR in HeLa cells. **A** Confocal fluorescence imaging using the probe CRF-NIR (20 μM) in HeLa cells treated with Cys (50 μM and 100 μM) and NEM (100 μM). Images were acquired in the red channel (700–800 nm) with excitation at 635 nm. **B** Co-localization experiment of CRF-NIR in HeLa cells: (a) confocal laser scanning microscopy (CLSM) image of Mito-Tracker Green (MTG) in the green channel; (b) CLSM image of CRF-NIR (20 μM) after the addition of Cys (100 μM) in the red channel; (c) overlay of (a) and (b); (d) fluorescence intensity correlation plot of MTG and CRF-NIR + Cys in the two fluorescence channels; and (e) intensity profiles of the green and red channels. a Confocal laser scanning microscopy (CLSM) image of Mito-Tracker Green (MTG) in the green channel; b CLSM imaging of CRF-NIR (20 μM) after the addition of Cys (100 μM) in the red channel; c Overlay of (a, b). Reproduced with permission^168^.

Recently developed fluorescent probe, CRF-NIR, was designed for specific detection of Cysteine (Cys) within mitochondria. The Cyanine NIR probe showed a near-infrared emission at 752 nm, with a significant Stokes shift upon cysteine presence. Besides mitochondrial targeting capability, the effectiveness of the probe was also confirmed through a low detection limit of 0.22 μM. The effectiveness of the probe in mitochondrial targeting is expected to offer insights into physiological processes as well as the diagnosis of associated physiological disorders. The results obtained from the fluorescence colocalization studies also offered evidence for the effectiveness of the probe in mitochondrial targeting, along with its superior imaging capability for the detection of cysteine (Cys) (Figs. 17, 18)^168^.

**Fig. 19:**
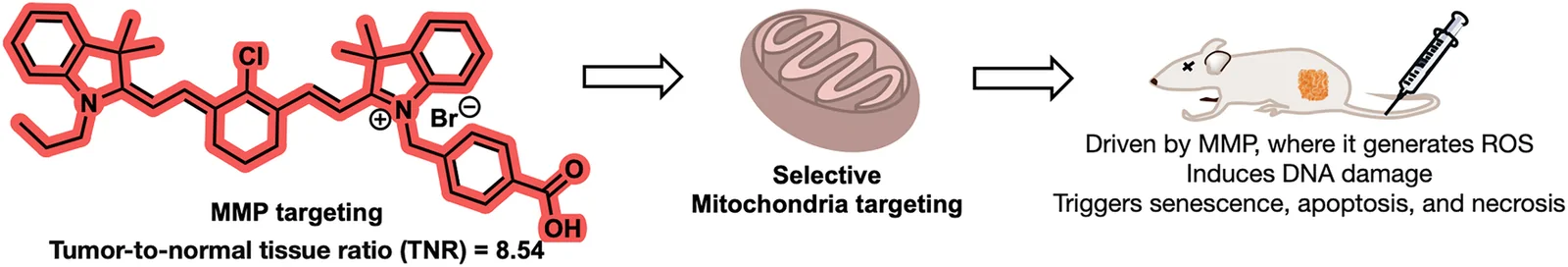
NIR photosensitizers based on heptamethine core with in vivo imaging capabilities. Mitochondria-targeted heptamethine cyanine-based imaging agent tumor-targeting abilities.

**Fig. 20:**
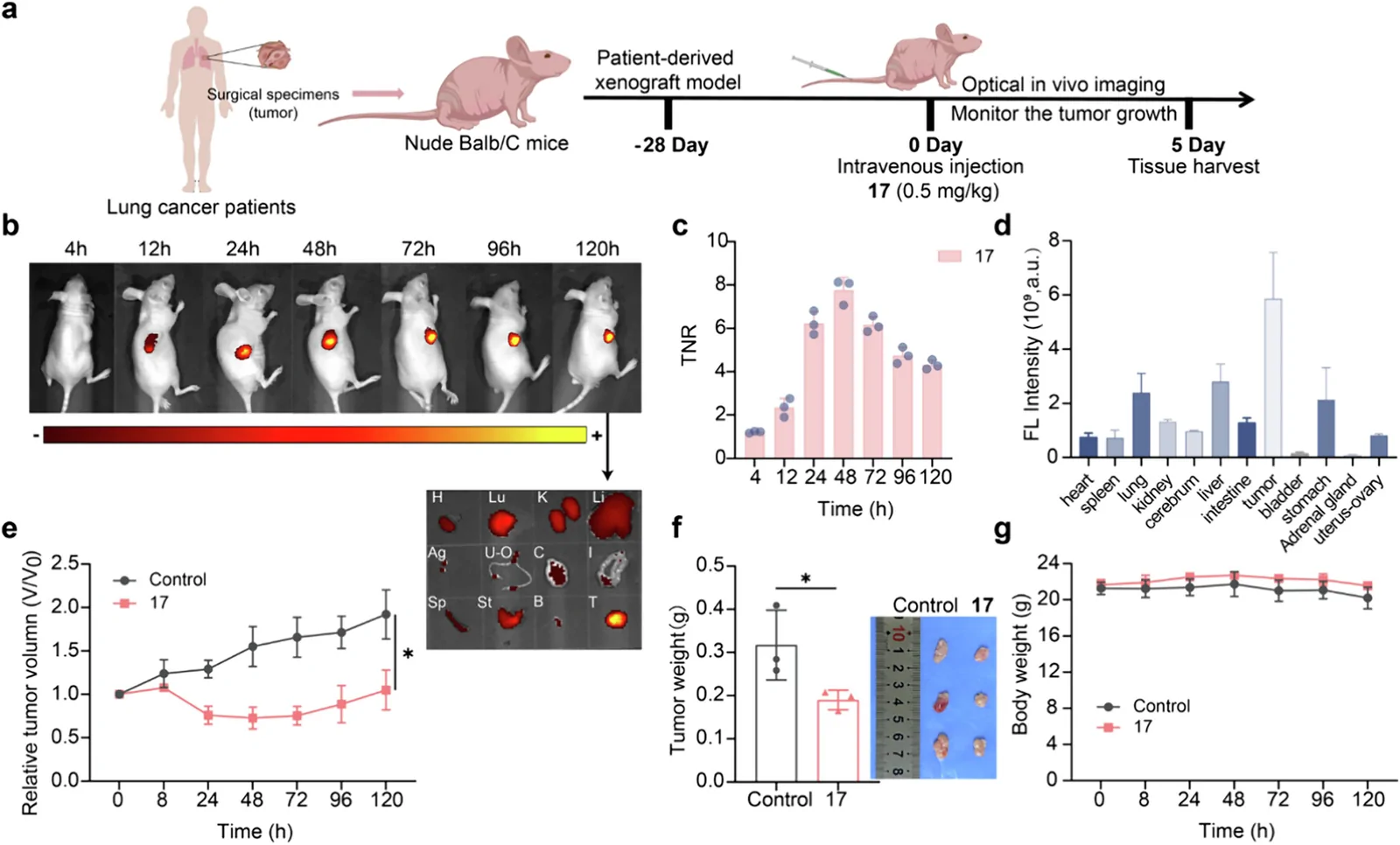
Targeted imaging and therapy in a patient-derived xenograft model using compound 17. **a** Schematic illustration of the clinical application study. **b** In vivo fluorescence signal distribution in mice at different time points after intravenous administration of compound 17 at 0.5 mg/kg, including ex vivo fluorescence imaging of major organs and tumors collected 120 h post-injection. **c** Quantitative analysis of the tumor-to-normal tissue fluorescence ratio (TNR). **d** Ex vivo biodistribution analysis of compound 17 in Balb/c nude mice at 120 h post-injection. **e** Tumor-growth curves of mice treated with PBS as the control or compound 17 at 0.5 mg/kg. **f** Tumor weights and representative tumor images from each treatment group on day 5. Each data point represents the result obtained from one tumor treated with compound 17. **g** Body-weight changes of mice in different treatment groups over time. Data were expressed as mean ± SD, unpaired two-tailed Student’s *t* test. *p < 0.05 (*n* = 3). Reproduced with permission^169^.

A more recent detailed study on cyanine dye-based NIR probes derived a series of heptamethine cyanine photosensitizers with symmetric and asymmetric architectures (Fig. 19). The novel NIR probes were synthesized and evaluated, and among them, a heptamethine cyanine fluorescent compound with an asymmetric architecture and specific mitochondrial targeting showed improved benefits of near-infrared fluorescence (NIRF) imaging, tumor specificity, and significant anticancer activity. Its high mitochondrial accumulation is supported by MMP, which is further responsible for increasing reactive oxygen species (ROS) generation. This generation further causes damage to DNA, senescence, apoptosis, and necrosis. Its high therapeutic activity was verified by multiple experimental models, including patient-derived xenograft (PDX), without any toxic impact. Compound 17 is a potential radiopharmaceutical drug for personalized medicine, with single-dose activity, long-term tumor retention, and reduced off-target accumulation (Fig. 20)^169^.

**Fig. 21:**
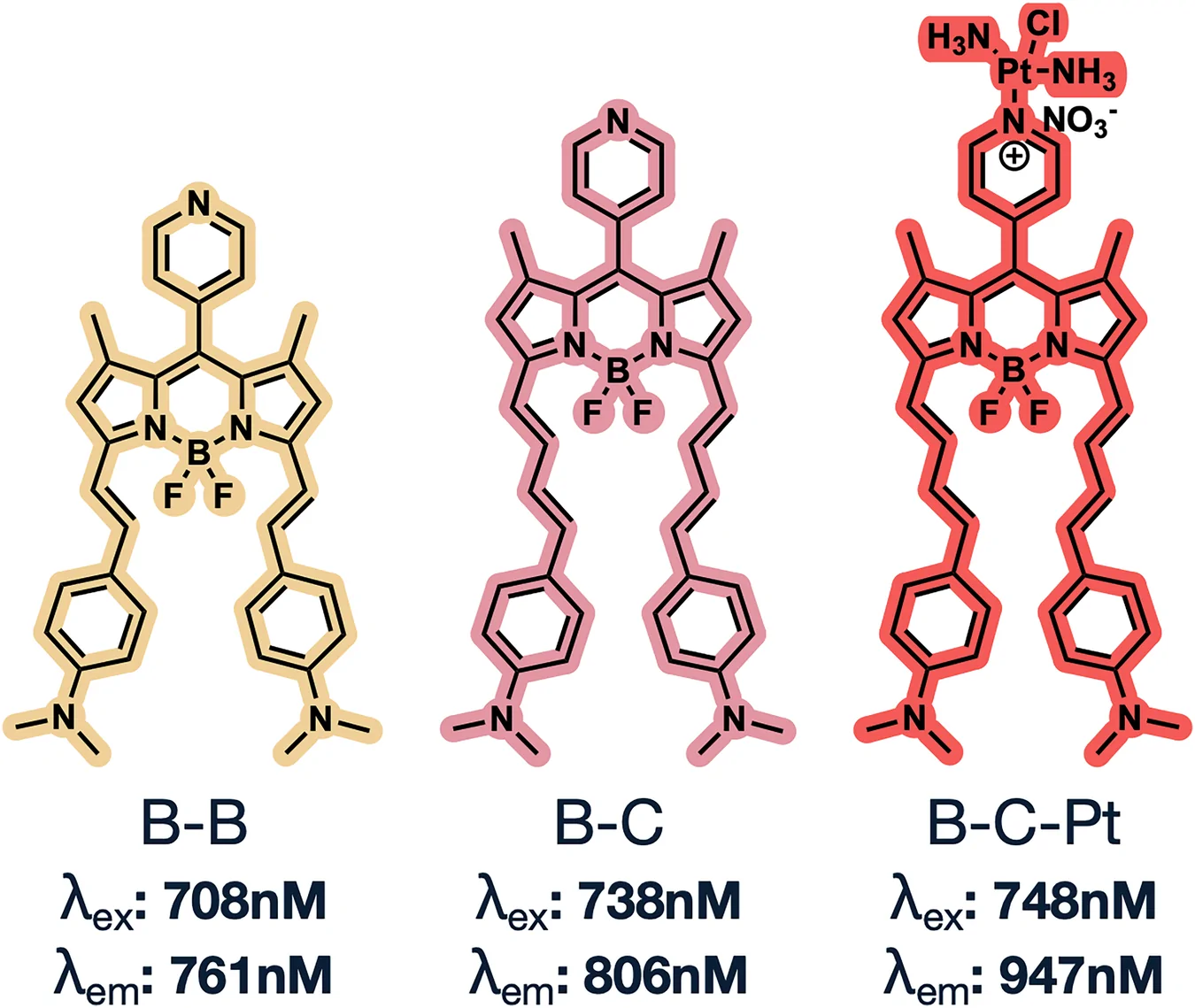
D-π-A-structured near infrared II fluorescent BODIPY probes. BODIPY based near Infrared II Fluorescent probes (B-B, B-C, and B-C-Pt) towards photoacoustic imaging-guided synergistic phototherapy and chemotherapy of cancer.

These polymethine-derived fluorescent dyes present modifiable emission features, high extinction coefficients, and functionalizability, hence making them suitable for conjugation with target ligands, peptides, or antibodies^170,171^. Therefore, cyanine dyes have been widely applied in many molecular imaging applications, ranging from cancer diagnostics and lymph node mapping to real-time visualization under surgical procedures. Towards improving quantum yield and photostability, structural alterations, like the introduction of rigidified backbones or side-chain zwitterions have been explored. BODIPY analogs have also attracted great interest as potential future-generation NIR fluorophores^172^. BODIPY-derived fluorescent dyes are especially beneficial due to their unique peaks of emission, high quantum yields, and excellent chemical stability^173^. Through the use of structural engineering methodologies like π-extension or introducing electron-donating or withdrawing groups, BODIPY analogs have been tailored with longer-wavelength emissions, hence making them suitable for use in applications requiring deep-tissue imaging. Their small size and versatility further make them suitable as scaffolds for activatable or environment-sensitive probes. Xing et al. discovered a BODIP-based NIR Fluorescent probe for cancer therapy. The BODIPYs showed photothermal conversion capability with strong fluorescence in the NIR. D-π-A-structured near infrared II fluorescent BODIPY probes, named B-B, B-C, and B-C-Pt, were designed and synthesized for photoacoustic imaging-guided synergistic phototherapy and chemotherapy of cancer. The cisplatin modified BODIPY probe (B-C-Pt) showed an excellent capability for singlet oxygen generation, and remarkable features for near-infrared absorption and fluorescence. When encapsulated with distearoyl phosphoethanolamine polyethyleneglycol 2000 (DSPE-PEG-2000), B-C-Pt showed red shifting absorption and fluorescence, which corresponded with wavelengths of 762 and 985 nm, respectively. The quantum yield of singlet oxygen (^1^O_2_) and photothermal conversion efficiency of B-C-Pt were determined to be 4.0% and 40.6%, respectively. In addition, B-C-Pt nanoparticles showed remarkable chemotherapeutic activity, with an IC_50_ value of 12.3 µmol/L. In vivo and in vitro studies supported that B-C-Pt are excellent multifunctional probes for photo-chemotherapy, which combine phototherapy with chemotherapy for cancer treatment (Fig. 21)^174^.

**Fig. 22:**
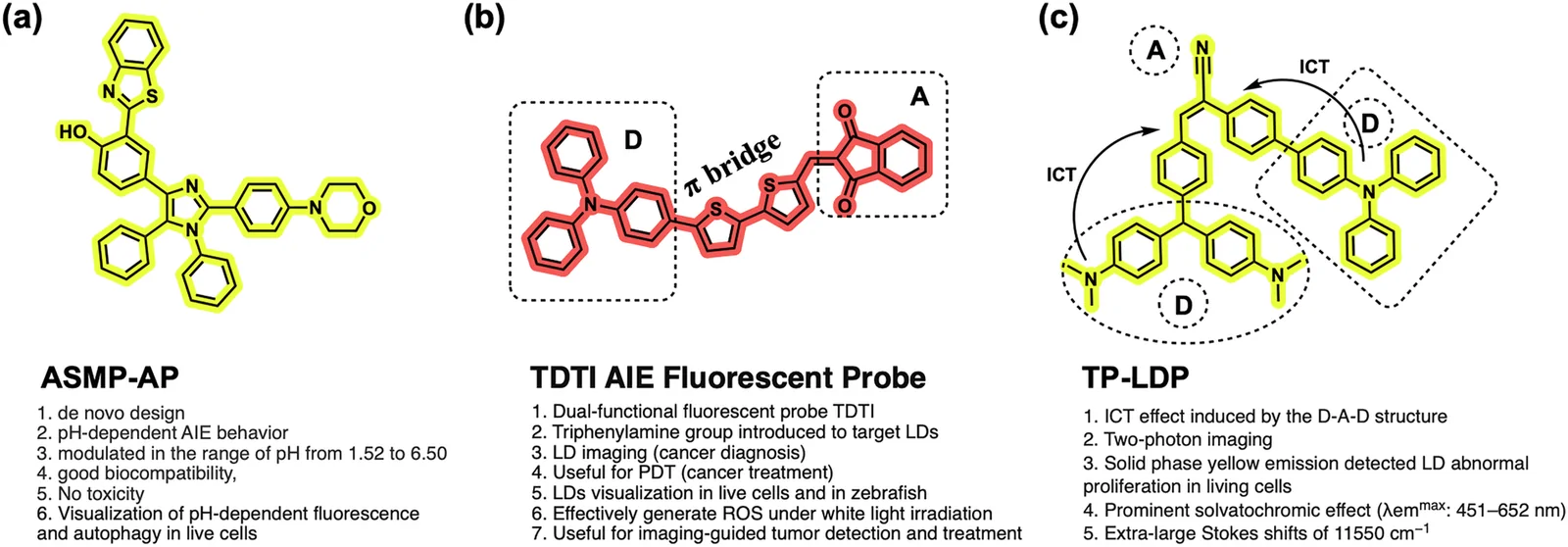
Representative example of AIE fluorescent probes for imaging and sensing application. **a** De novo design of a novel pH-sensitive AIE probe, ASMP-AP, towards lysosome targeting to facilitate the analysis of autophagy. **b** Schematic representation of novel dual-functional fluorescent probe TDTI with AIE feature LD imaging (cancer diagnosis) and PDT (cancer treatment). **c** LD-targeting fluorescent probe (TP-LDP) with AIE feature for LD imaging.

Additionally, the physicochemical characteristics of AIE molecules can be easily adjusted for the synthesis of responsive probes for the detection of a variety of biological responses to different stimuli, such as pH changes, enzyme reactions, redox reactions, as well as changes in ionic concentrations. Probes can also be created with the capabilities of being non-fluorescent while in systemic circulation and fluorescent only at targeted sites, e.g., acidic microenvironments within tumors, sites of infection or inflammation, or sites of infection. The acidic tumor microenvironment triggers macro-autophagy as an adaptive and defensive mechanism which is an important self-protection mechanism of cells, and its failure causes diverse diseases. As a result, autophagy is responsible for keeping cancer cells alive during acidic conditions and contributing towards drug resistance, making it an attractive target for drug intervention against acidic, hypoxic tumor microenvironments^175^. Fluorescent probes with specific targeting of lysosomes are essential to evaluate the complex dynamics of autophagy. ASMP-AP, a small-molecule probe with precise targeting of lysosomes and a high sensitivity to pH variation, enables the monitoring of autophagy processes in zebrafish, thus opening possibilities for in vivo investigations of autophagy (Fig. 22a)^176^.

Enzyme activity monitoring is an effective method in the process of disease diagnosis. In acidic environments and nutrient deficiency, cancer cells accumulate excess lipids into lipid droplets for future use. These excess lipids possess the ability to improve β-oxidation during states of energy distress and counteract lipotoxicity. Zhang’s group designed a AIE fluorescent probe fluorescent probe for lipid droplets (TDTI), facilitating simultaneous monitoring of lipid droplets and imaging-guided PDT for cancer diagnosis and treatment. The probe can selectively target lipid droplets with the ability to provide precise dynamic observation, specific imaging, and cancer cell differentiation. The probe shows efficiency in the production of reactive oxygen species and has been found to significantly inhibit the proliferation and induce apoptosis of HeLa cells. This shows the potential of this probe, which is capable of performing two functions, to be used in the diagnosis and treatment of diseases related to cancer (Fig. 22b)^177^. Some of the AIE systems have also been found to have the ability to perform two-photon fluorescence, which helps to achieve high-resolution imaging of densely scattering biological tissue or the entire organ using minimal photodamage^178^. These advances have significantly increased the potential of using the AIE probes, which have moved from the imaging of cells to the imaging of the whole animal, which may be used to perform clinical diagnoses and surgeries. Hao group from China developed AIE based Two Photon-LDP bioprobe to visualize the abnormality of the lipid droplet (LD) and was expected to overcome the limitations of the existing commercially available LD probes. Photostability, two-photon stimulated fluorescence imaging capability, and lipid droplet selectivity were explored using experiments within cellular contexts, and it is suggested that it could be useful as a diagnostic tool for LD-related disease (Fig. 22c)^179^. This selective activation minimizes off-target fluorescence and maximizes signal-to-noise, further enhancing the resolution and diagnostic accuracy of imaging methods. For example, pH-sensitive AIE probes with pH-sensitive linkers can be triggered in acidic microenvironments, such as endosomes and lysosomes, that are normally traversed during cell internalization of drug-loaded nanoparticles. Such pH-responsive probes can make accurate tracking of trafficking of nanoparticles inside cells possible and can serve as markers of successful release from endosomes or payloads. Use of AIE probes is especially beneficial for targeting tumors, where localized aggregation often occurs owing to the unique pathophysiological characteristics of tumors. Solid tumors have an increased permeability and retention effect, in addition to characteristics such as irregular blood vessels, increased interstitial pressure, and poor lymphatic drainage—conditions favorable for nanoscale drug delivery systems to accumulate and cluster^180^. When delivered to the target location in the tumor, AIEgens carried by these systems produce an intense fluorescent signal, which is linearly dependent upon the local drug concentration. This advantage not only aids in the visualization of drug accumulation in the tumor but also helps in measuring the targeting efficacy. Some of the AIE probes have also been developed to further aggregate upon interaction with tumor-diagnostic biomarkers or enzymes, which helps to achieve enhanced fluorescence and imaging with high contrast. This is particularly useful to achieve the precise delineation of the tumor margin, metastatic spread, or drug delivery^181^.

**Fig. 23:**
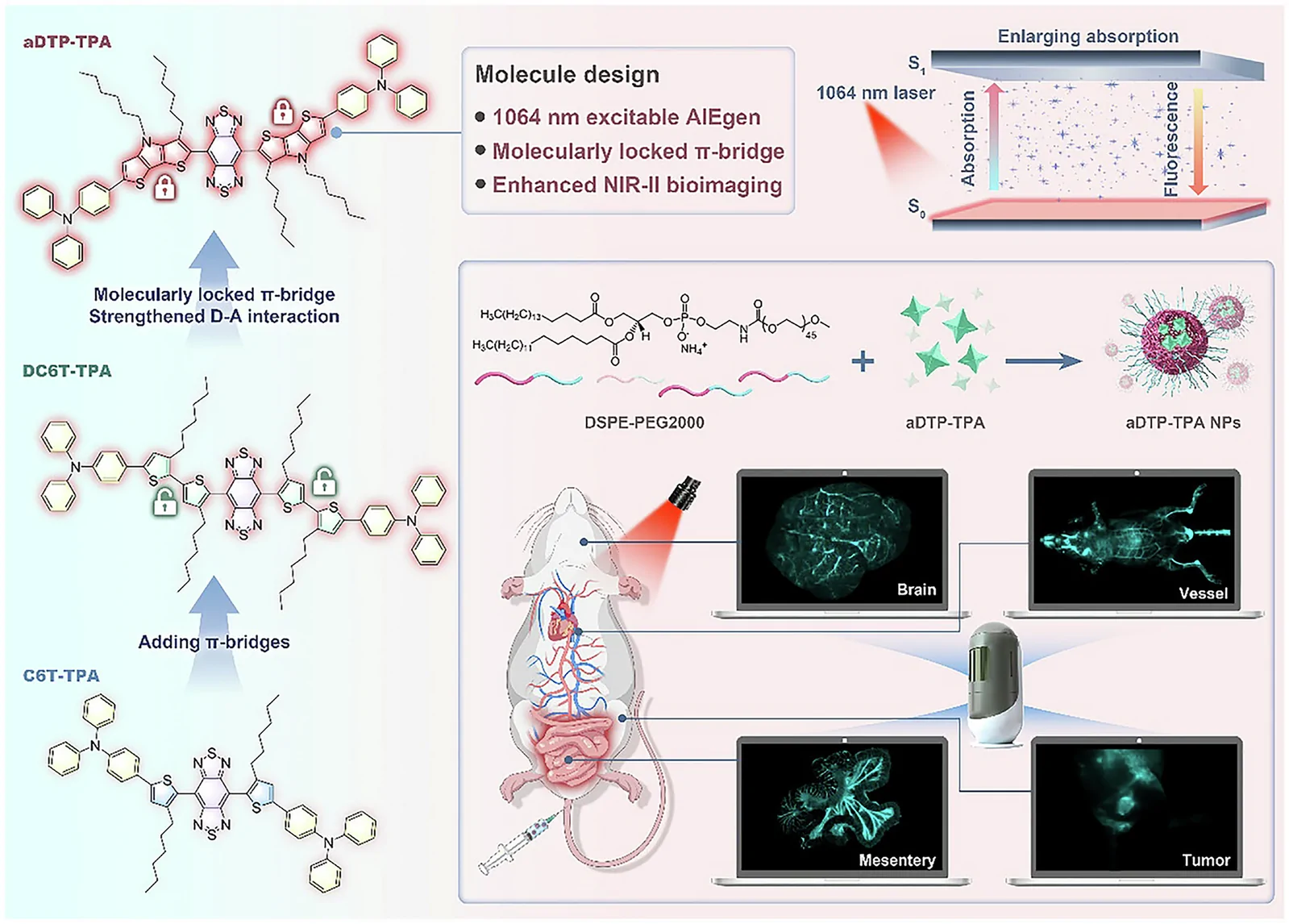
Molecular lock design based on alkyl-substituted dithieno[3,2-b:2′,3′-d]pyrrole (aDTP)NIR II probes for biomedical applications. NIR-II emission under 1064 nm excitation, aDTP-TPA nanoparticles (NPs) demonstrate exceptional performance as fluorescent agents for deep tissue imaging, angiography, and NIR-II-guided tumor resection. Reproduced with permission^182^.

aDTP-TPA nanoparticles was developed by Liu et al., representing an innovative AIE nanoparticle system that can be excited by NIR-II light based on the design of molecularly locked π-bridge architecture. Specifically, the π-bridge architecture is designed to enhance π-conjugation and donor-acceptor interaction without aggregation-caused fluorescence quenching, leading to excellent excitation capability in the NIR-II window and high-resolution imaging within tissues. Remarkably, the cRGD-conjugated aDTP-TPA nanoparticles were able to accumulate selectively within tumor sites and accurately define tumor borders using NIR-II imaging, thus facilitating the successful surgical resection of 4T1 tumors (Fig. 23)^182^.

**Fig. 24:**
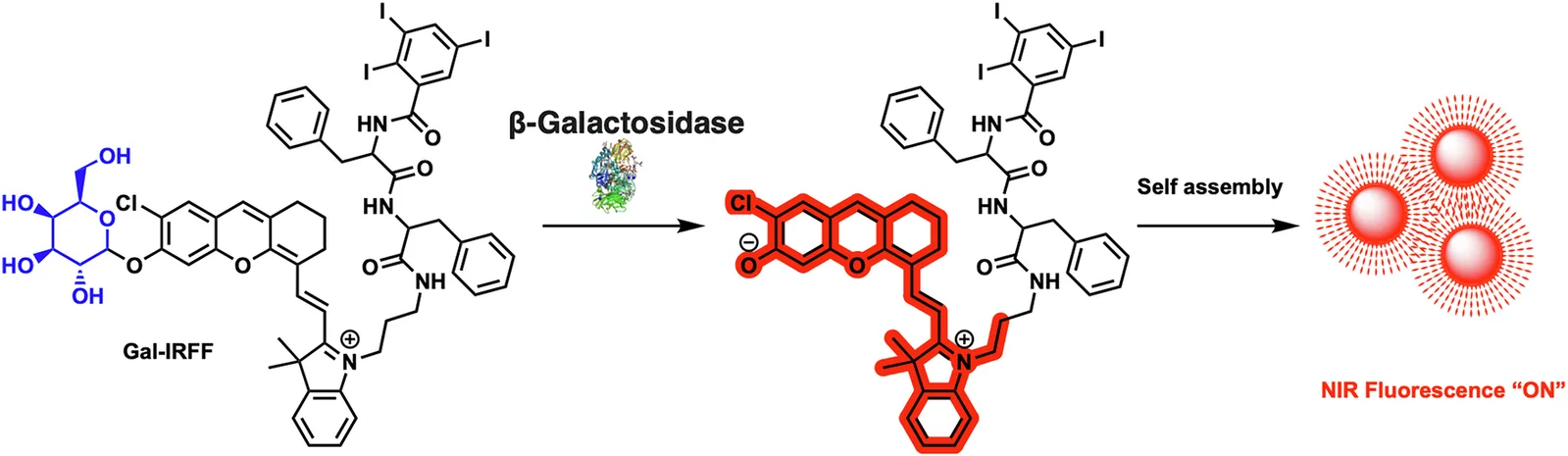
β-gal induced in situ self-assembly of Gal-IRFF. Schematic representation of the β-gal activable in situ self-assembly NIR fluorogenic imaging probe for in vivo monitoring of ovarian tumor.

Additionally, combined with diagnostic functionality, these nanoparticles are increasingly being coupled with drug delivery strategies. Fluorescent nanoparticles that are activated upon reaching the target location offer a theranostic platform that includes both therapeutic and diagnostic capabilities. QDs loaded with doxorubicin, for instance, or mesoporous silica release their payload within the acidic tumor environment while simultaneously emitting a fluorescence signal to verify the accuracy of targeted delivery.^183,184^. Currently, the treatment of Extramedullary multiple myeloma (EMM) is mainly based on chemotherapy; however, drug resistance often causes repeated therapeutic failures. Doxorubicin (DOX) induces apoptosis in cancer cells by disrupting DNA repair mechanisms and stimulating the formation of reactive oxygen species. However, DOX is non-selective and has significant side effects, including cardiotoxicity and myelosuppression. Research has shown that nanoscale CXCR_4_-PEG-CdTe-DOX increases the drug concentration within specifically targeted multiple myeloma cells and prevents toxic side effects. Polyethylene glycol (PEG) is hydrophilic, biocompatible, safe, non-toxic, and less immunogenic, which makes it an effective and safe targeting vector. In addition, polyethylene glycol allows various pharmaceutical delivery systems, enabling controlled release, extended duration of action, and greater cellular incorporation^183^. Despite their promise, challenges remain, including potential toxicity (especially with heavy-metal-containing QDs), photobleaching, off-target accumulation, and scalability of synthesis. Recent efforts are addressing these issues through the development of biocompatible materials, surface passivation techniques, and green synthetic routes^185–187^. Further research devised an enzyme-instructed in situ self-assembly fluorogenic probe Gal-IRFF, a small molecule activatable NIR probe, for accurate cancer detection. The probe demonstrated improved NIR fluorescence signals and self-assembled into nanoparticles by an enzyme-instructed in situ self-assembly approach. This enhanced the probe’s dispersion and retention duration inside tumor lesions. The research underscores the efficacy of this method for accurate in vivo tumor diagnosis. Gal-IRFF shown outstanding efficacy in prolonged imaging and the sensitive identification of endogenous β-gal inside intricate physiological systems. The method may be expanded to create probes that combine NIR fluorescence with other imaging modalities (Fig. 24)^188^.

One remarkably prospective category of inorganic fluorophores for the application in the field of deep tissue imaging is silver sulfide (Ag₂S) QDs, especially NIR-II-emitting Ag₂S QDs^189–191^. These nanocrystals possess high photostability, high quantum yields, and minimal cytotoxicity, which make them an even better choice for investigations in biomedical imaging. Unlike traditional QDs made of cadmium or lead, which are quite toxic, Ag₂S QDs are made from relatively biocompatible elements and can even be synthesized with different sizes for the improvement of their optical properties. The NIR-II Ag₂S QDs support deep tissue penetration with minimal autofluorescence, enabling the precise visualization of lymphatic vessels, blood vessels, and even internal organs with higher spatial resolution. The researcher from Beijing Institute of Technology reports a new strategy for the management of diseases using image guidance based on NIR-II bioimaging modalities to facilitate image-guided tumor therapy under an 808 nm excitation. The researchers develop highly biocompatible lanthanide-doped nanoparticles (RENPs-ZnPc/RB/mPEG-FA) that can emit NIR-II luminescence for imaging but also generate singlet oxygen for anticancer photodynamic therapy. The nanoparticles effectively suppress tumor growth and eradicate them without any recurrence being observed. The method shows great potential for practical applications as multifunctional probes for both imaging and therapy^192^. The ability to co-deliver therapeutic agents or genetic materials into these nanostructures also favors theranostic use, enabling concurrent diagnosis, therapy administration, and chemotherapeutic response assessment. Further study on NIR probe: Zeng et al. designed a site-specifically modified nanobody probe that includes both ICG and Ce6 for NIR fluorescence imaging and photodynamic therapy, respectively. In this particular system, the nanobody acts as the component used to target the tumor cells to deliver the imaging and therapeutic elements to the appropriate place. This study describes nanobody optical probes having site-specific modifications through which ICG is used for tumor imaging while chlorin e6 is used for photodynamic therapy. Nanobodies offer targeting specificity towards cancer cells, while PEGylation improves tumor uptake and imaging efficiency. Nanobody optical probes can effectively visualize tumors and provide a better therapeutic effect in Hep3B tumor-bearing mice. The example shows how fluorescent probes used only for imaging can become an important tool for the development of theranostic cancer treatment by coupling fluorescence detection and ROS-based tumor destruction using light^193^.

**Fig. 25:**
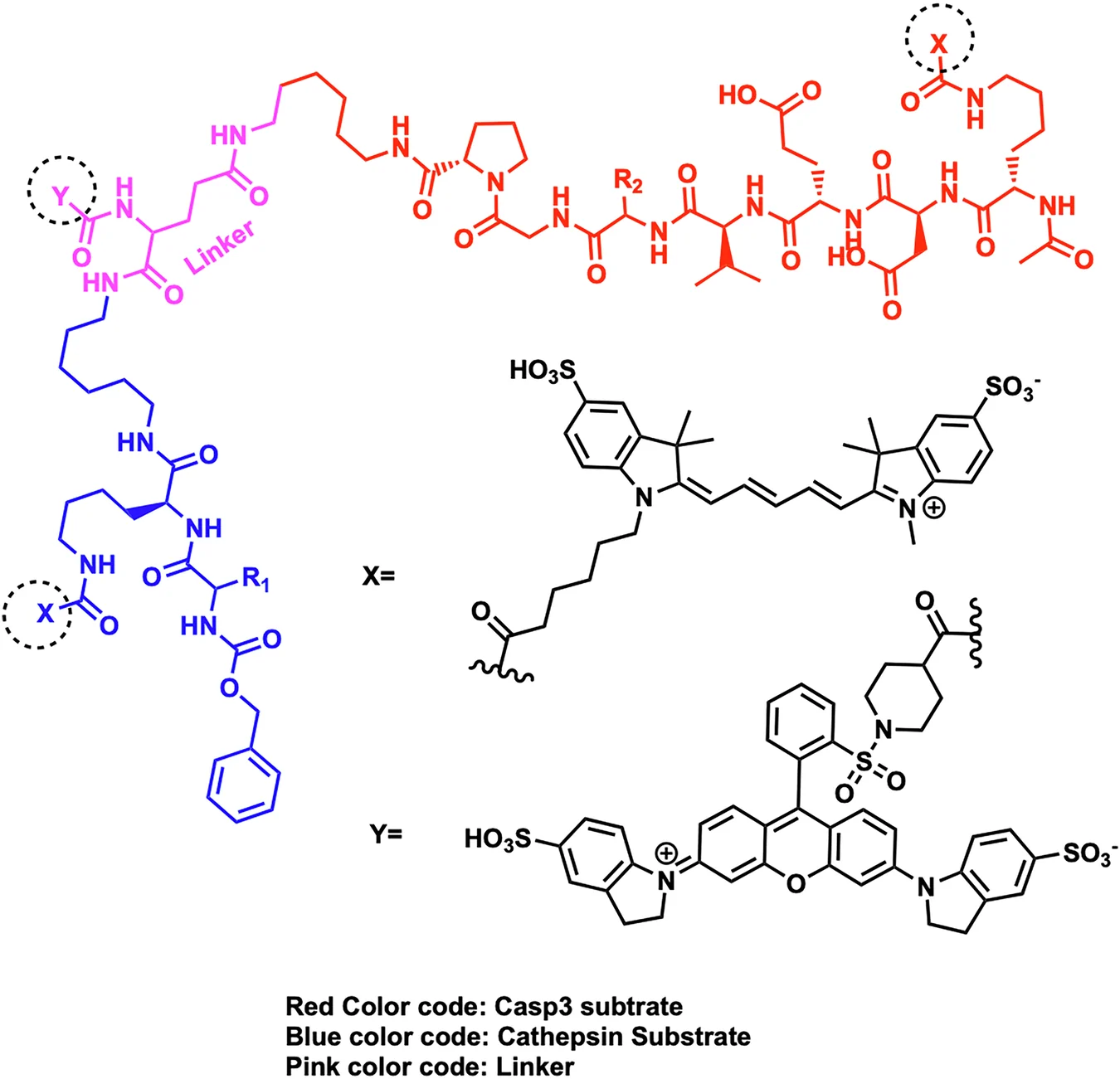
Structure of AND-gate probes. Structures of the dual orthogonal protease substrate probes.

Moving beyond single-target tumor imaging and phototherapy, recent probe design has increasingly focused on improving diagnostic precision through multi-input molecular recognition. The universal AND-gate design features intentional placement of a fluorescent reporter within a central hub, allowing for conjugation of multiple quenchers via enzymatically sensitive linkages. The ‘hub and spoke’ structure requires all attached quenchers on spokes to be removed for liberation of the central fluorescent hub. Conceptually, a central hub is coupled with multiple quenchers via multiple enzymatically sensitive linkages, creating AND-gates sensitive to distinct enzymatic signatures over a broad range. AND-gate fluorescent imaging probes were further designed by Widen et al. by combining two steps, namely, protease activation and fluorescence generation, using biorthogonal chemistry. The scientific rationale behind this method is based on the biological fact that tumor tissue often possesses different yet similar proteases, and thus activation by one type can cause nonspecific background signals. Peptide substrates were optimized to utilize reported specificity for caspase 3 (Casp3; DEVDGP) and cysteine cathepsin (z-FK) processing. Construction of the AND-gate probe, FAP-CAT, was accomplished using consistent chemistries and linkages for cathepsin and fibroblast activation protein α (FAP) sequence discrimination. The structural organization of FAP-CAT includes a central D-Glu linker with a sulfo-Cy5/sulfo-QSY21 fluorophore/quencher arrangement. Activation of the FAP-CAT probe is dependent only on incubation with FAP or CATL, with no discernible response observed for single proteases. Efficacy of these probes was evaluated in mammary tumor models, metastatic lung cancer models, and even in robotic fluorescence-guided surgery, showing their relevance for intraoperative tumor visualization. (Fig. 25)^194^.

**Fig. 26:**
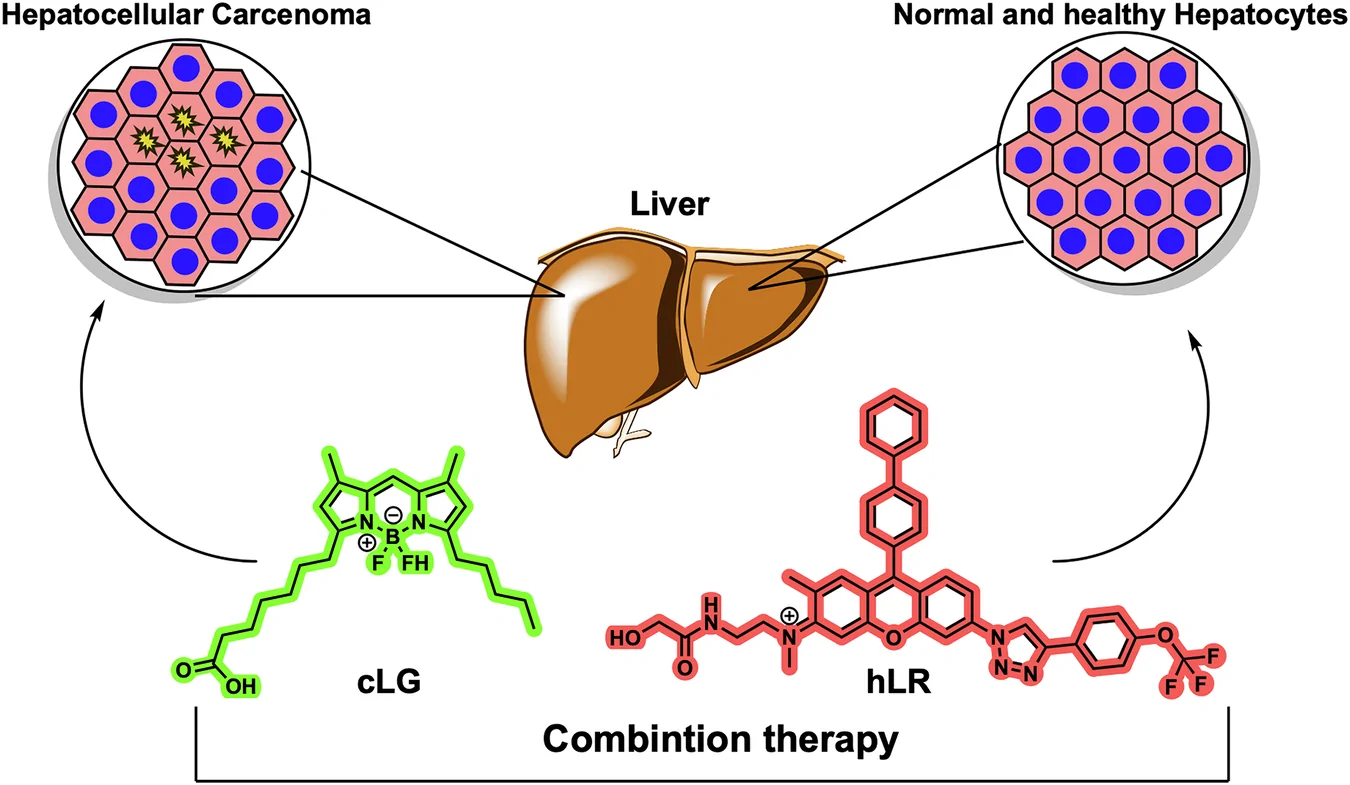
Discovery of fluorescent probes cLG and hLR for HepG2 and THLE-2 cells. Schematic representation of fluorescent probes for selective detection of cancer and healthy cells.

Recent study developed a chemically stable fluorescent sensor based on far-red emitting silver nanoclusters (PEI-AgNCs) using polyethyleneimine (PEI) as the template and 2-mercaptobenzothiazole (MBT) and sodium citrate (SC) as reducing agents for the selective determination of folic acid and targeted imaging of cancer cells with overexpressed levels of folate receptors. The efficient baseline separation of excitation and emission wavelengths minimizes background interference to greatly improve the contrast of bioimaging in complex biological systems. Characterization of the sensor showed its spherical shape, with a peak emission intensity at 631 nm. The nanomaterial showed great potential for bioimaging applications by reducing interference from biological background fluorescence and alleviating light scattering effects. The fluorescence intensity in HeLa cells correlated with their expression levels of FRs, highlighting the sensor’s potential for imaging tumor cells selectively^195^. Extending this concept beyond folate receptor-positive tumors, oncology takes advantage of specificity provided by fluorescent probes through the targeting of receptors that are often overexpressed in neoplastic cells. Probes have, for instance, been developed to target the human epidermal growth factor receptor 2 (HER2), the epidermal growth factor receptor (EGFR), and folate receptors. Among different types of cancers, hepatocellular carcinoma is a prevalent liver cancer that is associated with high rates of both morbidity and mortality. A fluorescent imaging probe, which has been labeled cLG, along with a similarly related probe labeled hLR, has been engineered to identify malignant cells from normal cells in the imaging of liver tissue. The probes have been shown to selectively tag particular populations of cells, outline the borders of tumors, and identify neoplastic vs. normal tissue, which implies that these may have potential for fluorescence-guided surgery applications (Fig. 26)^196^.

### Infectious disease detection and image-guided diagnosis

Fluorogenic probes are promising diagnostic tools that exploit unique biochemical properties in enabling quick and accurate infection diagnosis. In recent years, fluorescence detection technology has gradually emerged in the application of virus and bacterial detection^197,198^. Bioimaging reporters are gaining attention in the therapeutic avenue and showing promise. In instances of diseases like tuberculosis (TB) and COVID-19, fluorescent probes that target bacterial enzymes or viral proteins have been synthesized. These probes ensure the visualization of pathogens in clinical samples, hence enabling early diagnosis and relevant therapy. For instance, fluorescent markers conjugated with peptides have been used to detect TB bacteria, with the diagnosis available in only minutes and helping prevent the spread of infection^199^. An economically feasible point-of-care TB diagnostic system has been developed based on the combination of catalytic hairpin assembly (CHA) and the amplification of two signals using recombinase polymerase amplification (RPA). CdTe QDs are employed for visual or homogeneous fluorescence diagnostic signaling. TMA-capped QDs have greater detection sensitivity due to enhanced binding capacities for the silver ion (Ag+). The protocol developed was successfully applied to the clinical sputum samples of 36 TB patients and 20 healthy individuals, generating quantifiable data consistent with that from real-time RT-qPCR^200,201^.

Recent studies conducted by Sun et al. describe the development of an innovative method for the disinfection of harmful microorganisms with the use of a biomimetic silver-binding peptide, AgBP2. AgBP2-Ag_2_S QDs, which are capped with AgBP2, show high fluorescence properties. With the application of 808 nm near-infrared laser, it has been shown that the ROS generated can be used for the disinfection of harmful microorganisms, with 99.06% efficiency for the disinfection of Escherichia coli after 25 minutes. This shows that the disinfection process can be effectively carried out with the use of this quantum dot. In addition, the extended circulation time and biodistribution of Ag_2_S QDs can be further improved with the incorporation of biocompatible polymers, which can be useful for the development of dynamic imaging and diagnostic tools^191^.

The COVID-19 pandemic highlighted the need for point-of-care diagnostics that are low-cost, reliable, and suitable for self-testing. A novel approach to simple, inexpensive lateral flow assays (LFAs) was developed using glow stick peroxyoxalate chemistry. The use of fluorescent probes in fluorescent lateral flow assays (LFAs) has significantly enhanced point-of-care (POC) diagnostic technologies’ sensitivity and specificity. Fluorescent LFAs enable the detection of trace biomarkers, hence proving their significance in situations where there is limited availability of laboratory resources. During the COVID-19 pandemic, fluorescent LFAs played a critical role in massive testing measures through the provision of quick and accurate verification of the virus. Ju et al. developed an AIE carbon dot-based fluorescent lateral flow immunoassay for rapid detection of SARS-CoV-2-specific IgM and IgG antibodies. The AIE-CDs showed dual fluorescence emission and high emission efficiency in aggregated/solid states, making them suitable fluorescent labels for immunochromatographic detection. The platform achieved simultaneous detection of SARS-CoV-2 IgM and IgG with a reported detection limit of 100 pg/mL and showed selectivity against influenza A and B antibodies. This work combines the simplicity of point-of-care lateral flow testing with enhanced fluorescence sensitivity, supporting rapid serological screening, immune-response monitoring, vaccine-response evaluation, and public-health surveillance during viral outbreaks^202^. These findings have highlighted the need for the utilization of fluorescent probes in controlling the spread of infectious disease outbreaks. The NIH Rapid Acceleration of Diagnostics program aimed to develop a deployable lateral flow diagnostic targeting the nucleoprotein of SARS-CoV-2. Over 250 antibody pairs were tested for analytical sensitivity and specificity in the context of LFA. The development of this technology included excitation liquid optimization, design of a custom cartridge, development of a smartphone reader, and development of a respective app. This technology demonstrated excellent preclinical performance and was poised for commercialization. Although not commercialized yet, the platform was successful in achieving significant proof-of-concept and advancing in translational elements, including early high-performance LFAs and practical packaging of excitation liquid^203^. During the COVID period, a study from the Singamaneni research group demonstrates that plasmonically active, fluorescent gold nanorods conjugated to antibodies can greatly increase the sensitivity of conventional lateral-flow assays. When used with a common benchtop fluorescence scanner, the nanorods achieve a 30-fold improvement in dynamic range and detection limits, leading to 95% clinical sensitivity and 100% specificity for antibodies and antigens in SARS-CoV-2 samples. The new technology promises to bring benefits to point-of-care diagnostic uses^204^.

**Fig. 27:**
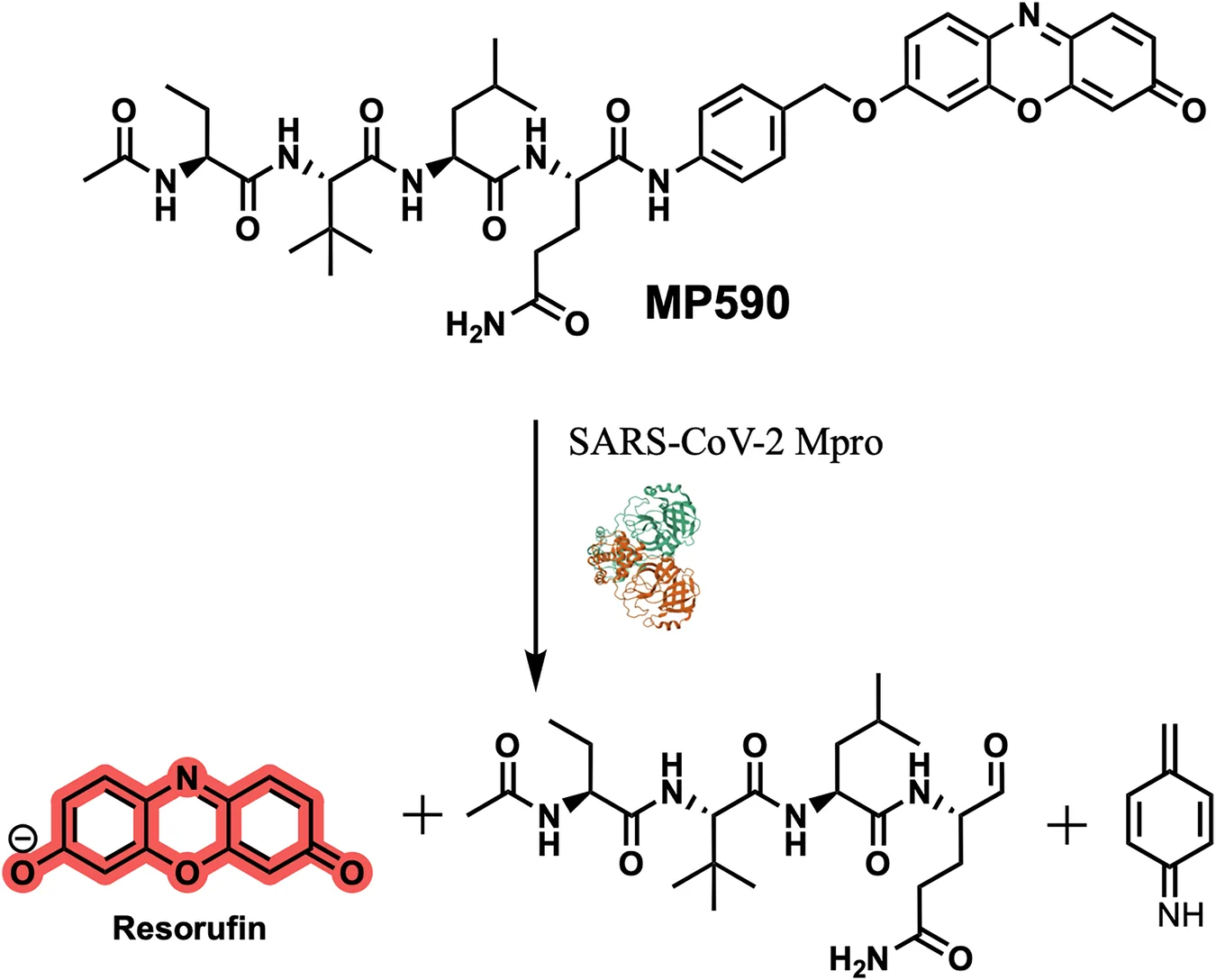
Self-Immolative fluorescent probe for selective detection of SARS-CoV-2 main protease. Detection mechanism of MP590.

**Fig. 28:**
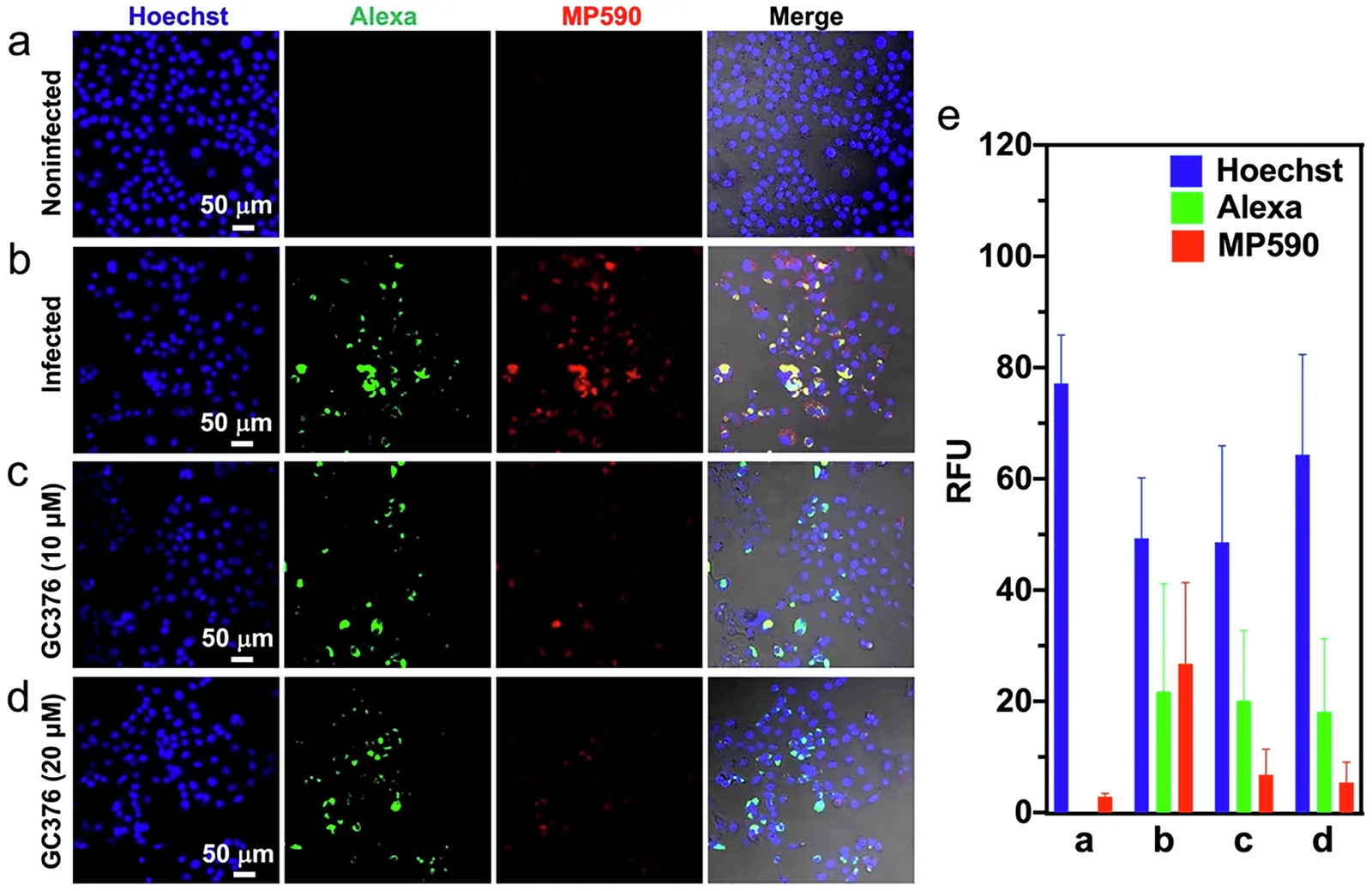
Visualization of Mpro expression and MP590-mediated Mpro inhibition activity in SARS-CoV-2-infected cells. TMPRSS2-VeroE6 cells. TMPRSS2-VeroE6 cells were treated under the following conditions: **a** uninfected TMPRSS2-VeroE6 cells as the control, **b** TMPRSS2-VeroE6 cells infected with SARS-CoV-2, **c** SARS-CoV-2-infected TMPRSS2-VeroE6 cells treated with 10 μM of the commercial Mpro inhibitor GC376, and **d** SARS-CoV-2-infected TMPRSS2-VeroE6 cells treated with 20 μM GC376. **a** Uninfected TMPRSS2-VeroE6 cells displayed only very weak fluorescence signals. In contrast, **b** shows that SARS-CoV-2 infection markedly enhanced fluorescence signals in both the Mpro antibody (Alexa) channel and the MP590 channel, indicating detectable Mpro expression/activity in infected cells. As shown in (**c**), treatment of infected cells with 10 μM GC376 resulted in a clear reduction in fluorescence intensity in both the Mpro antibody (Alexa) and MP590 channels. A further decrease in fluorescence signal was observed in (**d**) when the concentration of GC376 was increased from 10 to 20 μM, confirming concentration-dependent inhibition of Mpro-associated fluorescence. **e** Quantitative analysis showing the mean fluorescence intensity with standard deviation represented as error bars, calculated from CLSM images acquired from 12 fields of view corresponding to (**a**–**d**). All cell groups were incubated with MP590 at 3 μM and stained with SARS-CoV-2 Mpro primary antibody followed by Alexa-labeled secondary antibody. Reproduced with permission^205^.

Further study on a self-immolative fluorescent probe for selective detection of SARS-CoV-2 main protease showed how an optically self-reporting probe, known as MP590, has been designed for targeted detection of SARS-CoV-2 Mpro. The probe is highly selective upon testing against a series of protein interferences, which include hemoglobin, BSA, thrombin, amylase, PLpro, and trypsin. Imaging of Mpro is made feasible by the probe in HEK 293 T plasmid-transfected and TMPRSS2-VeroE6 SARS-CoV-2-infected cells. The probe activation allows for detection of infected cell Mpro, with a decrease in fluorescence intensity upon addition of GC376. The probe also clearly delineates the level of Mpro inhibition in infected cells treated with varied concentrations of GC376 (Figs. 27, 28)^205^.

### Neurological disorders detection and image-guided diagnosis

The rising occurrence of neurological conditions among the elderly is putting a substantial economic burden on almost every household. Neurodegenerative diseases are a group of conditions that deteriorate progressively and systematically impair the nervous system. These conditions normally have a long development phase, with their effects often appearing much later in life^206^. In many neurodegenerative disorders, amyloid accumulation has been recognized to be a common pathogenic change. This process leads to the development of amyloid fibrils and amyloid plaques, which gradually impair neuronal integrity and strongly impair the function of the brain. In neurology, the use of fluorescent probes has substantially improved our insights into the complex biochemical landscape of the brain. Probes selective for certain neurotransmitters like dopamine and serotonin have been developed, allowing for the investigation of neuronal signaling and its dysregulation in diseases like Parkinson’s disease and depression. In addition, probes targeting pathological proteins like amyloid-β and tau have been critical to investigations into Alzheimer’s disease. Probes can visualize the accumulation of protein aggregates in the brain, helping to diagnose and monitor the course of the disease^207^.

**Fig. 29:**
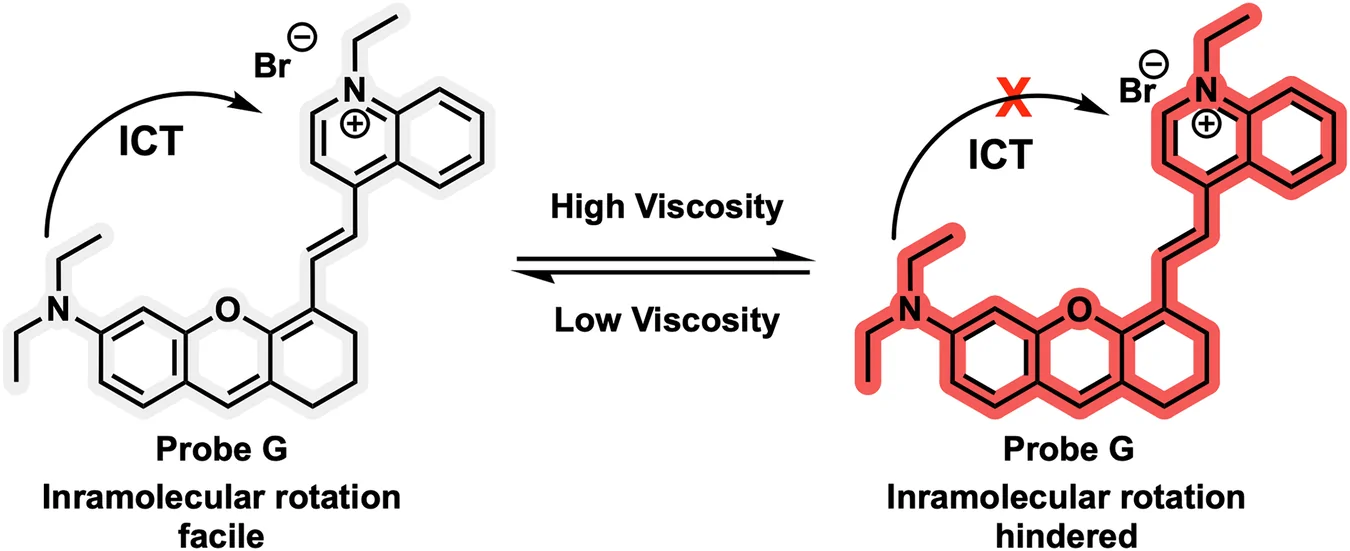
An oxanthene-based near-infrared fluorescence probe G for the detection of mitochondrial viscosity. Schematic representation fluorescent probe G, for mitochondrial viscosity and its application in the imaging of Alzheimer’s disease mouse brain.

**Fig. 30:**
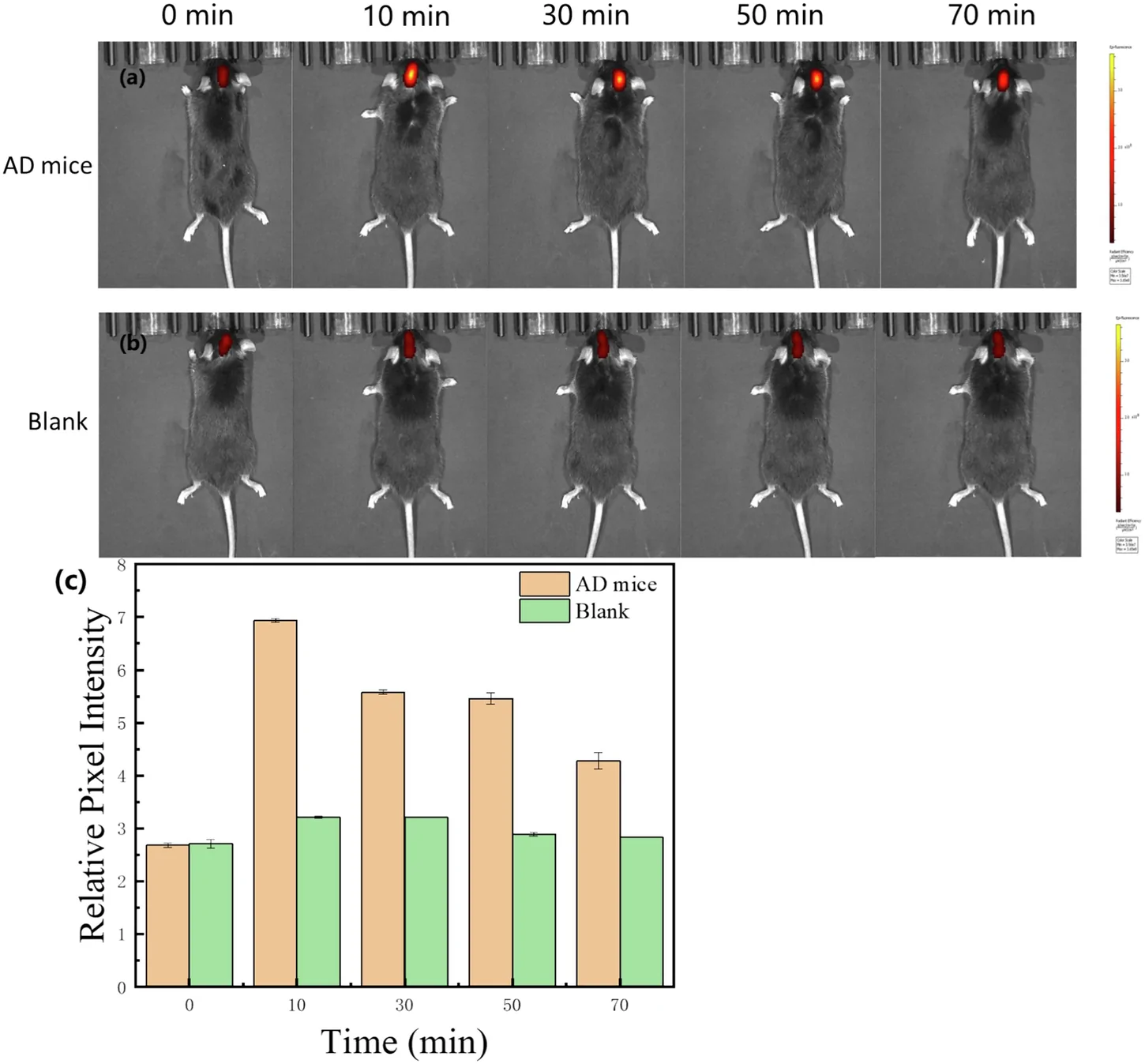
Fluorescence imaging of endogenous Aβ protein in AD mouse model. **a**–**b** Fluorescence imaging with Probe G in AD (**a**) and healthy (**b**) mouse brains after the injection of 50 μM, 50 μL for 0 to 70 min. λ_ex_ = 630 nm, λ_em_ = 700–740 nm. **c** Relative fluorescence intensities from AD (yellow bars) and healthy (green bars) mice brains. Reproduced with permission^208^.

A new anthraquinone-quinolinium-based near-infrared fluorescent probe **G** has been designed to measure the viscosity of the cellular microenvironment, an important factor in diseases like tumors, fatty liver disease, and Alzheimer’s disease. The probe showed high selectivity for viscosity and also targets the mitochondria. In mouse models, it showed its ability to quantify Aβ protein viscosity within the brains for Alzheimer’s disease (Figs. 29, 30)^208^.

AChE is a cholinergic enzyme primarily found in ppostsynaptic neuromascular junction, but its specific function is poorly characterized; however, AChE is implicated in both oncological and neurologic diseases. A new fluorescence probe, branded as CNA, has been synthesized for the fluorescence detection of AChE at 520 nm. A detection limit of 0.014 U/mL with a reaction time of 60 s characterizes the probe, highlighting its novelty. Having been successfully applied both in PC12 cells and in vivo mouse models, this probe has the potential to efficiently explore the physiological and pathologic functions of AChE in the complexities of biology^209^. In a similar vein, a simplified fluorophores coupled with MnO₂ nanosheets have shown excellent potential for precise and specific detection of biomarkers acetylcholinesterase (AChE). Simplified fluorescent tags, along with MnO_2_ nanosheets, have been used for the development of a ratiometric fluorescence sensing device for the detection of acetylcholinesterase (AChE) activity. This device has the capability for the detection of two different types of fluorescence, which improves the overall detectability. This has shown immense potential for the detection of AChE activity and the screening of inhibitors, e.g. Huperzine-A inhibitor. Finally, the unique properties of the MnO2 nanosheets, which act as a catalyst, have been utilized for the development and incorporation of rudimentary fluorescence indicators for the development of a ratiometric fluorescent device for the detection of AChE activity. This work proposes a ratiometric fluorescence sensing system for AChE detection that outperforms the standard single-channel fluorescence sensing detectors F-PDA-MnO_2_ and OPD-MnO_2_ in terms of quantitative range and the visibility of the color change. Additionally, the approach offers a low LOD of 0.073 U·L −  1 and a broad linear range, allowing for the quantitative assessment of AChE activity. Huperzine-A were used as an example to illustrate the potential use in inhibitor screening. In short, due to its potential, this approach might be useful for clinical AChE diagnosis and inhibitor screening^210^.

**Fig. 31:**
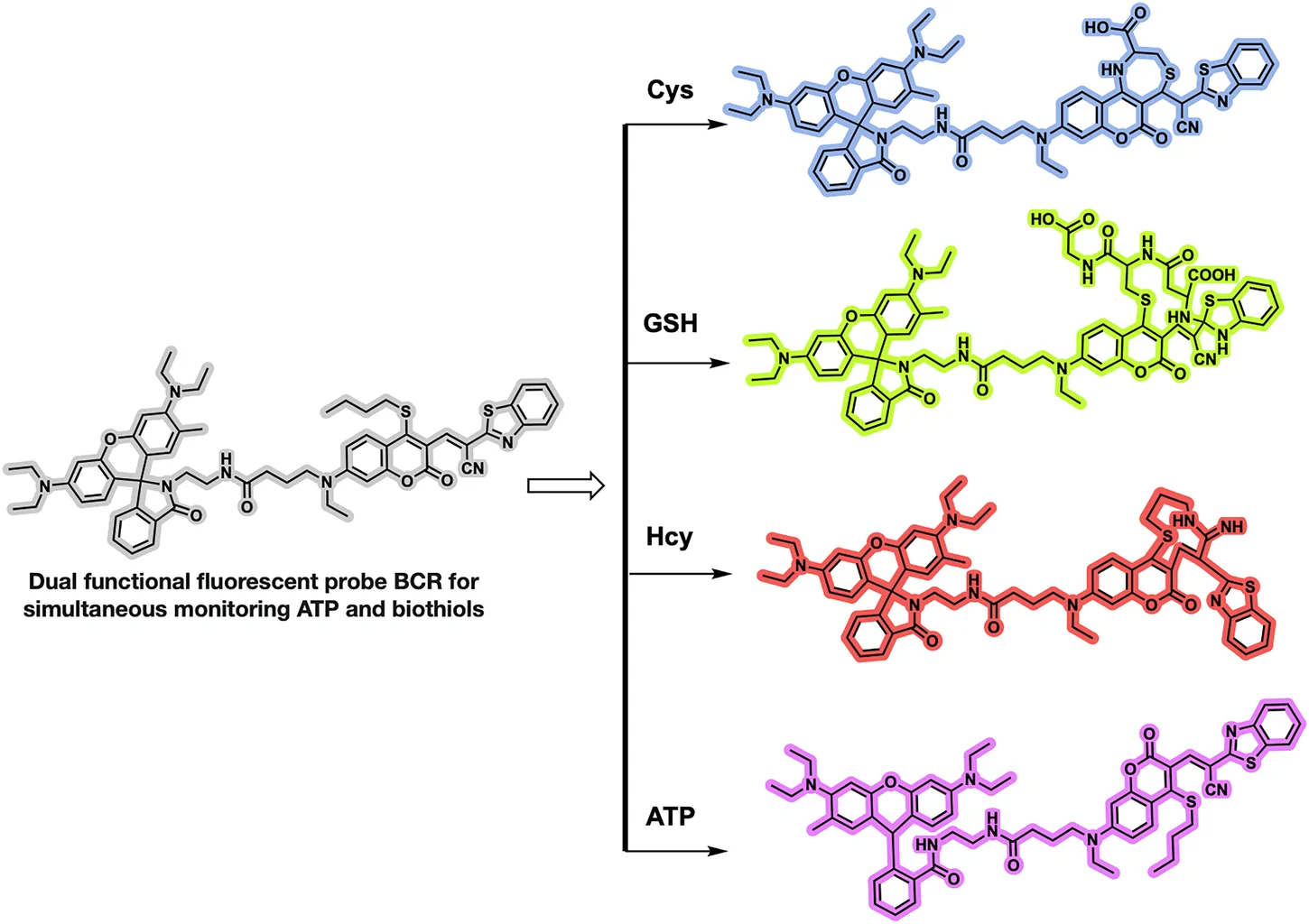
Sensing mechanism of probe BCR with cysteine, homocysteine, glutathione and ATP. Schematic representation fluorescent sensing of probe BCR solution after adding GSH, Hcy, Cys and ATP in epilepsy and liver injury models.

**Fig. 32:**
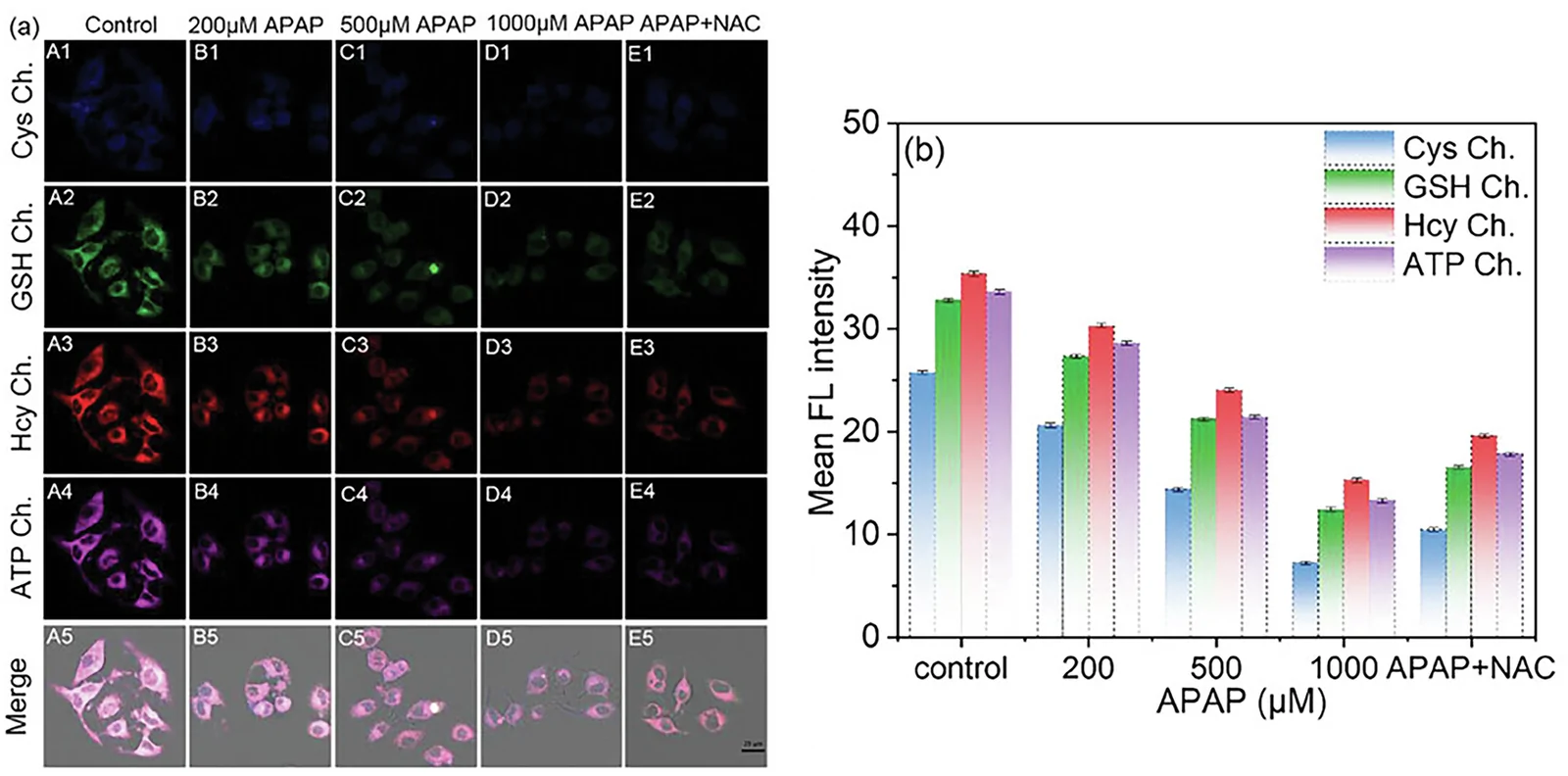
Confocal fluorescence imaging of GSH, Hcy, Cys, and ATP in HepG2 cells. Confocal fluorescence imaging of GSH, Hcy, Cys, and ATP in HepG2 cells. Cells were incubated with different concentrations of acetaminophen (0, 200,500, and 1000 µm), and APAP (1000 µm) + NAC (400 µm) for 12 h. **b** Relative pixel intensity of the fluorescence images A1-E5 in (**a**). λ_ex_ = 405 nm, λ_em_ = 420–470 nm for the blue channel, λ_ex_ = 458 nm, λ_em_ = 500–560 nm for the green channel, and λ_ex_ = 488 nm, λ_em_ = 540–630 nm for the red channel, and λ_ex_ = 514 nm, λ_em_ = 580–650 nm for the pink channel. Scale: 25 µm^211^.

Another important factor for neurological diseases is the regulation of adenosine triphosphate (ATP). ATP is an important intracellular currency of energy that is vital for a wide variety of cellular functions and is closely related to many pathological conditions. Changes in the concentration of ATP have been linked to many neurodegenerative conditions like epilepsy, Parkinson’s disease and Alzheimer’s disease. These diseases are mainly linked to pathological oxidative stress and disturbances in the process of metabolism of energy. Glutathione (GSH), cysteine (Cys), and homocysteine (Hcy) biothiols are critical for various physiological actions, as well as several pathological processes, due to their desirable redox properties. The two-step differential discrimination of biothiols and molecules of ATP is useful in the study of epilepsy pathophysiology and hepatotoxicity. A variety of novel methods for obtaining spatiotemporal information about extracellular ATP and adenosine, such as fluorescent sensors, have been developed so far and is beginning to elucidate the mechanisms involved in the release, uptake, and breakdown of ATP and adenosine. Herein, the novel fluorescence probe BCR is presented for simultaneous detection of Hcy, GSH, ATP, and Cys. The probe BCR is noted for its high optical properties, high biocompatibility, high selectivity, and enhanced sensitivity, useful for effective in vivo imaging of biothiol dynamics of ATP. Most importantly, the probe BCR efficiently visualizes the changes in the levels of Hcy, GSH, ATP, and Cys in both the zebrafish model of PTZ-induced epilepsy and the APAP-induced liver toxicity model in mice. These findings demonstrate the high potential of the probe BCR for early disease detection, along with potential therapeutic applications (Figs. 31, 32)^211^.

Many important biomarkers have been recognized for their correlation with neurological disorders. The presence of amyloid-β (Aβ) plaques, along with tau protein tangles, has been established to be definite markers for Alzheimer’s disease. The activity of acetylcholinesterase (AChE) also stands as an important marker most commonly linked with cognitive impairment. Several other physiological and molecular parameters are inherent to the etiology and progression of these multidirectional diseases. Additionally, neuroinflammatory markers like the cytokines IL-6 and TNF-α, markers for oxidative stress like reactive oxygen species (ROS), and the disruption of ions like Zn²⁺, Cu²⁺, and Fe³⁺ form significant parameters of this discourse^212,213^. Furthermore, fluctuations in the levels of neurotransmitters e.g. dopamine, glutamate, and GABA, as well as the loss of ATP are representative of cellular malfunction in Parkinson’s and Huntington’s diseases. These markers provide critical information related to the progression, onset, and therapeutic response regarding these disorders^214^. Oxidative stress, metal ion imbalances, the presence of reactive oxygen and nitrogen species, and changes in the concentration of neurotransmitters are all indicators characteristic of neurodegenerative pathophysiology^215,216^. Consistent with this multifaceted fact, extensive research has been focused on the design of novel fluorescence sensors for the detection of these various markers with higher sensitivity and selectivity. These newly designed probes operate in real time, many with subcellular resolution, thus serving as vital tools for the early detection and mechanistic investigation of diseases like Alzheimer’s disease, Parkinson’s disease, and other neurodegenerative conditions^217^.

**Fig. 33:**
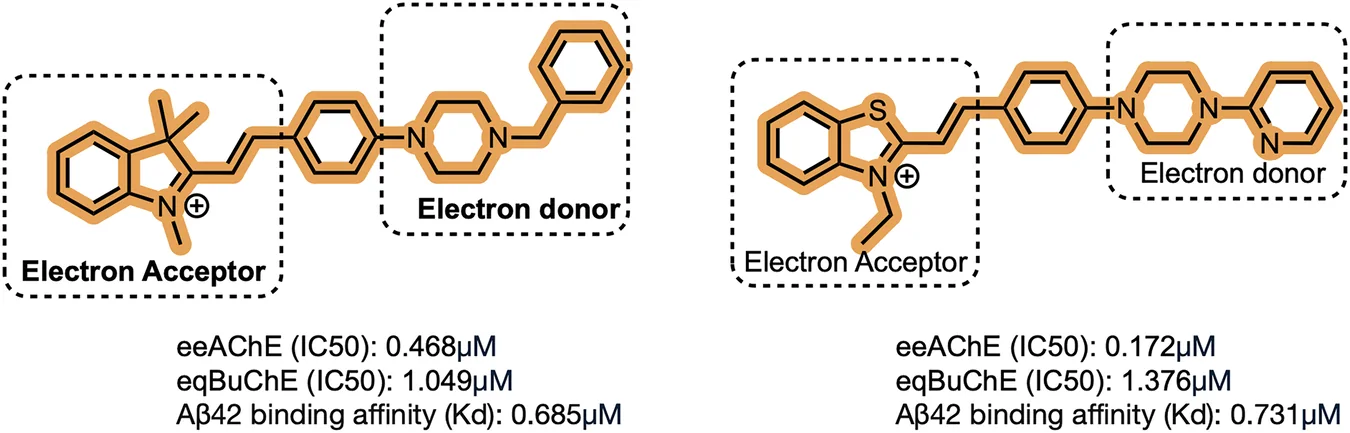
The donor-acceptor architecture type theranostic fluorescent probes. Schematic illustration of fluorescent molecules for AD with the potential to simultaneously perform detection of the Aβ species and inhibit the cholinesterase (ChE) enzymes.

The advancement of Alzheimer’s disease (AD) is ascribed to cholinergic deficiencies and the accumulation of β-amyloid (Aβ) species. A range of prospective theranostic drugs was formulated and assessed for their efficacy against cholinesterase enzymes and the identification of Aβ species. AChE is essential hydrolase in the neurological system associated with mood disorders resembling depression. A benzothiazolium fluorophore and a bifunctional electron-donating N-aryl piperazine scaffold-based fluorescent probe, demonstrated significant inhibitory efficacy against acetylcholinesterase (AChE) (AChE; IC_50_ = 0.172 ± 0.011 μM) and butyrylcholinesterase (BuChE) (BuChE; IC_50_ = 1.376 ± 0.141 μM). The fluorescence characteristics exhibited significant binding affinities and an augmented fluorescence response upon interaction with Aβ aggregates. Fluorophore Compound showed no indications of toxicity, and in vivo behavioral assessments revealed a significant enhancement in cognitive and spatial memory impairments in the scopolamine-induced cognitive impairment murine model. chemical/probe 18 exhibited outstanding characteristics as a fluorescent contrast agent and treatment efficacy, establishing it as a safe and effective lead chemical for Alzheimer’s disease (Fig. 33)^218^.

**Fig. 34:**
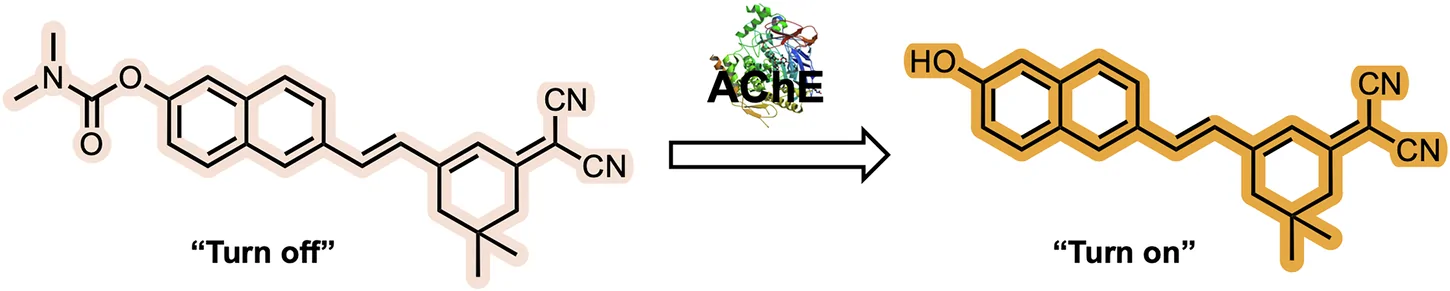
N,N-dimethylcarbamoyl group bearing probe ACP for monitoring acetylcholinesterase in Alzheimer’s disease (AD). Schematic illustration of “turn on” fluorescent molecules for selective imaging of the upregulation of acetylcholinesterase (AChE) in Alzheimer’s disease (AD).

ACP, a novel AChE-detecting fluorescent probe has been developed with a low detection limit of 0.341 U/mL. By using the probe, the expression of intracellular AChE was monitored in real time, and the upregulation of AChE expression has been detected in Alzheimer’s disease model. The design and synthesis of the probe are viable, shows excellent selectivity, and are sensitive to AChE, making it worthy of further exploration in various physiological and pathological conditions. Hence, the novel probe ACP may be a potential tool to be used to explore the physiological conditions related to AChE in the future. The fluorescent probe successfully imaged the dynamic changes of AChE activity and mitochondrial localization in PC12 and U87 cells and also showed good effects in detecting AChE changes in C. elegans of AD model and AD mice. Furthermore, a turn-on-type fluorescent probe CNA, has also been developed to detect AChE in biological samples, which shows a rapid response time of 60 s and a low detection limit of 0.014 U/mL. The probe has been used to detect AChE in PC12 cells and mice, making it a potential tool to explore the physiological and pathological role of AChE in biological systems (Fig. 34)^219^.

**Fig. 35:**
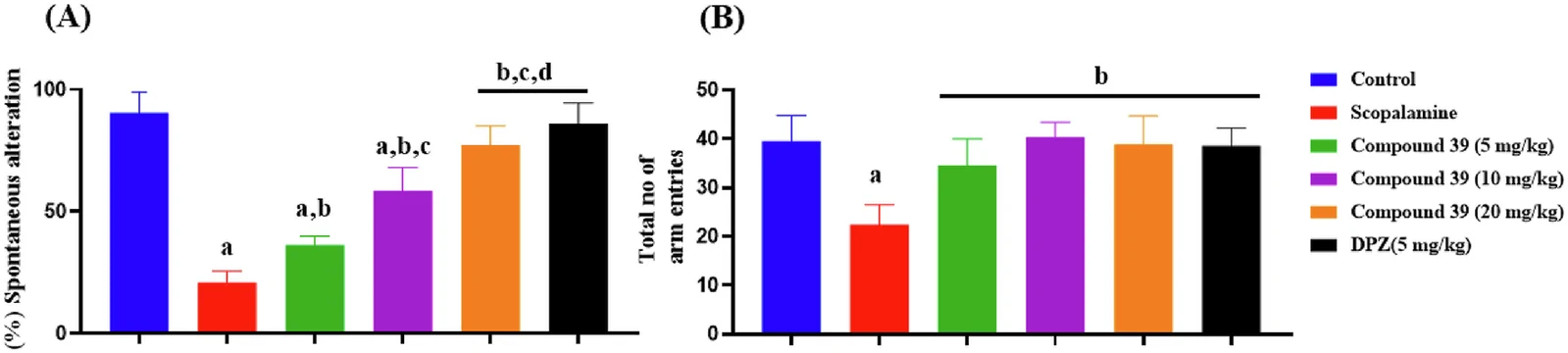
Schematic illustration of theranostic fluorescence probe 39 on cognition enhancement in the scopolamine-induced amnesia model. **A** Indicates % spontaneous alternation. **B** Indicates total no of arm entries. ^a^*P* < 0.05 vs. control group; ^b^*P* < 0.05 vs. scopolamine group; ^c^*P* < 0.05 vs. compound 39 (5 mg/kg) group; ^d^*P* < 0.05 vs. compound 39 (10 mg/kg). One-way ANOVA followed by Newman–Keuls post hoc test. Reproduced with permission^220^.

Most recently, an array of innovative theranostic compounds was developed and assessed for their efficacy against cholinesterase (ChEs) enzymes and the detection of Aβ species, which are primary targets in the therapeutic advancement for Alzheimer’s disease (AD). Compounds 39 and 43 had significant inhibitory effects on ChE enzymes, with expected superior BBB permeability in the PAMPA experiment. Fluorescence emission patterns exhibited notable characteristics across several spectroscopic parameters. In DMSO absorption maxima were found at 440 nm and emission spectrum with maxima displayed at wavelength of 640 nm. The binding experiment demonstrated that probe 39 interacts with Aβ_1−42_ aggregates and exhibits an increase in fluorescence intensity. The binding affinity for Aβ_1−42_ aggregates was confirmed by colocalization with thioflavin T (ThT). The mechanism of the fluorescence response was also characterized in detail using DFT computational simulations. The acute toxicity studies did not show any signs of toxicity and did not reveal any changes in body weight, food intake, and water consumption. Behavioral studies in vivo also revealed a marked improvement in cognitive and spatial memory deficits in the scopolamine-induced cognitive impairment mouse model. The ex vivo studies confirmed the brain acetylcholinesterase inhibitory potential of the lead compound 39, as indicated by decreased acetylcholinesterase enzyme level and increased acetylcholine level, along with significant antioxidant potential. This paper describes the first theranostic fluorescent probe for the simultaneous detection of amyloid beta aggregates and cholinesterase enzyme inhibition, which is a major target for the treatment of Alzheimer’s disease (Fig. 35)^220^.

**Fig. 36:**
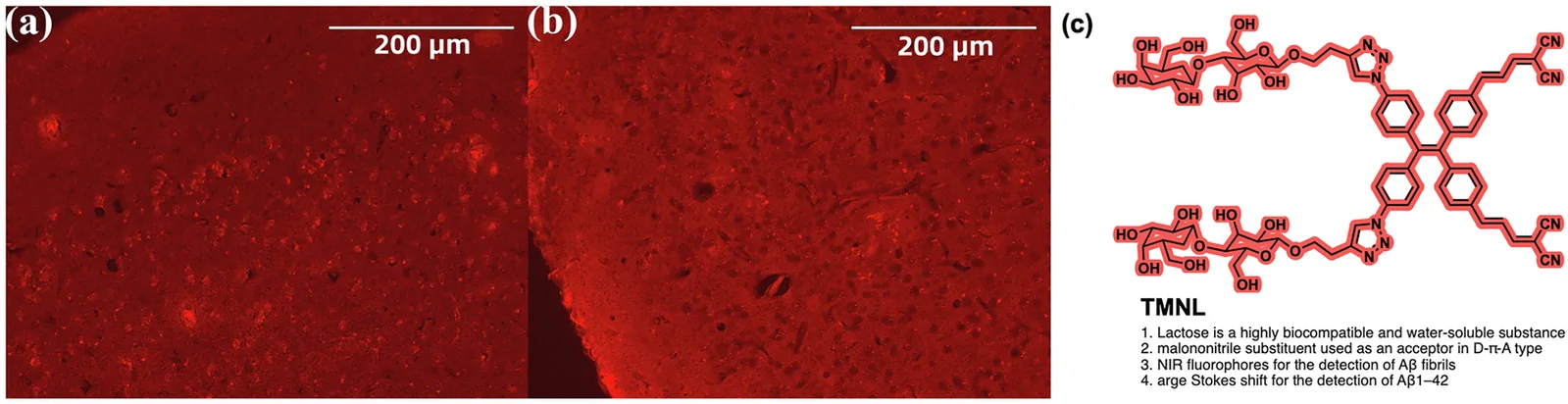
Fluorescence staining images of TMNL. **a**, **b** TMNL-stained Aβ1–42 plaques in the brain tissue of AD transgenic mice. **c** Molecular structure of TMNL. Reproduced with permission^221^.

Ji et al. have synthesized an AIE-based NIR fluorescent probe that exhibits good water solubility to detect Aβ_1−42_ aggregates in Alzheimer’s disease. In the present work, a bis-lactosyl-functionalized tetraphenylethene derivative, TMNL, based on the AIE effect, has been synthesized. Its amphiphilic character allows for its self-aggregation into emission-enhanced fluorescent organic nanoparticles. This probe has shown its potential in the imaging of Aβ_1–42_ aggregates in a transgenic mouse model of Alzheimer’s disease (Fig. 36)^221^.

Zhang et al. describe artificial nanoreactors designed specifically to mimic natural processes of photosynthesis to achieve in situ photocatalytic generation of hydrogen activated by light. The nanoreactors are made up of cross-linked biocompatible vesicles embedded with ascorbic acid, chlorophyll a, and indoline dye, which release hydrogen gas upon irradiation with NIR light. The process is also effective for eliminating high levels of reactive oxygen species (ROS) and preventing tau hyperphosphorylation in mouse models for AD, attesting to a proof-of-concept for using hydrogen therapy for AD^118^.

**Fig. 37:**
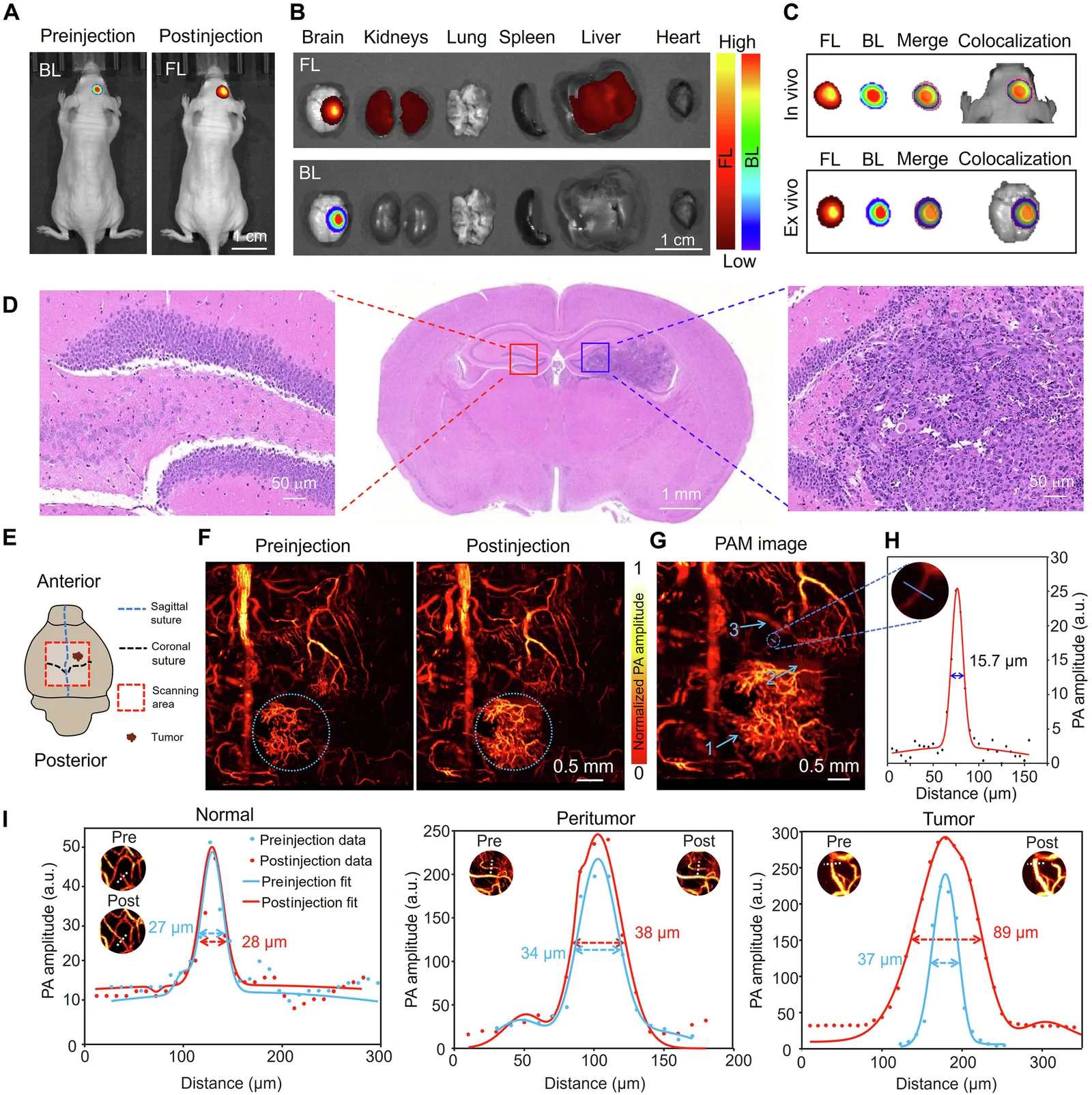
In vivo photoacoustic imaging of GBM. **A** In vivo bioluminescence (BL) and fluorescence (FL) images of GBM-bearing mice acquired 1 h after intravenous administration of IVTPO. **B** Ex vivo FL and BL images of the major organs collected from GBM-bearing mice 1 h after intravenous injection of IVTPO. **C** In vivo and ex vivo colocalization analysis of BL and FL imaging signals. **D** Hematoxylin and eosin (H&E) staining of brain tissue from the GBM-bearing mouse model. **E** Schematic representation of the tumor location, mouse skull structure, and photoacoustic microscopy (PAM) imaging region. **F** Photoacoustic images of cerebral blood vessels in a GBM-bearing mouse before and after intravenous injection of IVTPO. **G** Representative photoacoustic image of cerebral vessels in a GBM-bearing mouse after IVTPO administration, indicating the tumor region (1), dispersed probe signal (2), and cerebral cortical vessel (3). **H** Cross-sectional intensity profile measured along the blue line shown in (**G**). **I** Changes in blood vessel diameter in normal, peritumoral, and tumor regions before and after IVTPO injection. Reproduced with permission^223^.

One of the biggest challenges facing the neuroimaging community is the presence of the blood-brain barrier (BBB), which hinders the entry of diagnostic agents into brain^222^. The amphiphilic hemicyanine dye IVTPO, which is noted for its impressive optical properties and high potential to cross the blood-brain barrier, has been shown to serve as an effective fluorescence/photoacoustic probe for glioblastoma (GBM). IVTPO exhibits outstanding light imaging properties along with enhanced efficiency in the targeting of tumors. More importantly, the study found that in GBM progression, IVTPO was able to detect irregularity in the leakage of probes around the tumor-associated vascular structures even before the disruption of the BBB could be seen using Evans blue. This imaging tool also showed the presence of other changes associated with tumor vessels, such as dilation of the vascular structures inside the tumor tissue. Based on its performance when compared to Evans blue, IVTPO showed superior efficiency. These observations point to beneficial uses in the clinical diagnosis of intracranial GBM (Fig. 37)^223^.

These agents improve diagnosis of neurological disorders while serving as drug delivery carriers as well. For example, dual-functioning probes have been developed to image amyloid plaques while, at the same time, delivering therapeutic agents, which opens the door to an integrated diagnosis and therapy of neurodegenerative diseases^224,225^. Besides acting as a diagnostic and therapeutic tool in neurodegeneration, fluorescent probes have also been found to be useful as an invaluable tool in analyzing signaling processes in neurons. Whereas the dual-function exogenous probes are mainly used for the diagnosis of diseases and for therapeutic purposes, genetically encoded fluorescent sensors provide an alternative approach whereby they facilitate visualization of cellular activities within a live cell. An important class of genetically encoded sensors includes calcium indicators, of which a representative is the GCaMP family^226^. They contain a circularly permuted GFP, which is linked with calmodulin and a calmodulin-binding M13 peptide. The interaction with calcium elicits a conformational change, which then leads to an increase in GFP fluorescence. These probes have become invaluable tools for neuroscientific studies, allowing for real-time recording of neural activity and calcium events within a living organism. Genetically encoded voltage indicators (GEVIs) and neurotransmitter sensors, including glutamate sensor iGluSnFR, are also responsible for significantly advancing our understanding of synaptic transmission, plasticity, and network dynamics^227,228^. Quantification of calcium ion (Ca^2+^)-dependent fluorescence using genetically encoded calcium indicators (GECIs) has become a widespread approach for measurement of neuronal activity at both the level of the individual neuron and more extensive brain networks. Improvements toward greater sensitivity in GECI design over recent years have led to novel approaches for in vivo imaging. The GFP-based GCaMP sensors were systematically optimized to increase the signal-to-noise ratio (SNR) for Ca2+ influx detection upon neuronal activation. In favorable conditions, GCaMP6 and jGCaMP7 sensors allow for detection of an individual action potential (AP) and are often used for recording from extensive groups of neurons with techniques such as two-photon microscopy or wide-field image fluorescence. Furthermore, the sensors are also applied for monitoring calcium dynamics induced by activity within dense synaptic compartments such as dendritic spines and axonal regions. All jGCaMP8 versions are characterized by rapid kinetics upon variations in calcium concentration. Of all calcium indicators, jGCaMP8 attains a maximal single-spike sensitivity, with a comparatively brief half-decay time value of 200 ms within a mouse brain^134^. Biomedical applications range from disease modeling to investigating neural circuit function, as well as studies on epilepsy and neurodegenerative diseases. Other than that, inorganic nanomaterials enable the creation of multifunctional imaging platforms through the coupling of fluorescence with other modalities, such as magnetic resonance imaging (MRI), computed tomography (CT), and photoacoustic imaging. For instance, gadolinium-doped rare-earth nanoparticles combine both NIR fluorescence and T1-weighted MRI contrast, thus enabling dual-modality imaging for oncological and neurological applications. Similarly, gold or silver nanoparticles can be coupled with fluorophores to create hybrid platforms that take advantage of both plasmonic amplification and NIR emission^229^. Summing it all, fluorescent probes have become critical tools in modern diagnostics, yielding real-time, high-resolution information relevant to many diseases.

### Liver disease detection and image-guided diagnosis

The liver serves as an important organ for achieving body homeostasis through the processes of metabolism of nutrients, xenobiotic detoxification, synthesis of lipids and cholesterol, immune response, and endocrine function^230^. Considering its multiple physiological roles, liver injuries can be caused by many reasons, which include liver injuries induced by drugs or chemicals, hepatic ischemia/reperfusion injury, viral hepatitis, non-alcoholic fatty liver disease, liver fibrosis, cirrhosis, and hepatocellular carcinoma^231^. These types of liver diseases are varied in terms of their origins and mechanisms of progression; however, they often share many common pathological characteristics, among which include oxidative stress, inflammation, metabolic disturbance, enzyme abnormalities, lipid deposition, fibrogenesis, and tumor formation^232^. It is therefore of great importance to diagnose these conditions as early as possible to significantly improve patient survival rates. The use of fluorescent probes represents a guidance tool to detect liver diseases at the biomarker level prior to tissue lesions becoming clinically evident and attests to its significance in early disease detection and treatment monitoring.

**Fig. 38:**
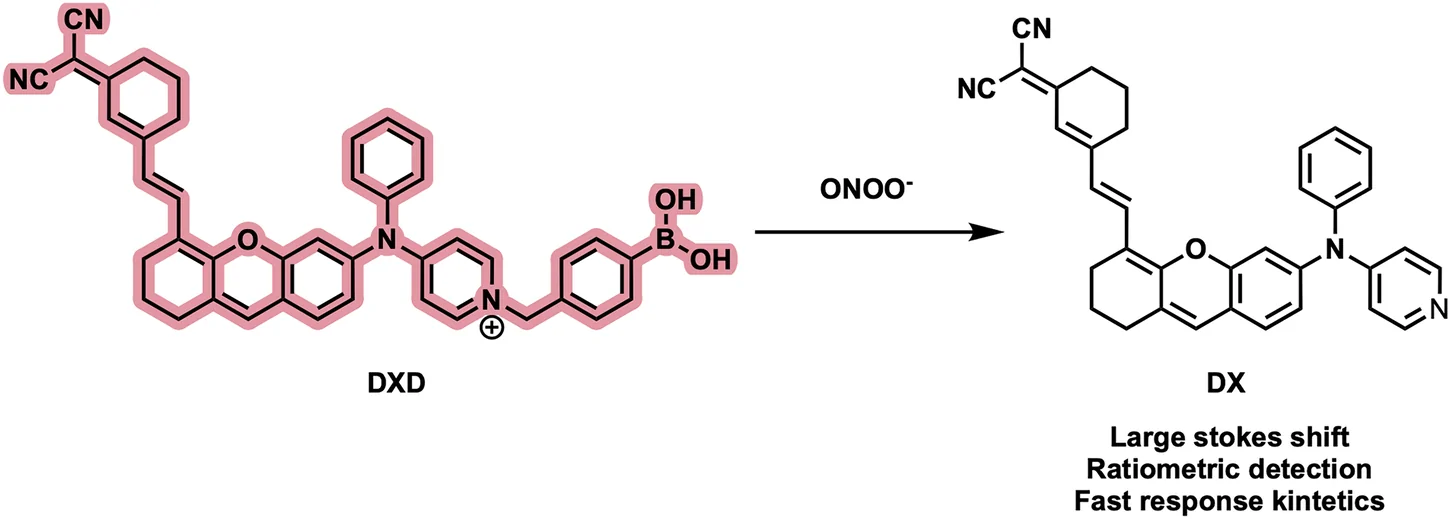
xanthene fluorophore DXD for ratiometric detection of ONOO−. Schematic representation of the reaction mechanism of DXD to ONOO^−^.

**Fig. 39:**
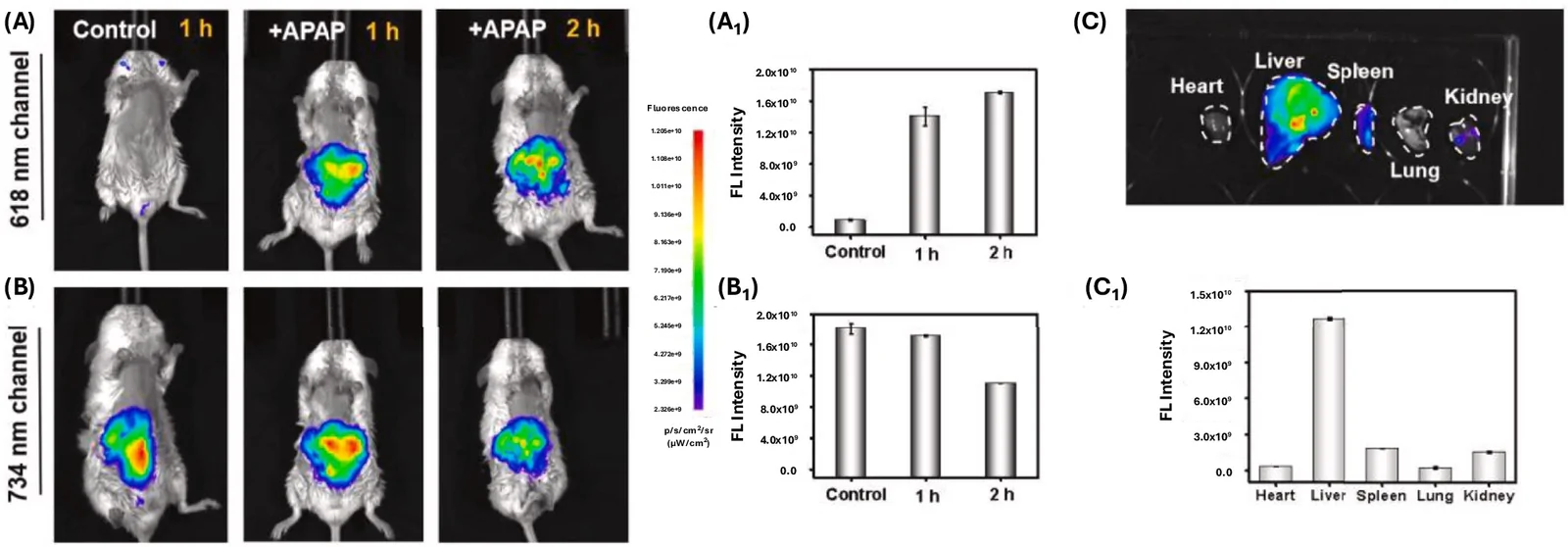
In vivo fluorescence imaging of endogenous ONOO− using an APAP-induced liver injury mouse model. **A**, **B**) Changes in ONOO− levels during DILI were monitored using dual-channel fluorescent imaging following tail vein injection of DXD (200 μM, 50 μL). **C** Fluorescence imaging of DXD in major organs (heart, liver, spleen, lung, and kidney). (A1-C1) Mean FL intensities of images in (A-C) ( ± S.D., *n* = 3). Reproduced with permission^233^.

Recently, Li et al. developed a ratiometric NIR fluorescent probe DXD for detecting ONOO^−^-fluxes for noninvasive visualization in models of rheumatoid arthritis (RA) and drug-induced liver injury (DILI) (Fig. 38). ONOO− is an essential product of nitric oxide (NO) and superoxide interaction and is tightly associated with oxidative/nitrosative stress, tissue inflammation, and hepatotoxicity. The work emphasizes the benefits of ratiometric probes in terms of correction of artifacts due to variations in probe concentration, tissue depth, excitation energy, and nonuniform biodistribution. Visualization of ONOO− levels during development of RA and DILI suggests the value of ratiometric fluorescent probes for the in vivo monitoring of inflammatory redox status, disease progression, and treatment response (Fig. 39)^233^.

**Fig. 40:**
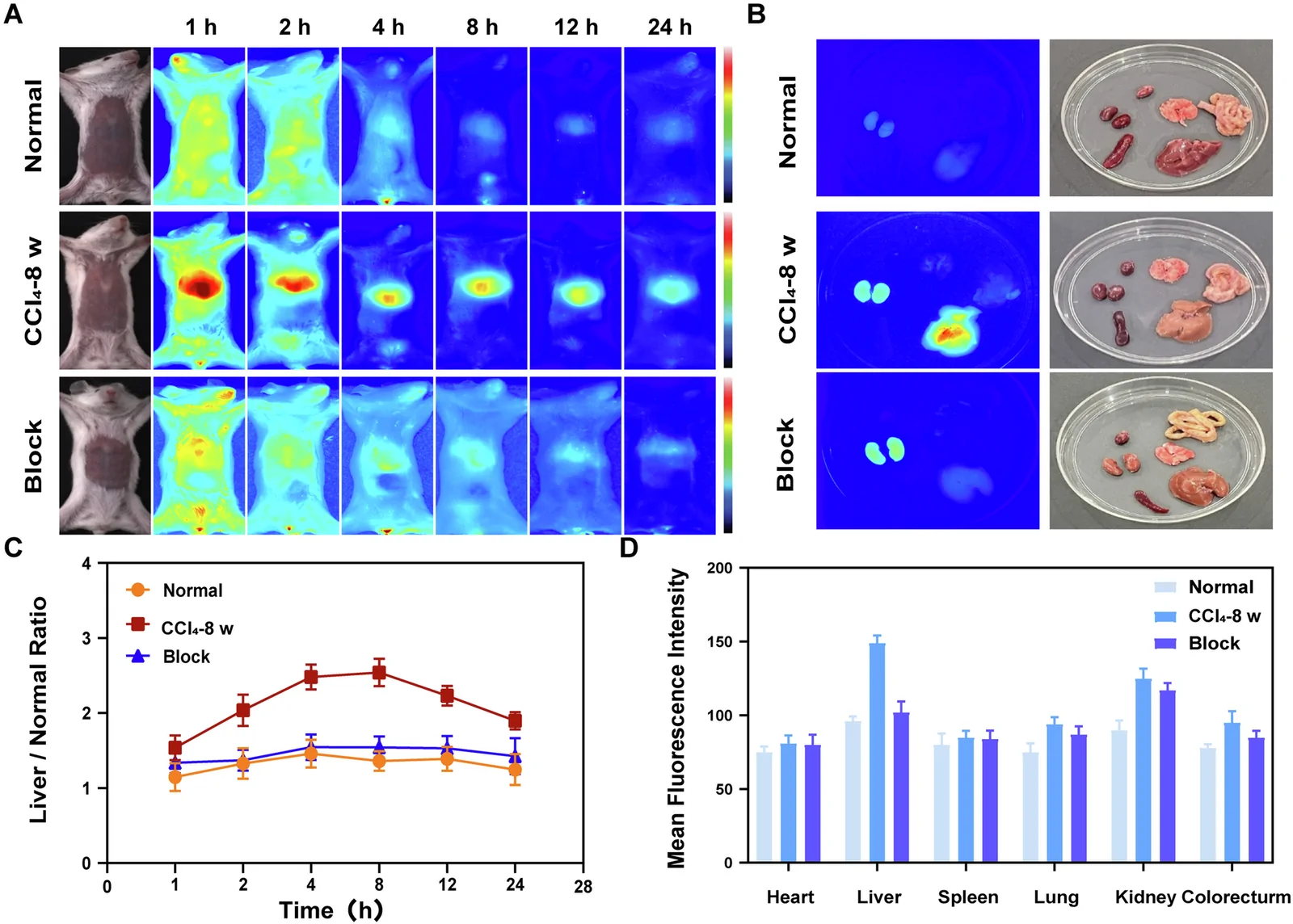
YQ-HSP-6 in the CCl4-induced liver fibrosis model. **A** In vivo fluorescence imaging of YQ-HSP-6 in normal mice and mice with liver fibrosis. In the blocking group, mice were pretreated with peptide HSP-6 1 h before administration of YQ-HSP-6. **B** Ex vivo fluorescence imaging of major organs after treatment with YQ-HSP-6. **C** Semi-quantitative analysis of the fluorescence ratio between normal and fibrotic liver tissues following YQ-HSP-6 administration. **D** Quantification of mean fluorescence intensity in ex vivo tissues after YQ-HSP-6 treatment. Reproduced with permission^234^.

Wang et al. presented a dual-probe fluorescence imaging technique for precise diagnosis and grading of liver diseases through targeting of HSP70 and PDGFRβ biomarkers. HSP70-targeted probe (YQ-HSP-6) detects stress response and hepatocarcinogenesis, while the second probe (YQ-PDG-1) recognizes fibrogenesis-related pathological processes. Importantly, the combination of HSP70 and PDGFRβ biomarkers makes it possible to diagnose and stage liver diseases, since all three pathologies, acute liver injury, hepatic fibrosis, and hepatocellular carcinoma have overlapping pathologic characteristics limiting the accuracy of biomarker-based disease diagnostics. In other words, the dual-fluorescence imaging strategy allows for precise noninvasive differentiation of various liver diseases at distinct stages (Fig. 40)^234^.

The work by Zhang et al. proposes a liver-targeting NIR fluorescence/photoacoustic probe for hepatic inflammation visualization. NIRF/PA dual-modal probe (hCy-Tf-CA) used for the detection of the superoxide anion (O_2_•−) in early hepatic inflammation. Fluorescence and photoacoustic imaging provide complementary advantages in terms of sensitivity, tissue penetration, and spatial resolution. Moreover, liver-targeting capability allows one to accumulate the probe locally in the tissue to ensure selective visualization. The probe could be applied for noninvasive assessment of liver inflammation and hepatotoxicity for early diagnosis, disease evaluation, and therapy monitoring^235^.

**Fig. 41:**
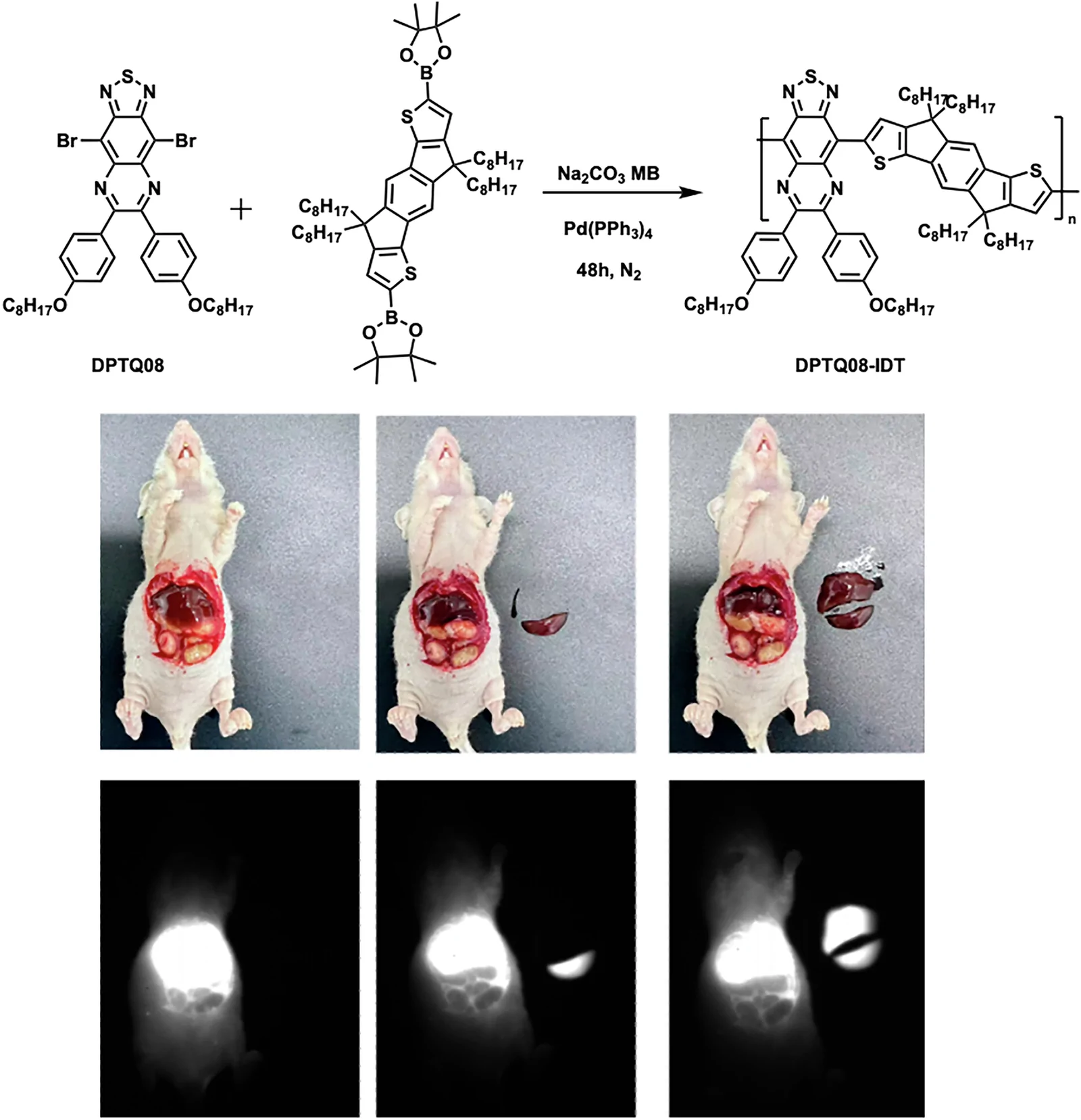
liver-targeted NIR-II fluorescent probe DPTQ08-IDT polymer dots for rapid in vivo imaging. Real-time fluorescence imaging of the liver resection surgical model using DPTQ08-IDT Pdots. DPTQ08-IDT Pdots: 60 μg mL–1. Reproduced with permission^240^.

Another example is provided by Wu et al., who synthesized a novel BODIPY asymmetric fluorescent probe (BDP-NO) capable of detecting both lipid droplets and nitric oxide for the noninvasive visualization of hepatic fibrosis. The dual-targeted design was motivated by the fact that lipids are known to accumulate abnormally under fibrogenic conditions and play important roles in lipid metabolism and redox signaling in the process. In particular, BODIPY-based BDP-NO allows one to report lipid droplets and nitric oxide levels to reveal important features of fibrogenesis development. Biomedical application of BDP-NO involves the fluorescent diagnosis of hepatic fibrosis and monitoring of therapy outcomes^236^. Futher, Wu et al. proposed a novel probe capable of detecting polarity changes in lipid droplets and used it for noninvasive diagnosis of nonalcoholic fatty liver disease (NAFLD). The design of the new BTD-based two-photon NIR probe involves a typical D-π-A motif, ensuring effective polarization changes with the minimum effect of viscosity. Importantly, the polarity of lipid droplets can reveal alterations in their content and metabolism during disease development. In addition, two-photon and NIR emissions enable a deeper imaging with reduced background noise, allowing for better biological compatibility of the probe^237^. Li et al. developed a novel naphthalimide fluorescent probe for detection of lipid droplet polarity in the context of atherosclerosis modeling. Specifically, changes in lipid droplet polarity are associated with alterations in lipid composition and structure, reflecting disease-related lipid remodeling. Since lipid droplets play critical roles in the generation of macrophage foam cells, lipid deposition, and inflammatory processes underlying atherosclerotic plaques, lipid droplet polarity is used as a sensitive marker of disease development in this case. Thus, biomedical implications involve fluorescence analysis of atheroma pathology and lipid metabolic abnormalities in it^238^. Further development of a liver-targeting fluorescent probe by Li et al. can be considered an excellent instrument for precise noninvasive diagnosis of DILI. According to the authors, the probe is based on the probe-lipid nanoparticles coassembly strategy that provides enhanced hepatic accumulation and improved imaging contrast compared to the traditional designs. Considering the heterogeneity and nonspecific nature of DILI-related biochemical markers, the authors aimed at increasing the sensitivity of the method and decreasing nonspecific fluorescence background. Biomedically, this approach could allow for precise early diagnosis of DILI and monitoring of treatment outcomes^239^. A new class of fluorescent probes, folic acid-conjugated DPTQ08-IDT polymer dots, has been designed by Shi et al. in their research as second NIR-II fluorophores for the visualization of the liver tissue and hepatic resection or ischemia/reperfusion injury. This novel imaging agent is based on a donor–acceptor-conjugated polymer, with emission in the NIR-II range around 1050 nm with high brightness, allowing deep penetration in tissues and minimizing any interfering background. By the inclusion of folic acid in the design, the fluorescence dots were able to target the overexpressed folate receptor-α expressed by tumor cells in the liver. Such a strategy allowed for fast targeting of the liver, leading to excellent fluorescence contrast that made possible liver tumor margin visualization in situ and monitoring hepatic circulation during ischemia–reperfusion injury (Fig. 41)^240^.

TXD-3 represents the recently developed naphthalimide-based NIR fluorescent probe that is able to detect changes in lipid droplet polarity in liver injury models. The principle behind the design involves the assumption that lipid droplets experience a number of alterations in morphology, structure, and composition in case of metabolic dysfunction during liver injury. Therefore, the ability to report polar changes allows for revealing additional information about lipid droplets in comparison with conventional lipid staining methods. In in vivo experiments, TXD-3 was applied for the recognition of liver injuries (acute alcohol-induced and NAFLD). The findings support its applicability in disease differentiation^241^. In addition, Xiang et al. developed a series of AIE-active fluorogenic probes (WJ1-WJ4) for fluorescent detection of lipid droplets and diagnosis of NAFLD. The probes involved modifying electron-donating and -withdrawing moieties of the coumarin framework. As a result, they exhibit high lipophilicity and photostability and low cytotoxicity, which allow for quick penetration into cells (~10 seconds) and specific lipid-droplet visualization in cells without washing steps. In addition, this group shows promising potential as NAFLD diagnostics tool^242^. Donor modification allows improving the NIR emission characteristics of fluorescent probes targeting lipid droplets and enabling noninvasive detection of NAFLD. According to Xiang et al., lipid droplets are key participants of hepatic lipid storage and accumulation, characterizing NAFLD. In addition, NIR emission is believed to increase tissue compatibility compared with probes based on shorter wavelength emitters. Thus, according to the authors, lipid-droplet-targeted fluorescent imaging with NIR emission might prove beneficial for NAFLD diagnosis and studies of hepatic lipid metabolic dysfunction^243^.

As an alternative approach, Zou et al. have developed AIE-active fluorescent probe HBA for selective recognition of endogenous/exogenous hydrogen sulfide signaling and detection of nonalcoholic fatty liver disease (NAFLD). Mechanistically, hydrogen sulfide is considered a gasotransmitter playing an important role in regulating hepatic metabolic homeostasis, mitochondria function, redox status, and inflammation. Importantly, changes in the level of endogenous/exogenous H_2_S signal reflect pathology development in NAFLD. Using AIE-active fluorophore, the authors have developed HBA that experiences quenching due to cyclization upon H2S-induced reactions. Biomedical implication lies in fluorescence detection of H2S and its application to NAFLD study^244^.

**Fig. 42:**
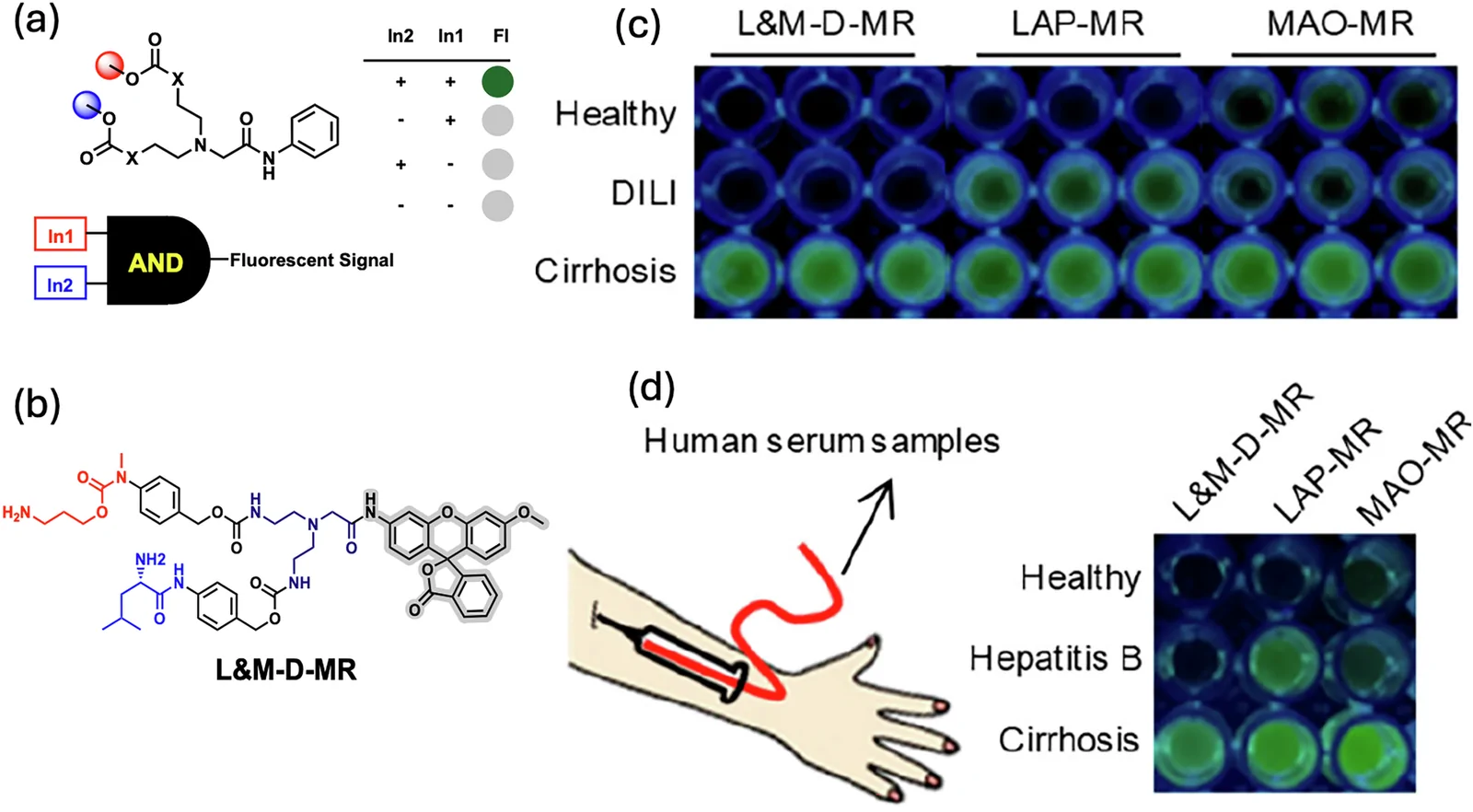
Molecular AND logic-controlled release system L&M-D-MR with monoamine oxidase and leucine aminopeptidase responsive moiety exhibiting fluorescence signal in the serum of cirrhotic mice. **a** AND gate strategy **b** fluorescent AND-gate probe (L&M-D-MR) **c** mouse serum samples treated with L&M-D-MR, and other designed probes LAP-MR, or MAO-MR (50 μM in PBS, pH = 7.4, 0.5% DMSO) in 96-well plates at 37 °C. **d** human serum samples treated with L&M-D-MR, LAP-MR, or MAO-MR (50 μM in PBS, pH = 7.4, 0.5% DMSO) in 96-well plates at 37 °C. Reproduced with permission^85^.

AND-gated fluorescent probes represent a promising instrument for improving disease differentiation by using molecular logic gates, where the fluorescent emission appears due to activation of two biomarkers independently. Such probes are advantageous as they provide increased diagnostic selectivity, as demonstrated by Chen et al. in their recent publication where a dual-responsiveness AND-gate probe (L&M-D-MR) targeting monoamine oxidase and leucine aminopeptidase allowed for discrimination between hepatitis B and cirrhosis by analyzing patients’ blood samples. To validate the in vitro efficacy of the AND gate–controlled drug release, liver cells (QSG-7701) were used for testing the response to the dual enzyme system within a physiological environment. An enhancement in the fluorescence intensity of the AND gate sensor only occurred with dual inputs, confirming the feasibility of converting a dual pathological enzyme input signal into the output of the fluorescence signal from the probe. To validate the application of the probe in vivo and in clinical diagnostics, the AND gate sensor responsive to the two enzymes (MAO and LAP) was employed in mice, followed by testing in human serum samples. The AND gate probe showed excellent response not only in mouse models but also showed capability in differentiating between cirrhosis and hepatitis B-associated human serum samples (Fig. 42)^85^.

Tang et al. developed QD@IR783-TK-FITC, a logical activatable nanoprobe that can be used to evaluate the time-based changes in the fluorescence signal corresponding to HIRI-induced hepatic injury and related systemic inflammation response. From an imaging perspective, the nanoprobe offers a strategy that combines activation based on ROS response with ratiometric NIR-IIb fluorescence imaging for early visualization of oxidative stress in the liver during the early phase of reperfusion injury. This strategy is appropriate since one of the critical molecules involved in the induction of HIRI is overproduction of ROS, which leads to hepatocyte damage and further inflammatory signaling events along with possible multiorgan damage. Importantly, beyond imaging ROS accumulation in the liver, the cleaved fluorescent signals from the nanoreporter can be excreted through urine, allowing for noninvasive monitoring of the systemic inflammation process. Biomedically speaking, the current research is important due to its use of locally imaging ROS with further analysis via noninvasive urinalysis^245^.

**Fig. 43:**
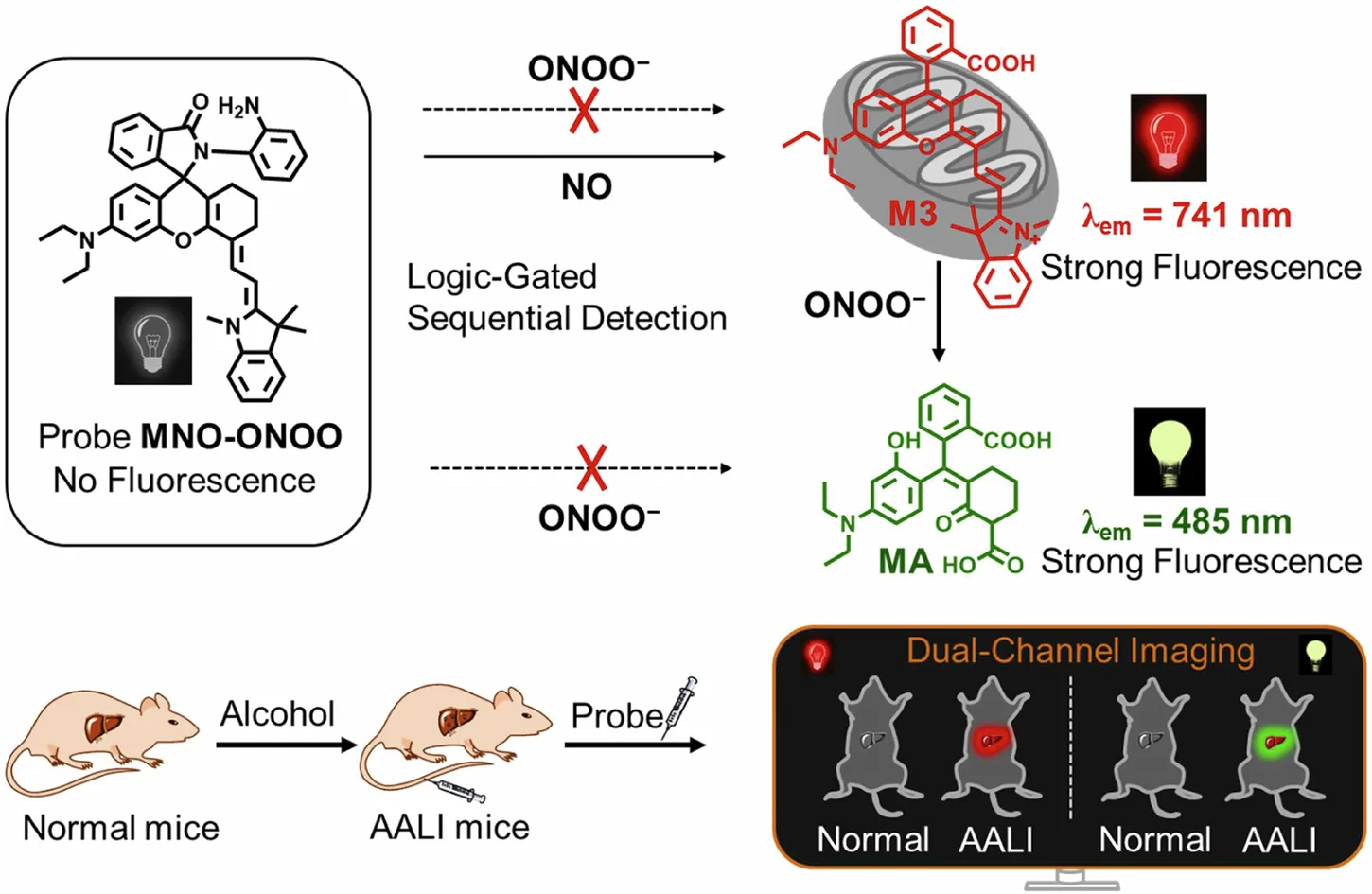
Logic-gated fluorescent probe MNO-ONOO used for the sequential differentiation and detection of NO and ONOO⁻ during Acute alcoholic liver injury (AALI). Probe MNO-ONOO^-^ for logical-gated sequential detection of NO and ONOO^−^ and for imaging of AALI mice. Reproduced with permission^246^.

Zheng et al. describe the development of a logic-gated fluorescent sensor capable of detecting sequential nitric oxide (NO) and peroxynitrite (ONOO⁻) in cases of acute alcoholic liver injury. The importance of such a study in terms of science can be attributed to the fact that acute liver injury induced by alcohol is associated with progressive nitrosative and oxidative stress. In the initial stage, the pathogenesis of acute liver injury is mediated by NO, which is a product of the malfunctioning of the nitric oxide synthase enzyme. Further down the road, NO forms a highly reactive nitrogen oxide – ONOO⁻ – that leads to nitration of tyrosine residues, lipid peroxidation, mitochondrial failure, DNA damage, and liver cell injury. Hence, with the help of their logic-gated probe, Zheng et al. managed to detect the changes from the initial inflammatory/nitrosative signaling phase to later stages when oxidative damage occurs. From the clinical perspective, their research provides evidence that a logic-gated fluorescent sensor is an appropriate tool for investigating the pathological progression of acute alcoholic liver injury (Fig. 43)^246^.

**Fig. 44:**
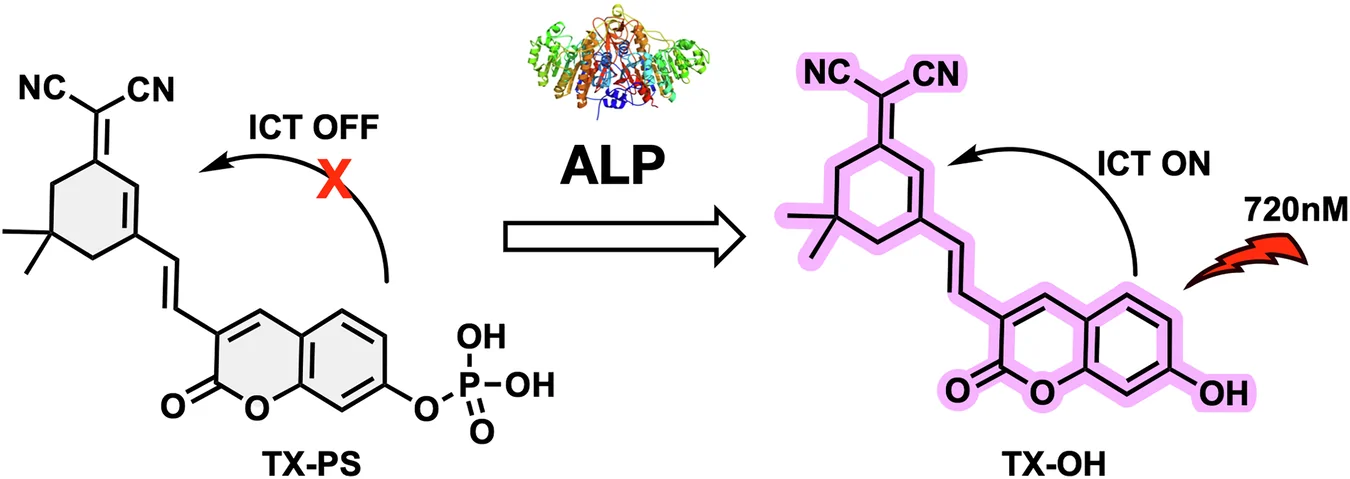
Dicyanoisophorone and coumarin-conjugated TX-PS for the detection of alkaline phosphatase in living cells. Alkaline phosphatase activatable near-infrared fluorescent probe for in-situ diagnosis of cholestatic liver injury.

TX-PS was designed and synthesized for Cholestatic liver injury (CLI) diagnosis by the conjugation of a dicyano-functionalized acceptor unit and coumarin moiety. It was found that the probe was highly specific and sensitive towards Alkaline phosphatase (ALP), and it showed low cytotoxicity and was capable of visualizing the distribution of ALP in cells. Moreover, TX-PS was able to detect significant changes in the concentration of ALP in liver tissues, making it a significant probe for diagnosis (Fig. 44)^247^.

**Fig. 45:**
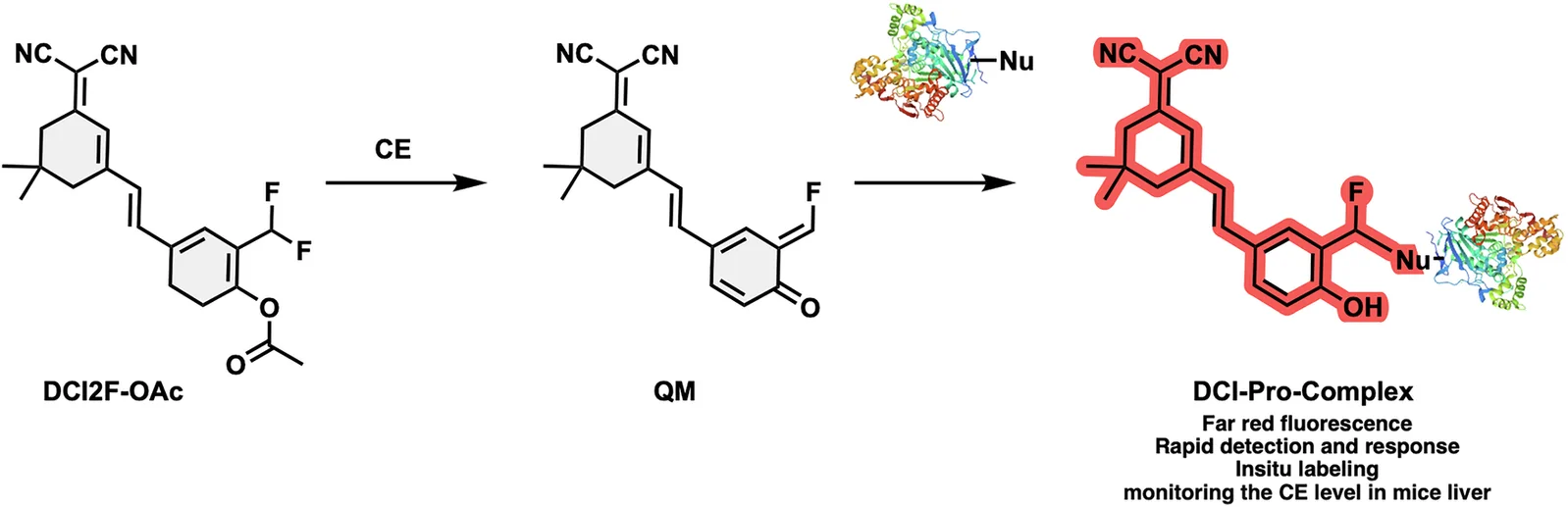
Activatable endoplasmic reticulum targetable proximity labeling far-red fluorescent probe DCI2F-Ac, for real-time detection of carboxylesterases (CEs). Schematic illustration of Intramolecular Charge Transfer (ICT) dual sensing and labeling Far-Red Probe for CEs.

**Fig. 46:**
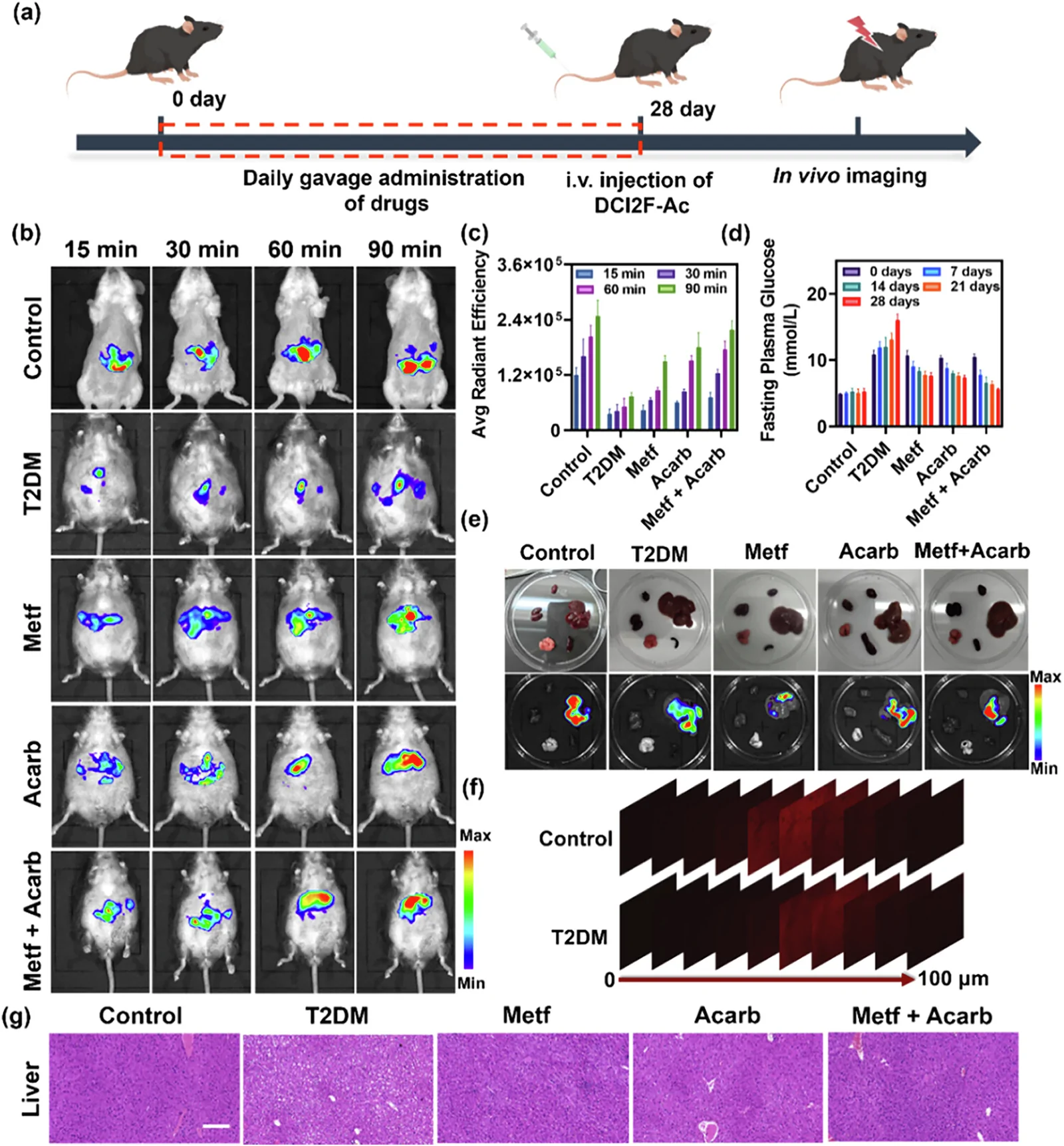
In Vivo Imaging potential of DCI2F-Ac monitoring CEs in Mice of T2DM. **a** Schematic representation of the experimental protocol prepared using FigDraw. **b** In vivo fluorescence images of control, T2DM, and drug-treated mice following intravenous administration of DCI2F-Ac (250 μM, 200 μL). **c** Histogram showing the average radiant efficiency of the mice presented in (**b**). **d** Weekly changes in FPG levels measured at 0, 7, 14, 21, and 28 days during different drug treatments. **e** Fluorescence images and photographs of dissected major organs, including the heart, kidney, liver, spleen, and lung, from the mice shown in (**b**). **f** Fluorescence images of fresh mouse liver slices, including confocal Z-axis scan images acquired at penetration depths of 0, 10, 20, 30, 40, 50, 60, 70, 80, 90, and 100 μm. λex = 488 nm; red channel: λem = 600–700 nm. **g** Hematoxylin and eosin (H&E) staining of liver tissues from control, T2DM, and drug-treated mice. Scale bar: 100 μm. Reproduced with permission^248^.

Xu et al. developed the ER-targeted proximity-labeling far-red fluorescence probe DCI2F-Ac based on dicyanoisophorone for imaging the catalytic activities of carboxylesterases (CEs) in T2DM (Fig. 45). This probe is highly specific and sensitive to CEs, and has the ability to covalently label proteins close to CEs, thus preventing signal diffusion and improving the accuracy of in situ imaging. Using DCI2F-Ac allows researchers to monitor CE activities within cells, study ER stress-induced regulation of CEs, assess the decline in CE activities in liver tissues from T2DM, and investigate the effects of metformin, acarbose, and a mixture of both. Overall, this study reveals the clinical applications of activatable fluorescence probes for metabolic disease diagnostics and drug therapy evaluations. Although the study is focused on T2DM, it is extremely important in the context of liver disease due to the role of CEs in the liver as metabolic enzymes responsible for hydrolyzing esters and other functions. (Fig. 46)^248^.

### Cardiovascular disease detection and image-guided diagnosis

Cardiovascular diseases remain an important problem in terms of global public health, since one of the key causes of complications, such as myocardial infarction and stroke, is atherosclerosis^249^. The development of various imaging modalities for the diagnosis and treatment of atherosclerosis has been actively pursued in few decades; however, most techniques suffer from poor tissue penetration, radiation exposure, complex preparation, photobleaching, low biocompatibility, or lack of specificity^36^. In this context, fluorescent probes have been proposed as effective tools for the early detection of plaque, imaging of vessels in vivo, and monitoring of treatment. As a result of binding with specific targets related to the process of plaque development, which includes inflammatory factors, matrix metalloproteinases, oxidative stress, lipids, and calcifications, fluorescent probes allow obtaining detailed information about plaque pathogenesis, its stability, and predisposition to rupture. Combined with NIR imaging, two-photon imaging, intravascular fluorescence imaging, confocal microscopy, MRI, CT, and other imaging techniques, fluorescent probes provide additional opportunities for diagnostics and therapeutic applications^250,251^.

Given the need for early and accurate diagnosis of atherosclerotic plaques, recent research has increasingly focused on developing dual-input probes that can detect biochemical changes as well as oxidative stress markers. Wang et al. developed a sequence-activated, double-locked two-photon fluorescent probe, C-HBrO-GGT, to realize the early prediction of atherosclerotic plaques by detecting simultaneously γ-glutamyl transpeptidase (GGT) and hypobromous acid (HBrO) simultaneously. In this system, GGT catalyzes the hydrolysis of the probe and releases HBrO-sensitive fluorescence after GGT-mediated hydrolysis. Such sequential activation greatly decreases the risk of false positive detection and makes possible the visualization of oxidative stress and enzymes in the pathogenesis process. Significantly, C-HBrO-GGT is used for the exploration of the CLC-1–HBrO–CAT–GGT cascade in a cell and the prediction of atherosclerotic plaque before the appearance of the plaque or immunofluorescence detection, suggesting its potential as a tool for the early-stage imaging of cardiovascular disease^252^.

**Fig. 47:**
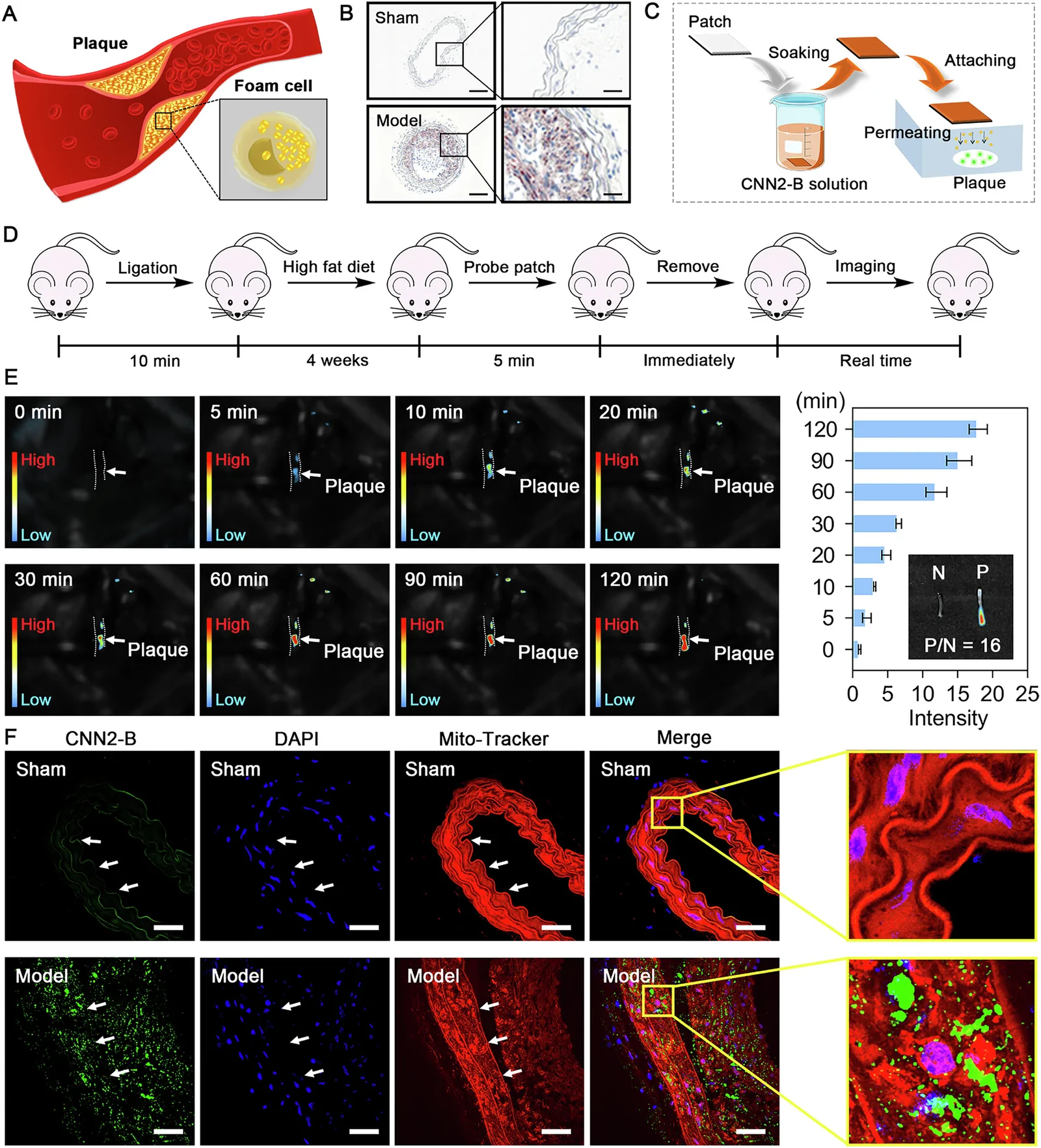
In vivo imaging of an atherosclerotic plaque. **A** Workflow for fluorescence imaging of atherosclerosis in mice. **B** Oil Red O staining images of the carotid artery blood vessel. Scale bars, 100 μm. **C** Schematic representation of in situ probe administration. **D** Schematic illustration of atherosclerosis model establishment and imaging in ApoE mouse. **E** Time-dependent fluorescence imaging data of carotid atherosclerosis using CNN2-B. **F** Confocal imaging data of freeze-sectioned left carotid artery. Green channel: λex = 488 nm; red channel: λex = 555 nm; blue channel: λex = 405 nm. Scale bar = 100 μm. Reproduced with permission^254^.

The dual-biomarker approach has also been adapted for photoacoustic imaging, allowing greater depth of tissue penetration for better in vivo evaluation of inflammation and oxidative damage of plaques. In 2024, Ma et al. reported a unimolecular photoacoustic probe UMAPP for in vivo imaging of neutrophil elastase (NE) and oxidative stress in atherosclerotic plaques. The probe consists of a BODIPY photosensitive scaffold, a NE-substrate chain, and an ONOO⁻/GSH redox-sensitive reporter. As oxidative stress and inflammatory conditions with activated neutrophils are common events in atherosclerotic plaques, UMAPP realizes single-molecular monitoring of multiple pathological behaviors in vivo in atherosclerotic plaques. This discovery is important as photoacoustic imaging allows deep tissue penetration and is superior to conventional fluorescence imaging in vivo^253^. Besides the enzyme- and ROS/RNS-sensitive probes, lipid storage inside plaques has also been used as an indicator of pathology through fluorescent probes targeting lipid droplets. Sang et al. described a peroxynitrite/lipid droplet sequence-activated dual-lock fluorescent probe, CNN2-B, to image atherosclerotic plaques intraoperatively. First, responding to a reactive nitrogen oxide species ONOO^−^, CNN2-B removes the recognition group to cause ICT and to induce coumarin-derived emission, followed by the interaction with lipid droplets, reflecting lipid accumulation in atherosclerotic plaques. Because of the dual-lock design, CNN2-B shows high selectivity, a large fluorescence enhancement factor, and enhanced signal-to-noise ratio (SNR) compared with conventional ONOO⁻-responsive probes, making possible the clear delineation of plaques following local administration in murine models (Fig. 47)^254^.

In addition to enzyme- and ROS/RNS-responsive systems, lipid accumulation within plaques has also been exploited as a pathological imaging feature, particularly through lipid droplet-targeted fluorescent probes. In another report by Sang et al., AND molecular logic gates were proposed as fluorescence “super-enhancers” in designing activatable probes with enhanced SNR. Taking lipid droplets as one input signal and ONOO⁻ as one analyte of interest in disease, the researchers designed probe, CNP2-B. Fluorescence is largely quenched before activation, but after activation with ONOO^−^, it becomes fluorescence-enhanced and moves to lipid droplets. With a remarkably enhanced fluorescence response, CNP2-B successfully images plaques in vivo in mice which clearly showed its importance the role of molecular logic gate designs to improve SNR in disease-diagnosing probes^82^.

**Fig. 48:**
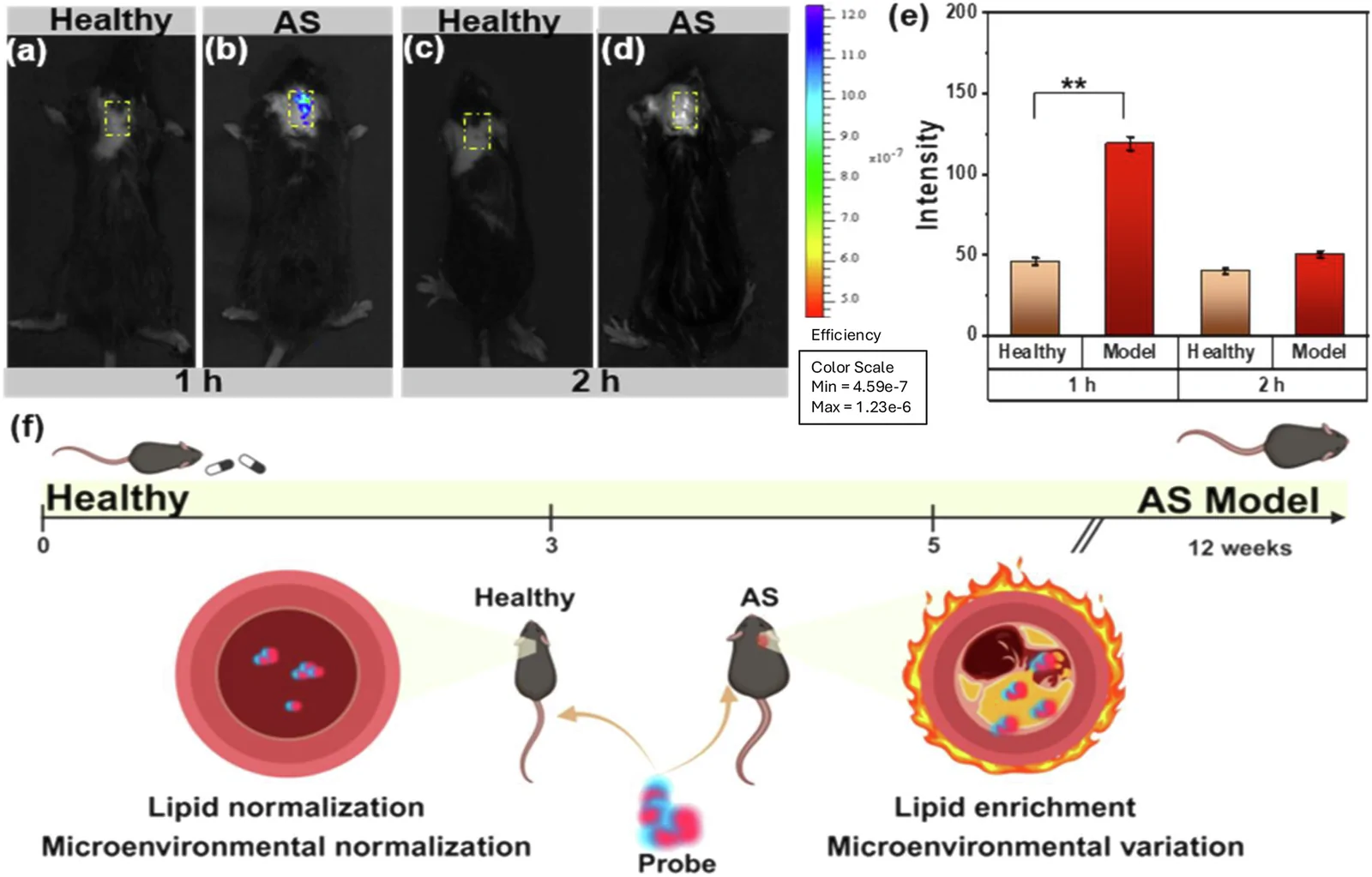
In vivo imaging of AS plaques using benzospiropyran-julolidine-based probe, BSJD. **a**, **c** Healthy mice. **b**, **d** AS mice. **e** Average fluorescence intensities of BSJD in (**a**–**d**). **f** Schematic illustration of BSJD for in vivo fluorescence imaging of AS plaques. Excitation occurred at 560 nm. Reproduced with permission^255^.

Beyond plaque visualization, fluorescent probes have also been designed to monitor cellular transformation events, especially macrophage-to-foam-cell conversion, which is closely associated with plaque growth and instability. Li et al. constructed a dual-color switchable benzospiropyran-julolidine-based probe, BSJD, providing dynamic information of lipid droplets and acidic microenvironment in foam cells in atherosclerotic plaques. Specifically, the probe has dual responsivities in polarity and pH and shows reversible fluorescence emission change. Thus, by using two fluorescence colors, the probe images lipid droplets and acidic microenvironment in the foam cell-forming processes. As foam cells are produced by lipid-loaded macrophages and play important roles in plaque proliferation, the probe serves as a tool for investigating foam cell development and atherosclerosis. BSJD has been shown to be capable of immediately imaging FC plaque locations in atherosclerosis mice with fast pharmacokinetics. Overall, BSJD shows significant potential as a dual-color organelle imaging technique for studying disease-related LD and lysosome alterations and interactions (Fig. 48)^255^.

**Fig. 49:**
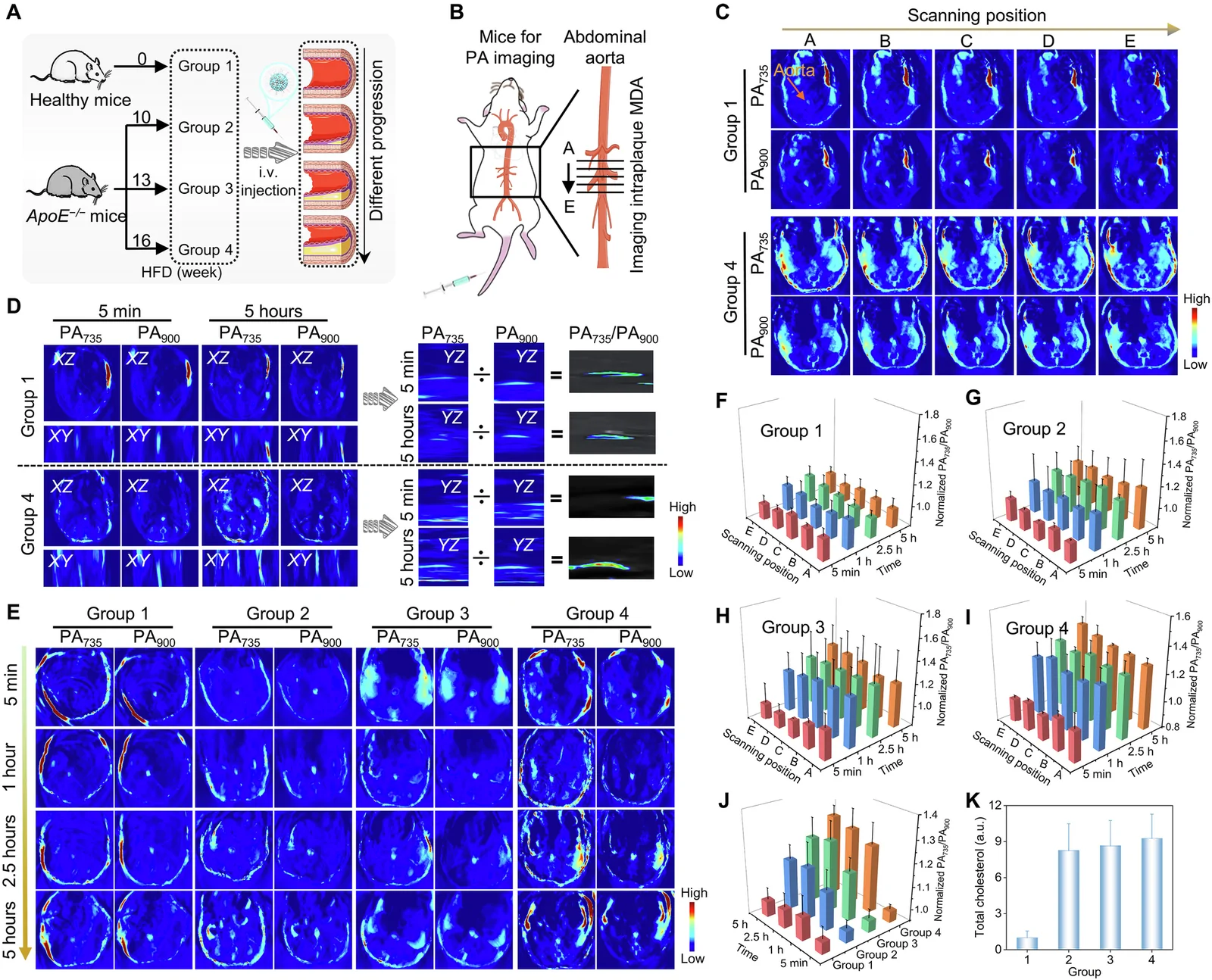
In vivo imaging of atherosclerosis development in mice. **A** Schematic representation of the establishment of different stages of atherosclerosis progression and the corresponding imaging procedure. Group 1: healthy mice; groups 2–4: ApoE−/− mice fed a high-fat diet (HFD) for 10, 13, and 16 weeks, respectively. **B** Graphical representation of PA imaging in living mice. The black area indicates the scanning region, and the black lines denote scanning positions A to E used for data analysis. **C** PA images acquired at different scanning positions (A to E) in group 1 and group 4 mice following injection of the PA nanoprobe. The aortic regions are indicated by arrows. **D** PA images of group 1 and group 4 mice obtained 5 min and 5 hours after injection of the PA nanoprobe in transverse (XZ) and coronal (XY) planes. PA735/PA900 images from the sagittal (YZ) plane of group 1 and group 4 mice were also collected 5 min and 5 hours after PA nanoprobe injection. The PA735/PA900 ratio images were generated by dividing the PA735 images by the PA900 images using Image-Pro Plus. **E** PA images of groups 1–4 mice after injection of the PA nanoprobe. **F**–**I** Quantification of normalized PA735/PA900 ratios in group 1 (**F**) group 2 (**G**) group 3 (**H**) and group 4 (**I**) at different time points and scanning positions after injection of the PA nanoprobe, with three mice per group. **J** Quantification of normalized PA735/PA900 ratios in groups 1–4 at different time points after injection of the PA nanoprobe, including data from five scanning positions per mouse; three mice per group, means ± SD. **K** Relative total cholesterol content in groups 1–4 mice. Reproduced with permission^257^.

Cardiovascular fluorescent imaging has progressed beyond plaques to include thrombosis, involving mitochondrial dysfunction and reactive nitrogen species involved in vasculature injuries and thrombus formation. Ye et al. developed a dual-response two-photon fluorescent probe for imaging mitochondrial viscosity and ONOO⁻ in thrombosis. It is well-known that oxidative injury, activated platelets, endothelial dysfunction, and ONOO⁻ generation are tightly associated with thrombus formation. Besides, two-photon imaging technique is advantageous for its deeper tissue penetration and less photo-toxicity. By taking advantage of the dual-responsivity and two-photon property, the probe can be applied to study the oxidation and mitochondrial damage in thrombosis models^256^. Ma et al. designed an oxidative stress biomarker-triggered multiplexing system to achieve adjunctive diagnosis of atherosclerosis. Specifically, they utilized malondialdehyde (MDA), an end-product of lipid peroxidation, to develop a near-infrared MDA-responsive small molecule and an acid-unlocked ratiometric photoacoustic nanoprobe to visualize the level of MDA within the plaques, revealing oxidative stress and plaque burden. In parallel, the same chemistry was used for the detection of urinary MDA, which reflects systemic oxidative stress. These dual functionalities allow for the translational application of this probe system for atherosclerosis evaluation (Fig. 49)^257^.

Thus, these examples showcase how novel cardiovascular imaging probes are being designed not only as a simple visualization tool, but also as a multi-functional approach towards enzymatic activity, oxidative stress, lipid deposits, mitochondrial dysfunction, foam cell formation, and plaque burden detection. As indicated in the above summaries, there has been a transition in the field of atherosclerosis diagnosis from simple fluorescent probes to complex molecular tools featuring multiplexing, photoacoustics, logic-gate, and multi-parametric reporting.

## Fluorescent probes in drug delivery systems

**Fig. 50:**
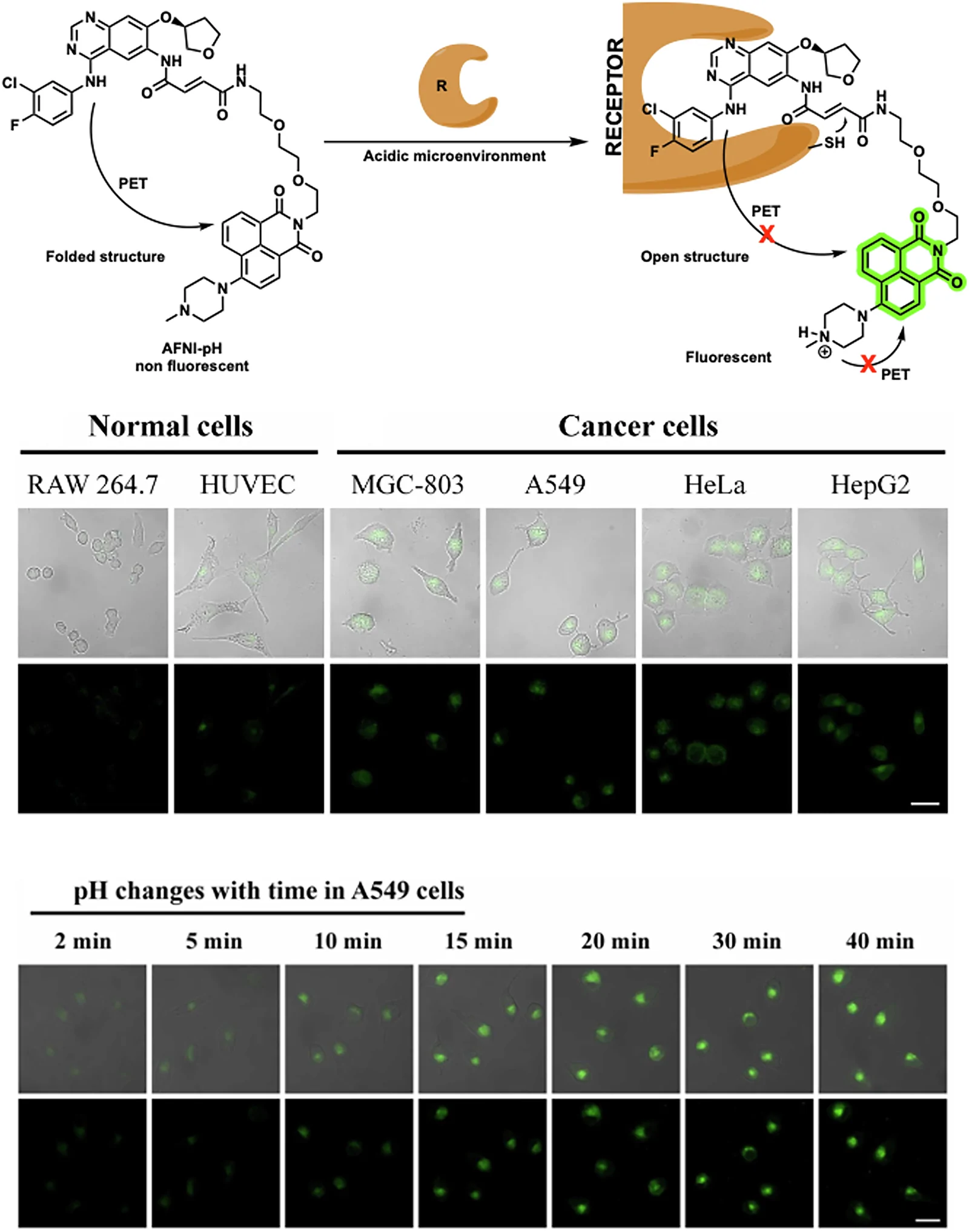
AFNI-pH probe with integrated diagnostic and therapeutic capabilities. Schematic representation fluorescent probe AFNI-pH to monitor pH changes in A549 cells and Fluorescence imaging of probe AFNI-pH (10 µM) in normal and cancer cells. (λ_ex_ = 434 nm, green channel at 475–575 nm. Scale bars: 20 μm. Reproduced with permission^259^.

Fluorescence probes have emerged as essential building blocks in the development of modern drug delivery systems, especially in the rapidly changing field of nanomedicine. A combination of fluorescent markers with therapeutic agents as well as nanoscale delivery systems has allowed for a better understanding of pharmacokinetics, biodistribution, as well as release characteristics in real-time. Some of the notable developments in this regard include theranostic nanocarriers, stimulus-responsive systems, as well as ligand targeting systems. Theranostic nanocarriers such as liposomes, polymeric micelles, dendrimers, as well as solid lipid nanoparticles optimize and modulate pharmacotherapeutic interventions and are designed to co-deliver the drug as well as a fluorescent marker as part of an integrated system. Liposomes have traditionally been applied to administer chemotherapeutic compounds because of their great biocompatibility and broad range of variability with regards to size and surface engineering; they are often incorporated with NIR fluorescent markers like 1,1-dioctadecyl-3,3,3,3-tetramethylindotricarbocyanine iodide (DiR) or ICG^258^. In addition, polymeric micelles and dendrimers have been designed to target chemotherapeutic compounds and fluorescent markers like rhodamine, coumarin, or QDs, enabling multimodal imaging methods (e.g., coupling fluorescence with MRI or PET) alongside therapy. These multimodal carriers provide important pharmacological information while also allowing healthcare practitioners to modulate the therapy in real time depending upon the observed biodistribution patterns, which is at the heart of the vision of personalized medicine. The improvement of the theranostic potential of nanocarriers towards real-time tracking requires developing responsive systems of drug delivery that use precise physiological or pathological conditions as initiators of the release mechanism. A novel theranostic agent, AFNI-pH, is reported to selectively target the epidermal growth factor receptor (EGFR) and human epidermal growth factor receptor-2 (HER2), in combination with a methylpiperazine-functionalized naphthalimide. The agent effectively detected cancer cells based on the drastically higher expression levels of these receptors and the acidic nature of the tumor microenvironment, exhibiting significant cytotoxicity toward A549 cells with high EGFR expression. In addition, this agent possesses the ability to track pH changes during the treatment course, providing a useful tool for studying afatinib resistance and evaluating potential combinatorial therapeutic strategies for future cancer treatments (Fig. 50)^259^.

Similarly, enzymatic reporters that respond to hydrolytic enzymes like matrix metalloproteinases, cathepsins, or caspases are utilized to produce fluorescence upon contact with these biomarkers, which are common in cancer or apoptotic tissue^260,261^. These intelligent reporters not only provide contrast and specificity to targeted therapies but also prevent systemic toxicity by limiting release of the drugs into the targeted tissue, hence minimizing exposure to normal tissue. Another essential part of ensuring accurate delivery of medicaments is played by targeted delivery systems, which use ligand-conjugated fluorescent labels to enable selective targeting of drug-carrying nanoparticles into diseased tissue. By modifying the surface chemistry of nanoparticles with ligands that target receptors overexpressed in pathological tissue—e.g., RGD peptide (less commonly known as Arginylglycylaspartic acid) that target the overexpressed αvβ3 integrin of angiogenic endothelial cells^262–265^, folic acid targeting the folate receptor^266^, or antibodies for HER2 or EGFR—these systems realize very localized distribution into tissue^267,268^. In these targeted delivery systems, the fluorescent label performs twin roles: it authenticates delivery of the carrier into the target tissue and, while so doing, provides visual confirmation of binding and internalization into the target receptor. This behavior is especially useful where activation of the medicament requires internalization or processing in lysosomes. For example, transferrin- or aptamer-modified nanoparticles have been used to transport doxorubicin in combination with fluorescent agents into glioma cells, allowing investigators to validate penetration of the blood–brain barrier (BBB) and delivery into brain parenchymal tissue—an otherwise problematic task^269,270^. More recently, sophisticated techniques like fluorescence molecular tomography (FMT), near-infrared fluorescence, and fluorescence lifetime imaging (FLIM) make quantitative assessment of the signal generated by the probe possible in vivo, allowing for temporal and spatial distribution of the medicament to be dynamically analyzed. Along with the passive targeting mechanism due to the enhanced permeability and retention effect, active targeting by receptor-mediated endocytosis promotes specificity, reduces off-target side effects, and increases therapeutic indices^271^. The incorporation of fluorescent reporters into such delivery systems can be tailored to produce ratiometric responses—shifts in emission spectra arising due to target binding or alterations in ambient conditions—facilitating increased quantification of drug delivery and release. For example, ratiometric FRET-based sensors can monitor concurrent changes in the spatially proximate positions of acceptor and donor dyes due to structural changes in the carrier or cleavage of the spacer, allowing for a very sensitive measure of drug release^272^. Advances have recently resulted in the design of dual- and triple-modal sensors that combine fluorescence with PET, photoacoustic imaging, or Raman spectroscopy, providing complementary data for both diagnostic and therapeutic decision-making.

Along with the use of small-molecule fluorophores, significant attention has been paid into fluorescent nanomaterials, such as QDs, UCNPs, and carbon dots, as robust and photo-stable markers for cellular imaging and tracking. Conjugation of nanomaterials with therapeutic agents facilitate the imaging over an extended period of time thus circumventing the limitations associated with the photobleaching observed with conventional fluorescent probes. Most notably, the biocompatibility and excretion patterns of these agents are currently under intense investigation, with techniques such as PEGylation and biodegradable coatings used to improve their biocompatibility and reduce their uptake by the reticuloendothelial system (RES).The ability to tailor such traits as probe brightness, emission wavelength, and surface characteristics presents great flexibility in the construction of these novel drug-delivery systems^273^. A zwitterionic polymer (ZP) was utilized to form a zwitterionic polymer-drug conjugate (ZPDC) for the simultaneous delivery and co-administration of paclitaxel (PTX) and gemcitabine (GEM). ZP, which preserved the allyl functional groups in addition to pendant sulfonium-benzyl (SB) zwitterions and a Cy5.5 dye subunit, was synthesized using the alkyne-azide click reaction on an alkene/alkyne-functionalized statistical PLA. Whole-body fluorescence imaging on live animals established that ZP was detectable for a six-day window, compared to the lifespan observed for the PEGylated control. This finding points to the greater effectiveness of SB groups over PEG900 at extending the circulation lifetime of polymers^274,275^.

Overall, the evolution of fluorescent probes developments represents the intersection of nanomedicine, chemistry, and imaging, pushing the frontiers of opportunities in targeted therapy. As regulatory authorities become more open to multimodal diagnostic agents, and as clinical applications of fluorescent nanomedicines gradually increase, the addition of these probes to routine therapy protocols holds great promise to enhance the efficacy of treatments, reduce side effects, and usher in an age of intelligent therapeutics.

## Integration with imaging modalities

Fluorescence lifetime imaging microscopy (FLIM) has been of great benefit in the fields of drug delivery and pharmacodynamics, allowing for real-time monitoring of molecular interactions^272^. FRET-based FLIM probes, which have been used to study drug-receptor interactions by measuring the changes in lifetime caused by the binding of the drug molecule to its target^276^. The method has seen widespread use in the characterization of kinase inhibitors, the study of G protein-coupled receptor (GPCR) signaling, and real-time monitoring of drug interactions in cancer cells. Through the incorporation of FRET donor and acceptor pairs in close proximity, any interaction between the drug and target protein affects the efficiency of energy transfer, which in turn is reflected in the shortening of the donor’s lifetime. In this context, FLIM presents an extremely powerful technique for its high sensitivity to the molecular environment and changes in molecular conformation and used for understanding the target engagement, and off-target effects, drug binding kinetics^277^. A pH-sensitive FLIM probes can report the endosomal escape of nanoparticle-encapsulated drugs by tracking shifts in fluorescence lifetime as the probe moves from acidic endosomes to the neutral cytosol. These FLIM-compatible probes are often designed with environment-responsive moieties such as BODIPY^278^, coumarin^279^, or ruthenium complexes^280^, which possess well-characterized lifetimes and high photostability. Notably, FLIM is increasingly being integrated into in vivo imaging methods, including multiphoton and confocal microscopies, to enable high-resolution in vivo imaging. Advances in FLIM endoscopy and FLIM-enhanced microscopes are helping move this technology to the clinic, especially for applications like fluorescence-guided surgery and intraoperative assessment of cancer margins^281^. These systems can discriminate malignant tissue from normal tissue even when there are minimal differences in fluorescence intensity. One of the important aspects of T cells labeled with FLIM-sensitive probes are to understand the in vivo evolution in tumor cells and assess treatment response in real time which undoubtedly extremely crucial for refining immunotherapeutic strategies and identifying early responders or non-responders among patients^282^. Additionally, FLIM, when coupled with sophisticated super-resolution techniques like stimulated emission depletion (STED)^283^, photoactivated localization microscopy (PALM)^284^ structured illumination microscopy (SIM)^285^, and Minimal (fluorescence) photon flux microscopy (MINFLUX)^286^, have effectively utilized these sophisticated probes to achieve sub-diffraction resolution with regard to molecular interactions and the dynamics of drug delivery in living tissue. STED microscopy can provide super-resolved imaging incorporating data about lifetimes.

Taking advantage of contemporary technological developments, scientists have extended their methods to probe complex networks of cellular communication. One new research field involves the use of super-resolution and lifetime imaging methods to study tunneling nanotubes (TNTs) and mitochondrial dynamics in live cells. Present research investigates the mechanisms of communication between cells using TNTs within a live mesothelial H28 cell line. The researchers identified vesicle-like structures and tubular and spherical mitochondria using red and near-infrared dye labeling and lifetime imaging techniques, and mitochondrial dynamics analyses. Novel insights about the structural and functional differences between TNT1 and TNT2 subtypes and their distinct roles in cell-to-cell communication have been revealed by combining advanced FLIM, confocal, and STED methods^287^. With these techniques, the processes involved in TNT synthesis and the methods by which it is transferred across cells, as well as the effects of pharmacological therapies and stressful environments on mitochondrial dynamics and metabolic states, could be easy to follow. The coupling with complementary imaging technologies has significantly improved the efficacy and performance of biomedical diagnostics, introducing an innovative generation of multimodal methods^288^. This multimodal strategy effectively overcomes the intrinsic deficiencies that exist when each modality is used alone. Although fluorescence imaging finds great sensitivity and real-time monitoring of molecular interactions, it often faces disadvantages of limited tissue penetration and poor anatomical representation^289^. On the other hand, image technologies like MRI and CT provide high-resolution anatomy, while PET offers sensitive radiotracer detection throughout the body, though this sacrifices molecular specificity and subcellular resolution that fluorescence imaging provides^289,290^. Therefore, the unification of these technologies into an integrated imaging probe makes it possible to study the processes of diseases from molecular interactions to structures at the organismal level, leading to more accurate diagnosis and improved patient care. The co-joining of dual- and multi-modal image capabilities—combining fluorescence with PET, magnetic resonance imaging (MRI), or photoacoustic imaging—has further extended the use of these probes, enabling complete anatomical, functional, as well as molecular analysis through a single agent^290,291^.

Dual-mode agents have commonly been developed through the encapsulation of various contrast-providing elements into one. One common method is through co-encapsulation of fluorophore with a paramagnetic, or radioisotopic agent into an entity derived from a nanoparticle or a polymer^292,293^. For instance, encapsulation of near-infrared fluorescent dye, e.g., ICG, into liposomes^294^, together with gadolinium (Gd) ion for MRI contrast^295^, enables simultaneous use for both light-transducing tracking and whole-anatomy imaging in the tissue regions further from the surface. These fluorescent nanomaterials with gadolinium have been widely applied in preclinical models of cancer due to the real-time optical feedback each can offer in surgery and aid in MRI-guided localization and volumetry of tumors^296^. Another broadly explored method involves the combination of fluorescence with positron emission tomography (PET), where the fluorophore is conjugated with an already radiolabeled target ligand or encapsulated into PET-trackable nanomaterial. One prominent example of this strategy is use of RGD-capped probes targeting integrin αvβ3, often overexpressed in vascular systems of many tumors^297^. These can be labeled with both fluorophore for immediate visualization and PET isotope, e.g., ^64^Cu or ^18^F, thus permitting exhaustive tracking and aiding visualization of both target and metastatic lesions^298,299^.

The use of fluorescence imaging in addition to computed tomography (CT) is still uncommon, mostly because high atomic number (high-Z) materials are needed to provide contrast in CT. However, AuNPs have proven to be good candidates due to their high X-ray attenuation characteristics and excellent biocompatibility. When surface-modified with fluorescent markers, gold nanoclusters can act as dual-mode agents allowing both fluorescence and CT imaging^300^. For sentinel lymph node mapping, these nanoparticles can provide fluorescence-guided assistance in surgery while enabling preoperative visualization with CT imaging^301^.

The combination of fluorescence imaging with functional and anatomical imaging techniques like MRI, PET, and CT has enormously improved diagnostic functions in modern medicine. These multimodal techniques improve the accurate diagnosis of many diseases by combining molecular specificity, functional evaluation, and anatomical localization into an integrated working scheme. Parallel to this, there is growing usage of artificial intelligence and machine learning algorithms to analyze sophisticated fluorescence image data, to streamline signal extraction processes, and to identify diagnostic patterns with high accuracy. This processing plays an especially critical function in high-throughput drug screens and the early diagnosis of disease.

## Clinical and translational trends

**Fig. 51:**
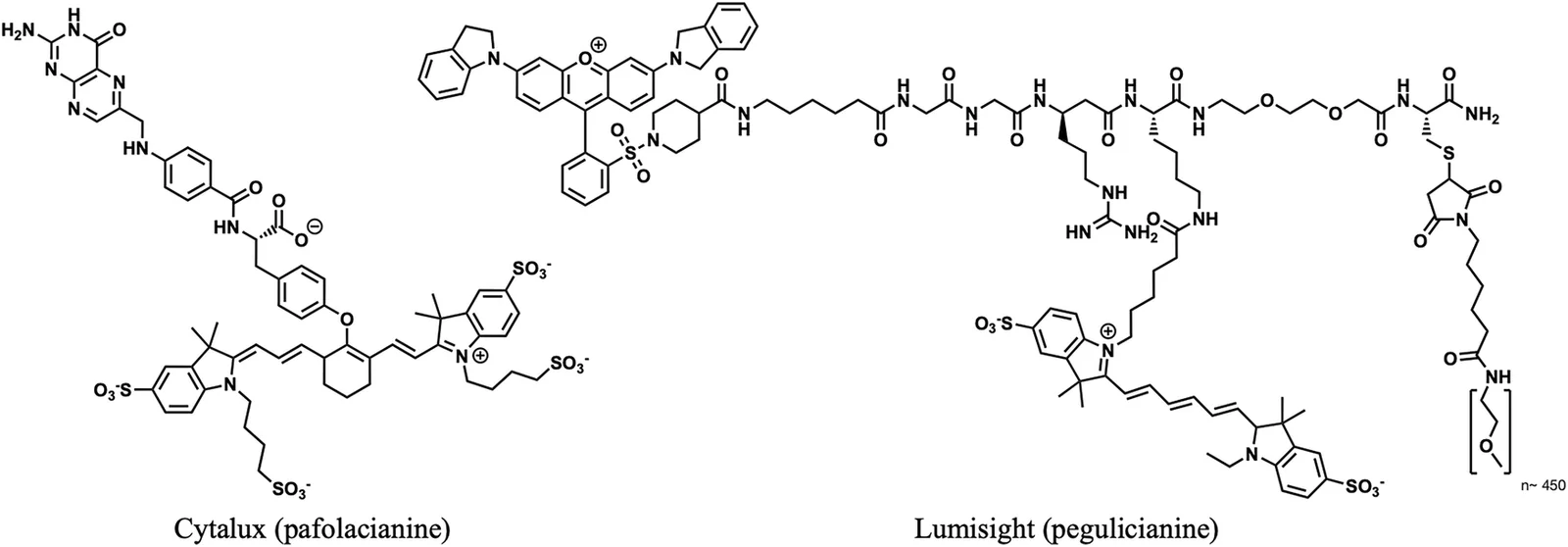
FDA-approved optical imaging agents. Cytalux (pafolacianine) and Lumisight (pegulicianine) for cancer imaging and treatment.

The use of fluorescent probes in the clinical environment has witnessed explosive growth over the last decade, mainly driven by the development of molecular imaging, nanomedicine, and the need for precision medicine therapies^302^. From being largely confined to pre-clinical and basic science studies, fluorescent probes have increasingly found their way into human diagnostic procedures, particularly for the treatment of cancer, surgical guidance, and precise biopsy procedures^289^. One major factor that has propelled the growth of the use of fluorescent probes has been the approval and endorsement by the main regulatory agencies for healthcare, including the United States Food and Drug Administration (FDA) and the European Medicines Agency (EMA) for the use of fluorescent probes. In 2021, the fluorescent imaging probe pafolacianine, which is also known as Cytalux, was approved by the FDA for the treatment of ovarian cancer, which undoubtedly marks a major landmark for the use of these probes (Fig. 51)^303^. The molecule targets the folate receptor-alpha, which is conjugated to a near-infrared dye that allows for real-time margin demarcation during surgery in the instances of epithelial ovarian cancer. This approval marks an important milestone, making it one of the first targeted fluorescent agents approved for use in surgery, opening the door for the release of many more sophisticated agents into clinical use. This fluorescent molecule is used as a diagnostic agent and is injected intravenously before the procedure is initiated^303^.

FDA approved the optical imaging agent Lumisight (pegulicianine, LUM015) together with the Lumicell direct visualization system (DVS), also referred to LumiSystemTM on April 17, 2024 (Fig. 51). The main goal of LumiSystemTM is to aid fluorescence imaging in adult breast cancer patients to help identify the malignant tissue in the resection cavity after removal of the initial specimen in lumpectomy procedures. During a lumpectomy in an adult breast cancer patient, intraoperative application of Lumisight (pegulicianine) aids identification of any remaining cancerous tissue which might still exist in the excision cavity when the main specimen has been removed. Lumisight is a light-emitting imaging agent intravenously administered before the surgical procedure. The Lumisight system is used with the Lumicell DVS or other FDA-approved systems to perform fluorescence imaging and alternative systems approved by the FDA to deliver pegulicianine in breast lumpectomy procedures. The safety and efficacy of Lumisight was tested in a multicenter intra-patient controlled clinical trial (NCT03686215) in breast cancer patients undergoing lumpectomy^304–306^.

The advent of real-time intraoperative imaging has significantly improved the efficacy of oncological surgery and various clinical trials are being carried out to assess the efficacy of fluorescent probes in several oncological and non-oncological applications (Table 1). These trials are aimed at enhancing intraoperative visualization, enhancing biopsy precision, and minimizing residual disease after surgery^307^. For example, Tumor Paint BLZ-100 (tozuleristide), which is a peptide-fluorophore conjugate originated from scorpion venom, has been evaluated in Phase II and III clinical trials of pediatric and adult central nervous system tumors. This compound shows target-specific binding towards invasive cells and presents near-infrared fluorescence, which makes it easier to differentiate malignant and normal brain tissue while excising them surgically. BLZ-100 have been instrumental in driving this revolution. BLZ-100 combines with a peptide that preferentially binds tumors and an NIR fluorescent agent, allowing for visualization of cancerous tissue intraoperatively. This real-time feedback promotes more complete resections of the malignancy with minimal damage to surrounding healthy tissue, leading to fewer recurrence cases and better patient outcomes^308^. According to evidence, the agent shows a high level of efficiency as a tumor-imaging agent when used in combination with a NIR imaging system. After intravenous injection of BLZ-100, the CTX conjugate selectively binds and becomes incorporated by cancer cells derived from neuroectodermal tissues. The NIR device makes use of ICG as a fluorophore to produce NIR fluorescence and enables visualization of cancer cells during surgical operations. The first human clinical trial with BLZ-100 which is an intraoperative fluorescent imaging agent, explicitly showed real-time guidance during surgical tumor resection, especially in skin cancer patients. The purpose of this study was to assess the safety, tolerability, and pharmacokinetics of BLZ-100 and to explore the pharmacodynamics of fluorescence imaging of skin tumors. The study involved 21 adult who received doses of 1, 3, 6, 12, and 18 mg, with fluorescence imaging performed before and up to 48 hours after dosing. The evaluation revealed that the BLZ-100 exhibited a good tolerability profile which provide support for the further clinical development of the imaging agent in the field of surgical oncology^308^. Empirical evidence confirms both efficacy and safety of BLZ-100 in a range of cancer indications that includes gliomas and pediatric central nervous system tumors. The tumor-targeting agent was developed using the tumor-specific peptide chlorotoxin (CTX), a neurotoxic peptide consisting of 36 amino acids and isolated from the venom of the scorpion Leiurus quinquestriatus, in combination with the fluorophore ICG^309,310^.

**Table 1:** Global clinical trials using fluorescent probes

Trial no.	Disease/condition	Fluorescent probe used	Status
NCT03180307	Ovarian cancer (surgical imaging)	Pafolacianine (OTL38) – FDA-approved as Cytalux	Completed
NCT04241315	Lung cancer (thoracic surgery)	Pafolacianine (Cytalux)	Completed
NCT03686215	Breast cancer (lumpectomy surgery)	Pegulicianine(Lumisight) FDA-approved in 2024	Completed
NCT00241670	High-grade glioma (brain tumor resection)	5-ALA (Aminolevulinic Acid) – FDA approved (Gleolan)	Completed
NCT00233402	Non–muscle-invasive bladder cancer	Hexaminolevulinate (HAL) – FDAapproved (Cysview)	Completed
NCT05168839	Colorectal cancer (left colon/rectal resection)	Indocyanine Green (ICG)	Recruiting
NCT03050879	Gastric cancer (radical surgery)	Indocyanine Green (ICG)	Completed
NCT06755554	Gastric cancer (D1/D2 lymph node dissection)	Indocyanine Green (ICG)	Recruiting
NCT00617981 NCT02112656	Hepatocellular carcinoma	Doxorubicin	Completed
NCT02181075	Liver tumor	Doxorubicin	Completed
NCT06819228	Head and neck cancer	Panitumumab-IRDye800	Recruiting
NCT02415881	Head and neck cancer	Panitumumab-IRDye800	Completed
NCT03733210	Head and neck cancer	Panitumumab-IRDye800	Completed
NCT04511078	Head and neck cancer	Panitumumab-IRDye800	Recruiting
NCT04085887	Brain tumor	Panitumumab-IRDye800	Not yet recruiting
NCT03384238	Pancreatic adenocarcinoma	Panitumumab-IRDye800	Recruiting
NCT02097875	Skin neoplasms	BLZ-100	Completed
NCT07294638	Sentinel lymph node metastasis in early gastric cancer	VE/CX-FTn probe (ferritin-based)	Recruiting
NCT03735680	Pan cancer	ONM-100 (intraoperative fluorescence imaging agent)	Completed
NCT07063693	Brain tumors	ABY-029	Not yet recruiting
NCT02736578	Pancreatic cancer	Cetuximab-IRDye 800CW	Terminated

Similarly, panitumumab–IRDye800CW, an anti-EGFR antibody with an appended near-infrared fluorophore, has shown promising findings in clinical trials with head and neck squamous cell carcinoma and colorectal carcinoma. This fluorescent probe enables intraoperative visualization of EGFR-positive lesions, which acts as an optical “GPS” to help surgeons make accurate resections at the millimeter scale. For breast cancer, fluorescent agents like bevacizumab–IRDye800 and trastuzumab-targeted agents are under investigation for specificity towards VEGF and HER2, respectively, and are leading towards developments with fluorescence-guided mastectomy and sentinel lymph node mapping^311^.

The role of nanoparticles in cancer therapy depends on the vascular heterogeneity of the tumor, the level of immunological infiltration, and the composition of the tumor microenvironment’s extracellular matrix. Some of the most promising clinical trials include research on ThermoDox®, sonoporation, and magnetic nanoparticles among other studies on breast cancer and metastatic pancreatic cancer^312^. Other research is investigating the combination of nanoparticles delivering doxorubicin with agents intended to engineer the vascular properties of breast cancer as well as the delivery of liposomal irinotecan for metastatic pancreatic cancer with the aim of maximizing drug retention using the enhanced permeability and retention (EPR) effect^313^. These studies aim at enhancing drug retention along with selectively targeting particular cancers according to the mechanisms of the EPR effect^314^.

Another significant application is seen in the use of fluorescent probes to aid biopsies. The use of real-time fluorescent imaging has the potential to increase the detection of areas of highest tumor burden or metabolism, resulting in increased diagnostic yield and fewer chances for false negatives. For prostate cancer, for example, current clinical trials are testing the use of PSMA-targeted fluorophores to improve the accuracy of needle positioning in ultrasound- or MRI-guided biopsy procedures. Fluorescence guidance is also under investigation for use in pulmonary nodule localization, gastrointestinal endoscopy, and lymph node sampling, thus offering healthcare practitioners an non-invasive tool for the detection of malignancies that can prove difficult to discern under white light or traditional imaging methods^315–317^.

Despite these developments, the transition of fluorescent probes from laboratory into clinical environments is fraught with challenges, including issues of scalability, safety, and compliance with regulatory standards. Scaling up the synthesis of fluorescent probes for medical use requires strict control of batch uniformity, purity, and formulation stability. The continued development of imaging probes, for example, near-infrared II fluorophores, activatable probes, and multiplexed imaging probes, is likely to lead to the development of next-generation devices that are more sensitive, specific, and able to perform multiple imaging tasks.

## Challenges, opportunities, and future directions

Despite widespread use and substantial advances in fluorescent probes for diagnostic and therapeutic applications, clinical translation and broader deployment remain limited by key challenges, particularly photobleaching and phototoxicity. These can decrease temporal resolution across prolonged imaging sessions, notably in the case of live-cell or in vivo applications. The rapid photobleaching makes maintenance of the signal more complicated, and the usual solution has been to apply more intensive illumination or to increase the duration of the exposure to compensate for this effect. The increased dose of illumination heightens the danger of phototoxicity, which is defined as cellular damage from photo-excited fluorophores that produce reactive oxygen species (ROS) by energy/electron transfer to molecular oxygen. This problem is particularly critical when imaging sensitive tissues such as the brain, eyes, and embryo. In consideration of these limitations, efforts are being made to design biodegradable fluorophores with lower long-term toxicity. To overcome these limitations, continuous research efforts have been directed towards designing photostable fluorophores with high quantum yields and minimized generation of ROS. In addition, fluorescent agents functional at the NIR and NIR-II window have shown increased photostability with improved penetration through tissue, making them more suitable for extended use in vivo. However, an optimal balance among brightness, biocompatibility, and photostability has remained an enormous design challenge, hence driving innovation in the field of probe chemistry. Recent literature precedence suggested that the development and use of fluorescent probes is attaining high specificity for target molecules while minimizing nonspecific accumulation. Fluorescent probes must engage with selective cellular or molecular targets, such as proteins, receptors, enzymes, or organelles, to provide significant diagnostic information. The enzyme inhibition principle has been used in the design of a wide range of modern probes. Environmental influences such as the level of acidity in the medium, temperature changes, and the availability of metallic ions can negatively impact the enzymatic activity and result in subsequent fluorescence quenching. In addition, the sensitivity and reliability of fluorescent signals can be impaired when they are exposed to various interfering species in complex sample matrices. Therefore, the future development of push-pull type fluorescence probes needs to prioritize their anti-interference performance. Nevertheless, a significant number of probes are known to display non-specific binding to the components of the ECM, serum proteins, or non-target tissues, causing noise and potentially misleading signals. This is particularly a problem in vivo, where complex pharmacokinetics, rapid clearance by the kidneys or liver, and immunological interactions can all impact the distribution and localization of the probes. In targeted imaging application, particularly in tumors, the enhanced permeability and retention effect is often utilized to increase passive accumulation of probes in neoplastic tissue^318^. However, to augment targeting specificity, tuning more advanced molecular design strategies, which have involved the development of activatable probes that shows fluorescence only upon response to certain molecular interactions or enzymatic hydrolysis or response are being developed. These intelligent probes are strategically quenched and activated only after interacting with disease-related biomarkers. Probes targeted to matrix metalloproteinases (MMPs), cathepsins, or caspases possess enhanced selectivity towards applications in oncology and inflammation research^319^. In addition, the use of multivalent and co-operative binding methods, where many weak interactions coalesce to provide high affinity and specificity, is also becoming common in probe design. Moreover, surface modification of nanoparticles with stealth coatings, e.g., PEGylation or zwitterion ligands, quenches nonspecific interactions and prolongs circulation time, enhancing the bioavailability and target accumulation of fluorescent probes^320^.

Future developments in fluorescent imaging technologies pertinent to drug delivery and diagnostics are about to enter an unprecedented transformation phase, with expected breakthroughs to revolutionize the field of biomedical science and precision medicine. The intersection of technologies from various fields, such as nanotechnology, synthetic chemistry, bioengineering, and artificial intelligence, is driving the design and manufacturing of next-generation fluorescent probes and imaging technologies with improved brightness, increased biocompatibility, and higher signal-to-background ratio (SBR), translating into tangible benefits with direct therapeutic interventions. For diagnostics, fluorescent imaging is expected to transform from being an addendum tool in the realm of anatomical or pathological description to an active molecular sensor able to monitor real-time pathological processes with the precision of single cells^321^.

An important breakthrough expected in the coming times relates to the routine clinical use of NIR-II (the second near-infrared window, 1000–1700 nm) imaging agents. Although NIR-I imaging (650–900 nm) already increased imaging depth and signal-to-noise ratios, NIR-II imaging offers higher resolution in vivo bioimaging, tissue penetration, reduced light scattering, and minimal autofluorescence, enabling more tissue penetration and with high spatial resolution compared to NIR-I^322^.

Genetically encoded barcodes at single-cell resolution have profoundly impacted experimental biology by enabling high-throughput genetic screening and single-cell lineage tracing. Their combination with DNA-barcoded fluorescence microscopy has enabled multiplexed imaging of molecular activities in individual cells^323,324^. This has particularly sped advances in spatial genomics and transcriptomics by granting unprecedented resolution in the analysis of cellular structures and gene expression in tissues. DNA imaging-derived techniques like MERFISH and seqFISH show great scalability and robustness thus driving advances in systems biology, developmental studies, and the pathophysiology of disease through an understanding of the molecular architecture of biological systems^325–327^.

The convergence of fluorescent diagnostics with artificial intelligence (AI) and machine learning (ML) is starting to produce tremendous breakthroughs^328^. AI-enabled real-time fluorescence data assessments can inform intraoperative decision, guide biopsies with extreme accuracy or predict future recurrence of disease according to patterns detected with molecular imaging. AI can also have critical roles in pharmacological discovery, forecasting the optimal combinations of fluorophores and targets, simulating pharmacokinetics of the probes, and simulating interactions of probes with biological barriers, such as the blood-brain barrier or stroma of tumors. Progressively, imaging systems are becoming inextricably linked to AI and cloud computing, thus enabling centralized, high-throughput data analysis in both clinical and research environments, driving accelerated clinical translation and greater patient benefits^329,330^. The amalgamation of fluorescence sensors and AI has notably improved the efficiency of analytical methods used in the field of environmental analysis. One of the significant advances has been the use of fluorescent graphene quantum dots (GQDs) in combination with ML methods for the detection and determination of Hg²⁺ and Fe³⁺ ions in real water samples^331^.

The field of fluorescent biosensors, including both implantable and wearable devices, represents a growing area of technological advancement^332,333^. These sensing methods, in conjunction with specific fluorophores, have the potential to monitor real-time pathological conditions, including diabetes, sepsis, and cancer recurrence. Fluorescence-based wristwatch-like sensors considerably enhance biomedical research through real-time monitoring of physiological parameters. This skin-worn microsensor technology incorporates the use of fluorophore-functionalized hydrogels, QDs, or responsive polymeric materials to sense biomarker molecules in sweat, interstitial fluids, or other optical methods^334^. For diabetics, the non-invasive method using fluorescent sensors has the capability to measure glucose levels in the body by detecting the presence of glucose in the sweat or interstitial fluids using enzymatic sensing channels or the spectrum shift in the fluorescent spectrum^335^. Diabetic patients can measure the glucose level in the body using a non-invasive technique based on fluorescent sensors that detect the presence of glucose in sweat or interstitial fluids using enzymatic sensing channels or changes in the fluorescence spectrum after molecule binding^336^. These smart sensors can also measure pH levels, sodium, potassium, and chloride ions to assess the hydration state of the body, balance electrolytes, and detect cystic fibrosis progression. These sensors also contain microfluidic channels with integrated LEDs or photodetectors to allow for excitation and detection processes. Wireless signals are relayed to smartphones or cloud servers for enabling personalized monitoring of health and decision support^337^.

In coming years, such “theranostic” agents can employ fluorophores that produce reactive oxygen species to cause photodynamic therapy, block proteins implicated in disease or adjust structural conformation to block signaling pathways linked with diseases. When incorporated with image management, these agents will provide clinicians with real-time views of the progress of therapy and adjust treatments, accordingly, preventing unnecessary patient exposure while maximizing overall therapeutic outcome. The increasing preclinical and clinical proof regarding the effectiveness and safety of NIR fluorophores, enzyme activatable probes, and nanoparticles targeting techniques is now aiding their increased translation towards a higher utility within the biomedical imaging and diagnostics arena. Significant progress has been marked by the introduction of more sophisticated probes, as well as artificial intelligence technology and wearable bio-detecting devices. To date, very few numbers of probes are in direct use for therapeutic applications. Overall, the development of fluorescent imaging should focus on enhancing sensitivity, specificity, and low toxicity. This innovative approach is expected to revolutionize the identification and treatment of complex diseases and to drive the facilitation of new therapeutic approaches in the near future (Table 2).

**Table 2:** Current bottleneck and future directions for fluorescent sensors

Future direction	Current bottleneck	Breakthrough point	Research priorities for future
NIR-II fluorescence imaging	Limited clinically approved NIR-II probes, insufficient brightness, poor wavelength tunability, biocompatibility concerns, and lack of standardized imaging protocols	Brighter organic NIR-II dyes, renal-clearable probes, activatable probes, multiplexed NIR-II imaging, clinically compatible imaging devices	Develop high-quantum-yield organic NIR-II fluorophores, improve clearance and safety, validate large-animal and human-tissue imaging, and move toward fluorescence-guided surgery
AI-integrated fluorescence imaging	Large fluorescence datasets are difficult to interpret; image analysis may suffer from noise, photobleaching, autofluorescence, and poor reproducibility	Deep-learning-based segmentation, signal correction, biomarker extraction, AI-assisted probe design, automated high-content screening	Build standardized annotated datasets, develop explainable AI models, integrate AI with FLIM/super-resolution/NIR imaging, and use AI to predict probe performance
Wearable fluorescence sensors	Difficulty in stable sampling of sweat/tears/interstitial fluid, motion artifacts, photobleaching, calibration issues, and weak clinical validation; optical signals create a significant power drain on batteries	Flexible optical patches, smartphone-readable sensors, ratiometric probes, microfluidic sweat handling, AI-assisted signal correction	Improve long-term sensor stability, validate biomarker correlation with blood values, develop multiplexed wearable fluorescence platforms, and test in real-world patient populations. Using minimally-invasive microneedle arrays will allow for real-time, continuous tracking of drug concentrations, enabling optimized dosages and reduced toxicity.
Genetically encoded fluorescent sensors	Limited spectral range, delivery challenges, phototoxicity, photobleaching, difficulty translating from cells/animals to humans	Red-shifted/NIR genetically encoded sensors, multiplexed sensors, organelle-specific reporters, improved brightness and dynamic range	Expand sensors for metabolites, metal ions, neurotransmitters, and disease-associated signaling; develop multicolor imaging of cellular crosstalk
Theranostic fluorescent probes	Many systems remain proof-of-concept; limited pharmacokinetic, toxicity, and clinical validation data	Image-guided drug release, activatable therapeutic probes, fluorescent nanomedicine, photodynamic/photothermal integration	Develop probes that combine diagnosis, treatment, drug-release monitoring, and response evaluation in one platform

## Conclusion

Fluorescent imaging has witnessed tremendous progress in the past two decades, evolving from its initial role as a rudimentary labeling reagent in biochemistry to becoming indispensable in the diagnosis of diseases, image-guided surgery, surveillance therapies, and more recently, targeted treatment delivery. This growth has been stimulated through advancements in areas like synthetic chemistry, nanotechnology, molecular biology, and optical imaging, signaling the state in which fluorescent probes are no longer simply passive markers but active players in clinical decision-making. These developments have yielded an ever-diverse range of probes: small-molecule fluorophores with enhanced photophysical qualities, enzyme-activatable and environment-sensitive probes, as well as NIR and NIR-II fluorophores for extended tissue imaging, complemented by multifunctional nanoparticles with both diagnosis and therapy delivery^338^. Clinically, these have transformed methodologies for diagnosis, therapy, and monitoring of diseases like cancer, infections, and neurological conditions. One such achievement is the FDA approval of pafolacianine (Cytalux), the near-infrared folate receptor-targeting agent, which marks the first molecular imaging agent cleared for intraoperative visualization of ovarian cancer tissue^339^. For example, fluorescent probes targeting overexpressed receptors (such as EGFR, HER2, or folate receptor-alpha) may improve fluorescence-guided surgery by facilitating better intraoperative visualization of tumor and tissue-sparing resections. Studies with BLZ-100 (Tumor Paint), panitumumab-IRDye800CW, among other agents, demonstrate the utility of fluorescence to direct surgical interventions in several types of tumors, such as glioblastoma and head and neck cancer.

Similarly, improvements in enzyme-activated, pH-dependent fluorescent probes have enabled researchers and clinicians to study the microenvironment of tumors, track enzymatic activity linked with metastasis or inflammation^340^. For the diagnosis of infectious diseases, analytical assays are experiencing improvements in speed, cost-effectiveness, and point-of-care testing methods with the coupling of lateral flow assays with fluorescent readout microfluidic systems able to detect viral or bacterial antigens in minutes. During the COVID-19 pandemic, these platforms demonstrated the potential of fluorescent probes to enhance public health surveillance and support decentralized diagnostic activity^341^.

In neurology, the quest to visualize molecular processes in the brain has given rise to fluorescent probes that have the ability to cross the BBB. This strategy allows for the non-invasive (or minimally invasive) monitoring of amyloid-beta and tau, as well as other associated disease biomarkers. Theranostic nanosystems are designed to have both an emitting reporter moiety and an encapsulated therapy, enabling concurrent evaluation of biodistribution, target binding, and therapy efficacy. In particular, these systems are tailored to release their therapeutic payload in response to internal stimuli (e.g., pH, redox, and enzymatic activity) or external stimuli (e.g., light and ultrasound), enhancing space and time control over activation of the drug. This two-modal approach has the potential to boost the therapeutic index of the cytotoxic agents while simultaneously offering an optical readout for the distribution of the probes. Such readouts could allow for the rapid evaluation of the efficiency of the drug delivery, with the potential for real-time dosing and/or administration adjustment. In the future, the combination of fluorescence probes with wearable devices and the assistance of AI algorithms for image analysis will undoubtedly allow for the expansion of the application space for the design of probes and fluorescence imaging modalities.

## Data Availability

No datasets were generated or analyzed during the current study.
